# The Martini 3 Lipidome: Expanded and Refined Parameters Improve Lipid Phase Behavior

**DOI:** 10.1021/acscentsci.5c00755

**Published:** 2025-07-31

**Authors:** Kasper B. Pedersen, Helgi I. Ingólfsson, Daniel P. Ramirez-Echemendia, Luís Borges-Araújo, Mikkel D. Andreasen, Charly Empereur-mot, Josef Melcr, Tugba N. Ozturk, W. F. Drew Bennett, Lisbeth R. Kjølbye, Christopher Brasnett, Valentina Corradi, Hanif M. Khan, Elio A. Cino, Jackson Crowley, Hyuntae Kim, Balázs Fábián, Ana C. Borges-Araújo, Giovanni M. Pavan, Guillaume Launay, Fabio Lolicato, Tsjerk A. Wassenaar, Manuel N. Melo, Sebastian Thallmair, Timothy S. Carpenter, Luca Monticelli, D. Peter Tieleman, Birgit Schiøtt, Paulo C. T. Souza, Siewert J. Marrink

**Affiliations:** † Department of Chemistry, 1006 Aarhus University, Langelandsgade 140, 8000 Aarhus C, Denmark; ‡ Physical and Life Sciences (PLS) Directorate, 4578 Lawrence Livermore National Laboratory, Livermore, California 94550, United States; § Centre for Molecular Simulation and Department of Biological Sciences, 2129 University of Calgary, 2500 University Dr. NW, Calgary, Alberta, Canada T2N 1N4; ∥ Laboratoire de Biologie et Modélisation de la Cellule, CNRS, UMR 5239, Inserm, U1293, Université Claude Bernard Lyon 1, 26911 Ecole Normale Supérieure de Lyon, 46 Allée d’Italie, 69364, Lyon, France; ⊥ Centre Blaise Pascal de Simulation et de Modélisation Numérique, Ecole Normale Supérieure de Lyon, 46 Allée d’Italie, 69364, Lyon, France; # 303091 Heidelberg University Biochemistry Center, Heidelberg 69120, Germany; 7 Department of Physics, University of Helsinki, Helsinki 00170, Finland; 8 Groningen Biomolecular and Biotechnology Institute, Nijenborgh 7, 9747 AG Groningen, The Netherlands; 9 Department of Innovative Technologies, University of Applied Sciences and Arts of Southern Switzerland, Polo Universitario Lugano, Campus Est, Via la Santa 1, 6962 Lugano-Viganello, Switzerland; 10 Department of Applied Science and Technology, 19032 Politecnico di Torino, Corso Duca degli Abruzzi 24, 10129 Torino, Italy; 11 Pharmaceutical Sciences, 128698 AstraZeneca R&D Gothenburg, Mölndal, 431 83, Sweden; 12 Molecular Microbiology and Structural Biochemistry (MMSB), 236346 UMR 5086 CNRS & Université de Lyon, Lyon 69367, France; 13 Institut National de la Santé et de la Recherche Médicale (INSERM), 75654 Paris, France; 14 Department of Theoretical Biophysics, 28273 Max Planck Institute of Biophysics, Max-von-Laue Straße 3, 60438 Frankfurt am Main, Germany; 15 Instituto de Tecnologia Química e Biológica António Xavier, 50106 Universidade Nova de Lisboa, Av. da República, 2780-157 Oeiras, Portugal; 16 505684 Frankfurt Institute for Advanced Studies, Ruth-Moufang-Str. 1, 60438 Frankfurt am Main, Germany

## Abstract

Lipid membranes are central to cellular life. Complementing experiments, computational modeling has been essential in unraveling complex lipid-biomolecule interactions, crucial in both academia and industry. The Martini model, a coarse-grained force field for efficient molecular dynamics simulations, is widely used to study membrane phenomena but has faced limitations, particularly in capturing realistic lipid phase behavior. Here, we present refined Martini 3 lipid models with a mapping scheme that distinguishes lipid tails that differ by just two carbon atoms, enhancing the structural resolution and thermodynamic accuracy of model membrane systems including ternary mixtures. The expanded Martini lipid library includes thousands of models, enabling simulations of complex and biologically relevant systems. These advancements establish Martini as a robust platform for lipid-based simulations across diverse fields.

## Graphical abstract

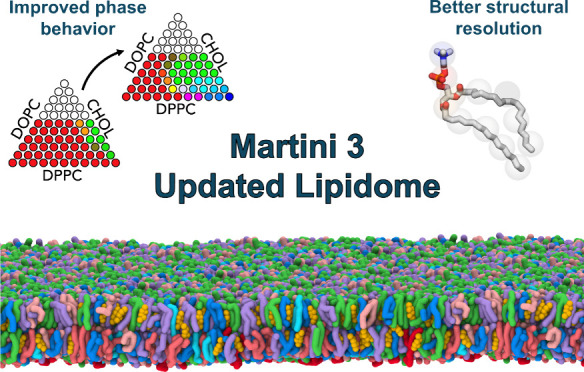

## Introduction

Computational modeling has become an indispensable tool for understanding and predicting the behavior of biological membranes at the molecular level. One of the most impactful approaches in this domain is coarse-graining, which simplifies molecular systems by reducing the number of degrees of freedom, enabling the simulation of larger systems over longer time scales.^1^ Among the various coarse-grained (CG) models, the Martini force field has emerged as a cornerstone,^2^ particularly for studying lipid membranes.^3,4^ Its success lies in balancing computational efficiency with the preservation of essential chemical and physical properties.^2^

Introduced in the early 2000s,^5–7^ the Martini model has continuously evolved, increasing its initial scope from simple lipid bilayers to complex biomolecular systems. With the release of Martini 2,^8^ it became possible not only to expand the library of phospholipid models,^9^ but also to include new lipids such as sterols^10^ and glycolipids,^11,12^ as well as proteins,^13,14^ nucleic acids,^15,16^ carbohydrates,^17^ and other biomolecules.^18,19^ Over the years, Martini has been used in a wide range of membrane-related applications, including domain formation,^20^ complex membrane compositions,^21,22^ membrane remodeling,^23,24^ protein–lipid interactions,^25,26^ and permeability.^27,28^ These studies have provided crucial insights into biological processes at the mesoscopic scale, bridging the gap between atomistic simulations and experimental observations and enabling *in situ* simulations of membranes in the context of a realistic cellular environment.^29,30^

Despite its broad applicability and success, the Martini lipid models have shown certain limitations,^31,32^ particularly in accurately capturing the phase behavior of ternary lipid membranes.^33^ For example, the inability to adequately represent certain lipid mixtures’ liquid-ordered and liquid-disordered phases has been a notable challenge.^34^ Additionally, the Martini 2 model occasionally struggles with reproducing gel and ripple phases, including their temperature phase transitions, lipid packing, and tilting.^35^ Other significant issues have been the model’s difficulty in accurately representing pore formation^36^ and the mechanical properties of membranes.^37^

The recent development of Martini 3 could potentially solve many of these shortcomings.^38^ Martini 3 introduces new bead sizes and chemical types, which combined with well-defined mapping and parametrization strategies could account for the subtleties of lipid head interactions more effectively, including more precise definition of chain lengths and bonded terms. Martini 3 already has a large variety of lipid models to choose from, including common headgroups and diverse tails including completely saturated, mono- and polyunsaturated tails, and cholesterol.^38–40^ Although the current Martini 3 lipid models released showed improved accuracy in a diverse set of applications,^41–46^ they could still be considered prototype models, as they were directly adapted from Martini 2 lipid models.^2,8^

In this work, we present a systematic parametrization and expansion of the whole Martini 3 lipidome, focusing on addressing the limitations of Martini 2.^2,31^ Our approach includes a redefined mapping scheme and integrates both bottom-up fitting of CG parameters to all-atom simulations with CHARMM36 ^47–50^ and top-down validation against experimental bilayer properties. This parametrization strategy, coupled with a new mapping scheme and optimized tail representations, results in the release of thousands of new lipid models. Moreover, these improvements significantly enhance the accuracy of Martini 3 lipid models in capturing key biophysical properties, such as gel–fluid transition temperatures and membrane phase behavior in ternary mixtures. Additionally, we demonstrate several applications of using the reparametrized lipids in modeling complex membrane compositions and topologies important to cellular life, as well as mapping protein–lipid interactions, highlighting the advantages of the expanded Martini 3 lipidome to both academia and industrial applications.

## Results

### Redefining the Lipid Mapping Scheme

To improve the Martini 3 lipidome, we conceived a model that could be extensively verified by experimental data. Due to the ”fuzzy” mapping of Martini 2 lipids,^2,8^ selecting experimental reference data was a major obstacle in benchmarking the model. For example, DPPC (1,2-dipalmitoyl-*sn*-glycero-3-phosphocholine) and DSPC (1,2-distearoyl-*sn*-glycero-3-phosphocholine) are mapped to the same Martini 2 molecule, although experimentally, DPPC and DSPC have significantly different bilayer thicknesses^51^ (DHH 3.46 ± 0.07 nm versus 4.33 ± 0.09 nm at 333 K). This ambiguity complicates fitting to atomistic simulations and validation against experimental data.

We therefore designed a new mapping scheme (Figure 1A) that can differentiate between tails that differ by two carbon atoms (e.g., 16C and 18C tails) based on a center-of-geometry mapping of atomistic structures and including hydrogens in the mapping.^52^ The 16C tails are differentiated from the 18C tails using a small bead that maps 3 carbon atoms immediately after the glycerol ester bead. In contrast, the 18C tail uses a regular-sized bead that maps 5 carbons at the beginning of the tail. In the new mapping scheme, ester groups are mapped together with their adjacent glycerol carbon to a small SN4a bead (Figure 1A). Furthermore, the phosphodiester group is now centered on the phosphorus atom with the *sn*-3 glycerol carbon left out of the mapping (but still implicitly accounted for by the van der Waals radius of the phosphodiester bead), making comparisons of phosphorus distances between CG, AA, and experimental data consistent.

**Figure 1:**
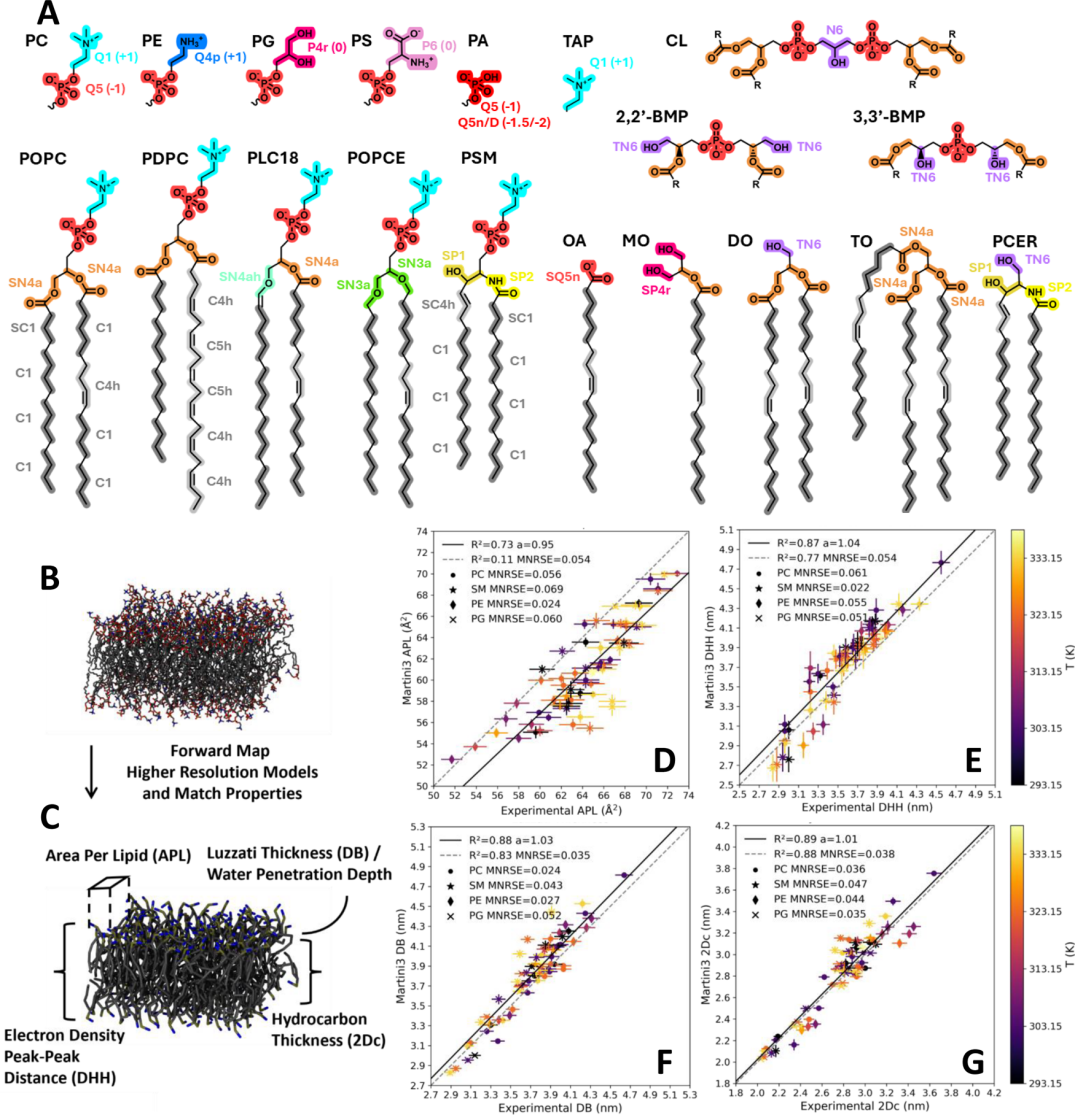
Reproducing structural bilayer properties across major lipid classes. (A) Redefined mapping scheme of Martini 3 lipid models. The AA to CG Martini 3 mapping is shown for several common headgroups, linkers, and full lipids. The different colors indicate the mapped CG bead type and bead charge. Our parametrization strategy relies on first matching bond and angle distributions of higher resolution CHARMM36 lipid models (B), followed by testing a range of emergent bilayer properties like bilayer geometries and phase behavior (C) like area per lipid (APL), electron density peak–peak distance (DHH), water penetration depth/Luzzati thickness (DB), and hydrocarbon thickness (2Dc). To this end, we compiled a large experimental benchmark of lipid bilayers, coined here the “Martini lipid Benchmark” (MIB), and tested the ability of Martini 3 bilayers to reproduce (D) APL, (E) DHH, (F) DB, and (G) 2Dc of the benchmark. We provide a glossary of the lipid abbreviations in the Supporting Information. The data plotted in panel D–G is provided in Table S4.

We also remapped sphingomyelins (SM), where the sphingosine (d18:1) tail now contains an additional bead, compared to the recent parametrization^34^ by Stroh et al., which results in improved phase transition properties. The mapping of polyunsaturated phospholipids was also revised, where the interaction level of the bead now depends on how many double bonds are contained within. Beads that span 1.5 double bonds have their interaction level increased to C5h to differentiate them from the normal double bond type C4h (Figure 1A).

### Reparameterization Strategy

A new set of general parameter building blocks was designed to fit the updated mapping scheme. Bond lengths now take the modified bead sizes into account and account for whether bonded beads span multiple double bonds, found to have an impact on the average bond length (adjacent double bonds make the center-of-geometry distance between beads shorter). While Martini 2 and Martini v3.0.0 use specific angles over beads containing double bonds (i.e., C-D-C where D is a bead containing a double bond), there is no differentiation between angles where the adjacent beads contain double bonds (e.g., D-D-C or D-D-D).^8^ Our atomistic reference simulations of polyunsaturated lipids suggest that the angle distribution varies depending on the number of double bonds in the lipid tails, and the CG angles were diversified to account for multiple unsaturated beads connected in series. In the new parameter set these cases are fitted independently, improving the angle distribution fit of mono- and polyunsaturated lipids.

The expanded bonded parameter set was first fitted in two stages. First against a diverse set of atomistic reference simulations using the CHARMM36 force field (see simulation details in Methods Section 3.1). The atomistic reference simulations were all mapped to pseudo-CG trajectories (Figure 1,B–C) using our newly mapping scheme (Figure 1A) and equivalent CG simulations were prepared for each system. Then the CG bonded distributions were fitted to the mapped atomistic counterparts in a bottom-up approach, employing manual adjustments and automatic protocols like swarm optimization.^53^

In the second stage of the parametrization, we required that the resulting parameters reproduced a wide range of experimentally derived top-down properties, for example, geometrical features like bilayer area per lipid and thickness, and thermodynamical properties like phase change behavior. This second stage is a highly nonlinear and multidimensional optimization problem that is inherently difficult to solve, especially with a relatively small set of fitting parameters, i.e. general bonded parameters and bead type choices to be used in all lipid-related molecules. Furthermore, due to the simulation turnover time of evaluating top-down properties like phase change behavior, which requires substantial MD sampling, fully automating the second stage was not feasible, and we instead relied on human-in-the-loop decisions and compromises between bottom-up and top-down properties through rational design.

### Benchmarking Martini Against Experimentally Determined Properties of Liquid Bilayers

We conducted an extensive literature search for experimental reference data of bilayer properties including APL and three distinct thicknesses: the bilayer electron density peak–peak distance (DHH), the water penetration depth (DB) also known as the Luzzati thickness, and the hydrophobic thickness of the lipid tail region (2Dc).^4–12^ We observed good agreement between the experimentally derived results and simulations using our new parameter set. For APL, a slight underestimation for PC, PG, and SM lipids by ∼3 Å^2^ was observed (Figure 1D). This is an intentional compromise made to improve gel–liquid transition temperatures and phase separation properties in ternary mixtures. The reproduction of DHH, 2Dc, and DB thicknesses shows that there is a good balance between bond lengths and angles in both the tails and the headgroup region (Figure 1E–G). Lipid order parameters, bending moduli, and APL (Figures S1–S2, Table S1) also compare well between the Martini 3 and CHARMM36 models. The modular and systematic nature of the refined mapping scheme provides access to a large variety of lipid types, showcased by the simulation and characterization of 200 bilayers with common phospholipid headgroup and tail combinations (Figures S3–S4, Table S2), as well as di- and triglycerides (Table S4). The characterization includes analysis of bilayer thickness (PO_4_-PO_4_, DHH, DB, and 2Dc), area per lipid (APL), area compressibility (K_a_), average P2 lipid tail order parameter and lipid diffusion. Combined, the results show that the new lipid models are valid across a broad range of properties and biologically relevant temperatures.

### Improved phase behavior across a wide range of lipid systems

Lipid phase behavior is an important phenomenon *in vivo* and is linked to lateral heterogeneity of lipid bilayers.^17^ One of the major experimental differences between saturated lipids is their bilayer gel–liquid transition temperature (*T*_m_). To quantify *T*_m_, we conducted simulated annealing and seeding simulations (Figure 2A-B) of four saturated phospholipids; DPPC, DSPC, PSM, and SSM. Graphing the Lindemann Index as a function of temperature, phase changes were distinguishable as abrupt changes in the index (Figure 2C–D). Transition temperatures estimated from simulated annealing (Table 1) suggest that Martini 3 overestimates *T*_m_ of the tested lipids by 5–10 K. However, as the annealing simulations displayed significant hysteresis complicating analysis, we also devised an orthogonal protocol of seeding fluid bilayers with a gel phase. Using the seeding setup, we observed transitions that are within 1–4 K of the experimentally determined transition temperatures (Table 1, Figure 2,D).

**Table 1:** Transition Temperatures of Saturated Lipids (K)

	annealing liquid → gel	annealing gel → liquid	annealing combined	seeding	experimental main transition^15,16^ ^a^
DPPC (16:0/16:0)	311.8 ± 2.3	334.0 ± 6.5	323.1 ± 3.5	320.0 ± 4.0	314
DSPC (18:0/18:0)	321.8 ± 1.3	354.4 ± 6.5	338.5 ± 3.5	330.5 ± 2.5	328
PSM (d18:1/16:0)	308.0 ± 1.2	338.8 ± 5.5	323.8 ± 3.0	313.5 ± 2.5	314
SSM (d18:1/18:0)	307.2 ± 1.1	337.2 ± 2.8	322.5 ± 1.5	314.0 ± 1.0	318

a Uncertainty of scanning calorimetry^14^ is ∼2 K.

**Figure 2:**
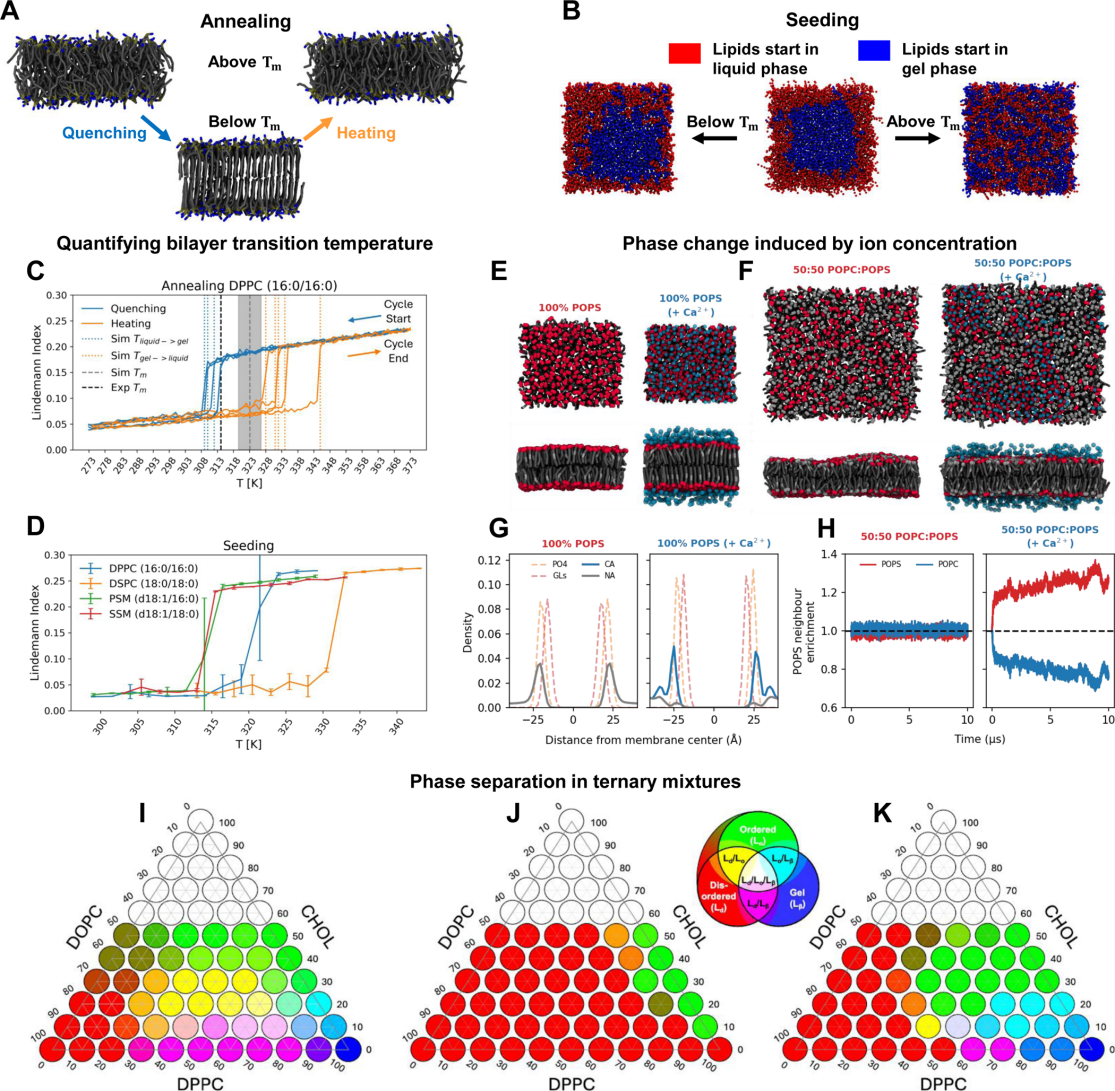
Improved phase behavior of reparametrized Martini 3 lipids. We quantified phase transition temperatures by (A, C) annealing or (B, D) seeding. (C) Lindemann Index plotted as a function of temperature in both the quenching (blue) and heating direction (orange). Liquid → gel and gel → liquid transition temperatures were determined using the largest gradient in the Lindemann Index (blue and orange vertical dotted lines). The experimental reported *T*_m_ is shown as black dotted lines and the *T*_m_ calculated from the annealing simulations is shown as a gray dotted line. (D) Lindeman Index for a series of constant temperature simulations for each lipid type. (E-H) Impact of Ca^*2+*^ on phosphatidylserine-containing bilayers. (E) Liquid→gel transition of POPS bilayer in the presence of Ca^*2+*^. (F) Phase separation in a POPS:POPC 50:50 bilayer in the presence of Ca^*2+*^. (G) Density profile of ions (Na^*+*^, Ca^*2+*^), phosphate (PO_4_), and glycerol linker (GLs) were obtained from simulations of POPS bilayers. (H) Enrichment index of POPC (blue) and POPS (red) lipids obtained from simulations of a POPS:POPC 50:50 bilayer in the presence of Ca^*2+*^. (I–K) Ternary phase behavior for mixtures of DPPC, DOPC, and CHOL from experiments (I), using Martini 2 (J), and using Martini 3 (K).

Lateral heterogeneity can also arise from interactions with divalent cations, particularly Ca^2+^, which plays a crucial role in the function of biological membranes. We conducted a series of tests studying the interactions between Ca^2+^ and anionic phosphatidylserine (PS) bilayers, showing that the reparametrized lipids now can reproduce the phase change response of POPS (16:0/18:1) to an increase in Ca^2+^ concentration (Figure 2E,G) as well as the Ca^2+^-induced phase separation in mixed POPC:POPS bilayers (Figure 2F,H, Figure S5).

Finally, addressing one of the major limitations of Martini 2, we sought to reproduce ternary phase diagrams^18–21^ (Figure 2I) consisting of DPPC (16:0/16:0), DOPC (18:1/18:1), and cholesterol (CHOL) using both Martini 2 (Figure 2J) and Martini 3 (Figure 2K) simulations. Martini 2 shows no phase coexistence in three-component or DPPC/DOPC binary mixtures (Figure 2J), with the phase diagram largely dominated by the L_d_ phase. The reparametrized Martini 3 lipid models capture several features of the experimental phase diagram (Figure 2K). Binary mixtures are now phase-separated in the correct regions, particularly DPPC/CHOL systems. While the L_β_/L_d_ coexistence in DPPC/DOPC mixtures does not extend as far into the lower DPPC region as observed experimentally (Figure 2,I), it is significantly improved. Additionally, much of the L_β_-containing region is reproduced, and we even observed triple-phase L_β_/L_o_/L_d_ systems. Note that a detailed comparison of simulated and experimental phase diagrams is not straightforward, due to differences in system sizes and time scales, as well as changes in hydration levels during phase transitions which are not considered in our simulations. This might also be the reason that the ripple phase is not observed with the current setup.

In addition to the benchmarks presented, we also found improved properties of nonlamellar phases (Figures S6–S7), which expands the applicability of Martini 3 for studying lipid-based drug delivery systems.

### The Martini 3 Lipidome Enables Simulation of Bilayers with Realistic Lipid Composition

It is becoming increasingly clear that modeling of realistic membrane environments is important in the study of transmembrane proteins.^4^ Previously, Ingólfsson et al. have used the Martini 2 force field to build a standardized and simplified mammalian plasma membrane model that contains eight different types of lipids asymmetrically distributed across the leaflets.^21,54^ Here, we adapt this plasma membrane model to the Martini 3 force field (Figure 3) by following a protocol recently presented by Ozturk and co-workers.^55^ This typical mammalian plasma membrane model has an extracellular leaflet enriched in SM lipids with saturated tails (di-C18:0) and cholesterol, whereas its cytoplasmic leaflet mostly contains unsaturated lipids, some of which are composed of negatively charged headgroups, such as PAPS and SAP6. The number of unsaturated bonds per tail is 1.8 times higher in the cytoplasmic leaflet than in the extracellular leaflet, consistent with a recent experimental study on the plasma membranes of human erythrocytes.^56^

**Figure 3:**
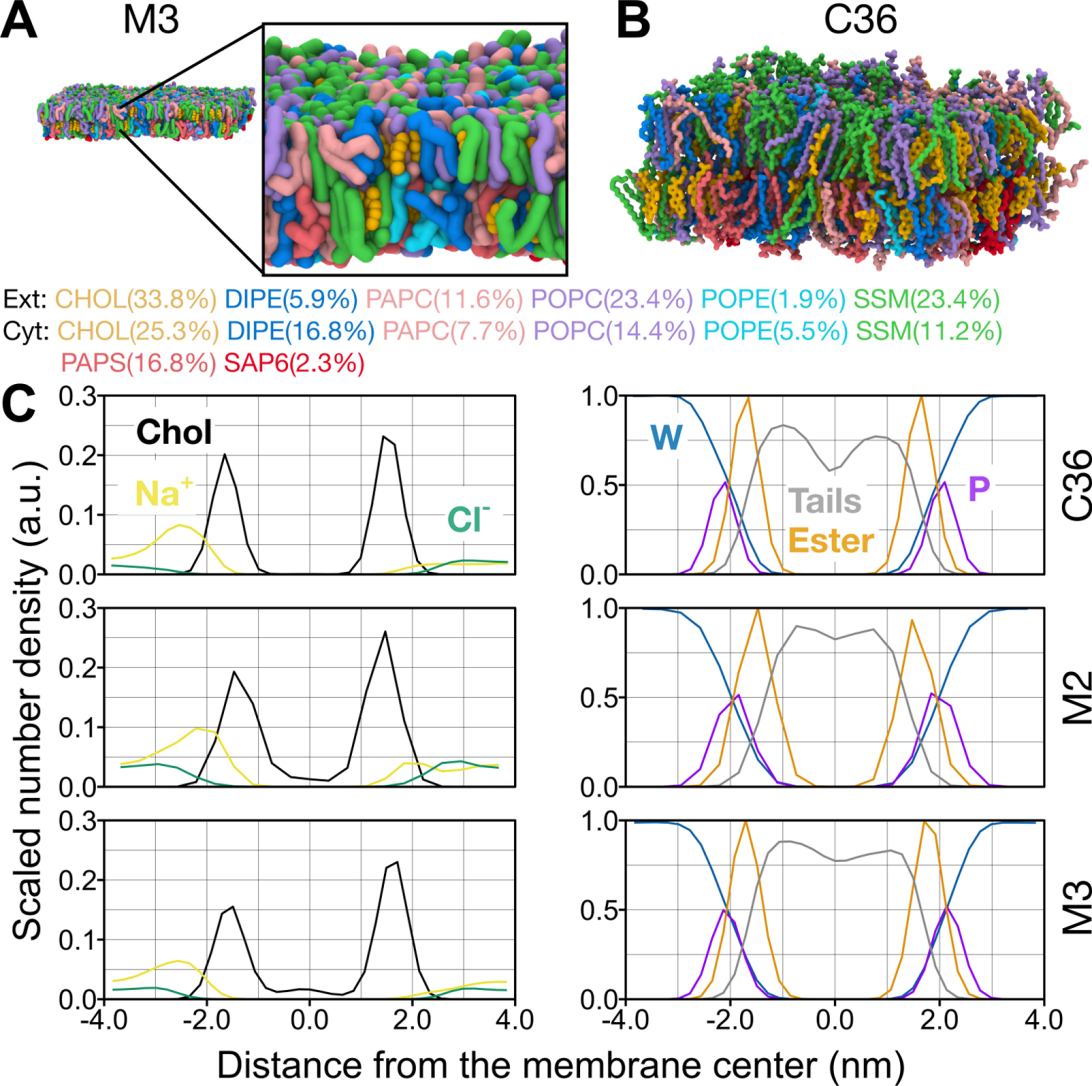
Complex membrane simulations with Martini 3. Snapshots taken from the end of the simulation systems with (A) Martini 3 (M3) and (B) CHARMM36 (C36). Each lipid type is colored differently: CHOL in yellow, DIPE in dark blue, PAPC in light pink, PAPS in pink, POPC in purple, POPE in light blue, SAP6 in cherry, and SSM in green. The snapshots were taken at 20 μs from one of the Martini 3 simulation systems and at 500 ns from one of the CHARMM36 simulation systems. (C) The density profiles of ions (sodium, chloride, and cholesterol (ROH bead in Martini 2/Martini 3 and O3 atom in CHARMM36), water (W), phosphate (PO4 bead in Martini 2/Martini 3 and P atom in CHARMM36), linker (GL1, GL2, OH1, AM2 in Martini 3 and C21 and C31 in CHARMM36), tail beads (C1A, C2A, C3A, C4A, C5A, D1A, D2A, D3A, T1A, C1B, C2B, C3B, C4B, D2B, D3B in Martini 3 and all tail carbon atoms in CHARMM36) along the membrane normal are shown. The top, middle and bottom panels show the data from CHARMM36, Martini 2 (M2), and Martini 3 simulation systems, respectively.

The Martini 2 model of this plasma membrane model was characterized in detail at different levels of complexity in an earlier work by Ingólfsson and co-workers.^54^ Overall, the Martini 3 model has membrane properties compatible with the Martini 2 version. Martini 3 lipid models are now able to distinguish between lipid tails that differ by two carbon atoms which leads to certain differences between the Martini 2 and 3 models of the typical plasma membrane (Table S4). For example, the Martini 3 model has a lower area per lipid for both leaflets, indicating improved lipid packing. The area per lipid in the extracellular leaflet is smaller than in the cytoplasmic leaflet due to the higher proportion of saturated phospholipids as was the case in the Martini 2 model. The membrane thickness, calculated as the phosphate-to-phosphate distance, is found to be higher with the Martini 3 model (4.22 ± 0.01 vs 3.94 ± 0.02 nm). In addition to the slightly faster cholesterol flip-flop rates on average, the equilibrated fractions of cholesterol are also slightly higher (0.59 vs 0.55) in the extracellular leaflet. The phospholipids in the Martini 3 model also diffuse slightly faster in both leaflets and as observed previously with the Martini 2 model, the phospholipids of the extracellular leaflet have slower lateral diffusion rates than those in the PUFA-rich cytoplasmic leaflet (Table S4). Both leaflets of the Martini 3 model are found to be slightly more ordered than those of the Martini 2 model. The extracellular leaflet is more ordered than the cytoplasmic leaflet, as expected due to the asymmetric distribution of lipids with saturated and unsaturated tails (Figure S8).

To validate the Martini 3 plasma membrane model, we built a smaller version of the membrane presented in Figure 3,A and simulated it for 20 μs with the Martini 3 force field, followed by a conversion to an all-atomistic representation compatible with the CHARMM36 force field (Figure 3B) using *ezAlign*,^57^ which was then simulated for 500 ns. The structural properties of the CHARMM36 plasma membrane model are comparable to the Martini 2 and 3 models (Figure 3C). The membrane thickness is 4.191 ± 0.004 nm and is closer to that of the Martini 3 model. The area per lipid values of the leaflets of the CHARMM36 model are found to be 0.517 ± 0.021 nm^2^ in the extracellular and 0.588 ± 0.024 nm^2^ in the cytoplasmic leaflet. Similar to our Martini simulations, using CHARMM36 results in an extracellular leaflet rich in cholesterol and lipids with saturated tails having a lower area per lipid than the PUFA-rich cytoplasmic leaflet.

### Modeling of Multi-Component Membranes Enables Studies of Protein–Lipid Interactions

The Martini model has long enabled the simulation of membrane proteins in realistic lipid environments over extended time scales.^13,58,59^ Here, we assess the ability of the reparametrized Martini 3 lipids to reproduce known lipid–protein interactions across various systems. We focus on major observations, while an extended analysis is provided in the Supporting Information.

In simulations of the Kir2.2 channel with PI­(4,5)­P_2_-enriched bilayers,^40,60^ we observe lipid binding at experimentally identified sites (Figures S9–S10). Similarly, simulations of the ADP/ATP carrier^61,62^ in membranes with cardiolipins reveal lipid binding sites consistent with prior Martini 2 results, albeit with lower lipid occupancies (Figures S9, S11). In general we observe that Martini 3 exhibits faster lipid exchange dynamics and weaker site specificity compared to Martini 2 (Figure S12), likely due to reduced protein–lipid interactions.^31,63^ These differences underscore the need for further benchmarking of lipid residence times and binding site occupation in diverse protein–lipid systems.

We extended our tests to peripheral membrane proteins, including BtPI–PLC,^64–67^ SaPI–PLC,^68,69^ PLC-δ1 PH domain,^70–72^ FGF2,^73–75^ and the Ebola VP40 matrix protein^76,77^ (Figures S13–S17). Binding properties depended strongly on membrane composition: for instance, BtPI–PLC only stably associated with POPC and not DMPC membranes (Figure S13), while SaPI–PLC selectively bound POPG (Figure S14), as expected from experimental results, since SaPI–PLC has no affinity for POPC membranes.^66,68,78^

Our results show that the Martini 3 framework with reparametrized lipids can capture key aspects of membrane-protein interactions across diverse systems. However, the model can be improved, including resolving low lipid occupancies at known experimental binding sites (Figure S9). We expect that ongoing improvements in the Martini 3 protein model and further validation against experimental data are necessary to resolve these issues, and community input will be crucial in guiding future developments. Overall, Martini remains as a flexible platform for modeling proteins and ad-hoc membrane environments.

## Discussion

Despite the considerable advancements achieved with the reparametrized Martini 3 lipidome in accurately representing lipid membranes, several areas require further refinement to broaden its applicability and enhance model accuracy. Here, we outline possible future directions for the Martini lipidome.

First, one of the compromises we made for the presented lipid parameter set, was a small systematic decrease in APL across most lipid types in favor of drastically improved phase transition behavior. One axis requiring further exploration is the properties of the phosphate bead, for which we currently use the Q5 bead, a bead type also assigned to other chemical groups such as carboxylates. This overlap may limit the specificity of ion-induced lipid clustering, particularly in PS- or PIP-rich membranes.^40,79^ It is possible that phosphate could be independently optimized to represent the distinct behavior of phospholipids. Additionally, fine-tuning the interactions between the phosphate and metal ions, like Ca^2+^,^79^ could also be explored to improve the model’s accuracy. Such parametrization could focus on accurate reproduction of experimental APL values while preserving the improved phase transition properties achieved with the current models. Additional refinements of the Martini 3 bead interaction matrix, specifically for the lipid headgroup region, are expected to further improve consistency with experimental and atomistic benchmarks.

Second, while Martini 3 lipids show compatibility with protein–lipid interaction patterns observed with Martini 2 lipids, further validation and optimization is needed. Martini 2 has been extensively tested for diverse protein–membrane systems, but forthcoming improvements in Martini 3 protein models, such as improved side chain thermodynamics and structural flexibility, will require re-evaluation of these interactions. A systematic assessment of the Martini 3 lipidome together with updated Martini 3 protein models will be essential to ensure compatibility across a wide range of systems. In this work, we have established a set of test cases that could be iterated in future parametrization work, and we encourage the broader Martini community to report both positive and negative results of the protein–lipid interactions studied.

Third, while the lipidome described here is significantly expanded to thousands of lipids, it is still incomplete. Important classes of lipids, such as glycolipids, branched lipids, and specialized signaling lipids, remain absent. The inclusion of glycolipids will benefit from the development of Martini 3-compatible sugar parameters.^80,81^ Further optimization of the lipidome would benefit from leveraging automated parametrization tools such as Bartender,^82^ CGCompiler,^34^ and Swarm-CG.^53^ These workflows allow systematic refinement of lipid models while incorporating critical properties, such as phase behavior, directly into the cost function. By automating parameter optimization, the Martini lipidome can be expanded to include new lipids more efficiently, facilitating the exploration of novel lipid structures and a broader range of biological membranes.

Finally, it is important to acknowledge the inherent limitations of coarse-grained modeling, which also apply to the Martini 3 lipidome. For instance, the entropy-enthalpy compensation limits the transferability of the lipid models to state points other than those used in the parametrization process. The entropy-enthalpy trade-off also substantially complicates reproduction of certain delicate processes like bilayer pore formation, where free energy barriers are too high in Martini.^36^ While the reparametrized Martini 3 lipidome slightly improves this property over Martini 2 (Figure S18), the free energy profile of pore formation is still significantly larger compared to atomistic models. Further refinements to phosphate bead types and their interactions with divalent ions may improve the ability to model ion-induced pore formation and even membrane fusion events.^83,84^ Furthermore, although the current Martini lipidome can capture phase transitions quite well, in general, capturing temperature dependent behavior will remain challenging. A temperature-dependent approach, as suggested in earlier works,^85,86^ could mitigate some of these issues and improve the robustness of Martini simulations across a wider range of conditions. Another issue pertains to the artificially enhanced dynamics of CG models. Due to the neglect of atomic degrees of freedom, friction is being reduced, leading to a speedup in the overall dynamics.^87^ For the new Martini lipidome, we do not expect a much different speedup factor compared to previous lipid models, as seen by similar lipid diffusion constants between Martini 2 and 3 models (Figure S1).

## Conclusion

In this work, we presented the new Martini 3 lipidome. The availability of well-calibrated smaller bead types in Martini 3 enabled us to create a larger set of lipid topologies than hitherto possible, meaningfully distinguishing lipids that differ by only two methylene groups. Moreover, by creating new parameter building blocks, novel lipid classes and related surfactant molecules can be easily integrated in a fully compatible manner. Based on the “Martini lipid Benchmark” and an extensive set of tests, we showed a general improvement in reproducing key membrane properties including phase behavior, compared to the previous version of the lipidome. The Martini lipid benchmark suite is not only relevant for the current study but also for benchmarking future lipid force fields irrespective of resolution. We demonstrate how the new Martini 3 lipidome can be used to study a wide range of applications at increasingly realistic *in situ* conditions, from the realistic phase behavior of multicomponent lipid bilayers and mapping of protein–lipid interactions, to the construction of nonlamellar lipid phases for drug delivery, important in both academic and industrial applications.

## Methods

### Atomistic Reference Simulations

The atomistic reference simulation data included three sets of alkanes with a length of 16, 20, and 24 carbon atoms together with 5 different alkenes with a length of 20 carbon atoms, with both mono and polyunsaturated chains, mimicking common lipid tails; glycero-, ether-, and sphingomyelin phospholipid bilayers of pure composition.

The hydrocarbon molecules were parametrized using the CHARMM36 CGenFF force field.^88^ For each hydrocarbon, monoolein (MO, 18:1), or ceramide (PCER, d18:1/16:0), 200 identical molecules were inserted into a cubic box of volume ≈ 5.5 nm^3^. MO and PCER systems were simulated using parameters obtained from the CHARMM-GUI web server.^89,90^ Generic CHARMM36 simulation settings were used: van der Waals nonbonded interactions were switched from 1.0 nm to a cutoff of 1.2 nm. Particle Mesh Ewald^91^ (PME) summation was used for electrostatics with a real-space cutoff of 1.2 nm. Simulations were integrated using 2 fs steps and hydrogen bonds were restrained for all systems using LINCS.^92^ The systems were minimized and equilibrated for 10 ns at 300 K and 1 bar using the Nose-Hoover thermostat (τ_t_ = 1) and Berendsen barostat (τ_p_ = 4),^93,94^ followed by a simulation of 10 ns at 300 K and 1 bar using the Nose-Hoover thermostat (τ_t_ = 1) and Parinello-Rahman (PR) barostat^95^ (τ_p_ = 5). Simulations were performed using GROMACS [Bibr ref96],[Bibr ref97] 2019.4.

The following phospholipid bilayers of pure composition were prepared using the CHARMM-GUI web server: POPC (16:0/18:1), POPE (16:0/18:1), POPG (16:0/18:1), POPS (16:0/18:1), PAPC (16:0/20:4), PDPC (16:0/22:6), DMPCE (e14:0/e14:0), DHPCE (e16:0/e16:0), PLC18 (ve18:0/18:1), PSM (d18:1/16:0), SSM (d18:1/18:0).

The CHARMM36 force field was used for all reference phospholipid simulations.^47,49,50,98^ Each bilayer consists of 200 lipid molecules with a water layer of 2.25 nm on top and bottom of the bilayer. For the anionic PG and PS lipid systems, 200 sodium counterions were added to the solution but otherwise, the systems were simulated without salt. The systems were first minimized and equilibrated in a series of small equilibrations, with gradually weaker restraints on protein and lipids using generic CHARMM-GUI simulation settings. Production simulations were 500 ns long at 310 K (POPC, POPE, POPG, POPS, PAPC, PDPC, PLC18), 323 K (PSM, SSM) or 333 K (DMPCE, DHPCE) and 1 bar using the v-rescale thermostat^100^ (τ_T_ = 1 ps) and Parrinello–Rahman barostat^95^ (τ_p_ = 5 ps) using semi-isotropic pressure coupling . The nonbonded settings were identical to the hydrocarbon simulations. Simulations were performed using GROMACS 2019.4.

### The Martini Lipid Benchmark

We set up lipid bilayers of 200 lipids (100 in each leaflet) in a cubic periodic box of dimensions ≈ 8 nm x 8 nm x 10 nm using *insane*,^9^ matching each lipid type and temperature for each experimental data point in the Martini Lipid Benchmark. The systems were solvated with regular water beads. No ions were added for phosphocholine (PC), phosphoethanolamine (PE), and sphingomyelin (SM) systems. 200 Na^+^ counterions were added to the phosphoglycerol (PG) systems. For each system, the simulation temperature matches the experimental temperature using the v-rescale thermostat (τ_T_ = 1 ps) and we maintain 1 bar pressure using the c-rescale barostat (τ_p_ = 4 ps) using semi-isotropic pressure coupling for both equilibration and production simulations.^99^ Each system was minimized and equilibrated for 50 ns, followed by a 100 ns production simulation. Frames were saved each 1 ns. Simulations were performed using GROMACS 2024.0. Calculation of APL, DHH, DB, and 2Dc is described in the Supporting Information.

### Estimation of Bilayer Transition Temperatures of Saturated Phospholipids

Four systems were built consisting of pure DPPC, DSPC, PSM, and SSM using *insane* ^9^ with 0.15 M NaCl added. The bilayer was minimized and equilibrated for 50 ns at 373 K and 1 bar. Five repeat simulations of annealing consisting of a cycle going from 373 to 273 K to 373 K at a rate of 1 K/10 ns, were conducted. Temperature and pressure were maintained using the v-rescale^100^ thermostat (τ_t_ = 1 ps) and the Parrinello–Rahman^95^ barostat (τ_p_ = 12 ps) using semi-isotropic pressure coupling. Simulations were performed using GROMACS[Bibr ref97] 2024.0.

For the annealing simulations, the Lindemann Index^101^ was calculated for apolar chain beads in blocks of 10 ns, such that each block corresponds to a change of approximately 1 K:

1 $$L=\frac{1}{P}\overset{P}{\underset{p=1}{\sum }}\frac{\sqrt{<{r_{ij}}^{2}>-<r_{ij}{>}^{2}}}{<r_{ij}>}$$

where <*r*_*i* *j*_> is the pairwise distance between bead *i* and bead *j* (*i* ≠ *j*), averaged over the time block of 10 ns, and *P* is the total number of pairs. The liquid → gel and gel → liquid transition temperatures were determined using the largest gradient in the Lindemann Index. The macroscopic transition temperature (*T*_m_) was estimated using eq 2, known as the ”hysteresis method”:^102,103^

2 $$T_{m}=T_{liquid->gel}+T_{gel->liquid}-\sqrt{T_{liquid->gel}{\cdot T}_{gel->liquid}}$$

The seeding setup for evaluation of the bilayer transition temperature was prepared in the following way: Two membranes consisting of 200 lipids were created using COBY^104^ (Coarse-grained System Builder). The two systems were then minimized and equilibrated for 50 ns at 30 K above or below the known experimental transition of the given lipid type, to obtain the APL of the liquid and gel phases, respectively. Utilizing the found APL, COBY was then used to create a membrane consisting of 200 lipids in a liquid phase surrounding a gel patch also consisting of 200 lipids (the gel patch was inserted into the liquid bilayer). The system was minimized and equilibrated for 10 ns with separate temperature baths for the gel patch, simulated at 30 K below the experimental transition, while the surrounding liquid membrane was simulated at 30 K above the experimental transition temperature. Three independent repeats of the equilibration were performed with resampled velocities, resulting in three independent starting structures for the seeds simulations. The seed systems were then each simulated for 500 ns using a constant temperature bath for the entire simulation box, at temperatures ranging from 15 K below the experimental transition temperature to 15 K above, with steps of 2.5 K. The c-rescale barostat (τ_p_ = 4 ps) was used for equilibrations of the initial membranes. For equilibration with separate temperature baths the Berendsen^94^ barostat (τ_p_ = 4 ps) was used. The v-rescale^100^ thermostat (τ_t_ = 1 ps) was used for all simulations. Simulations were performed using GROMACS 2024.0. The Lindemann Index^101^ was calculated using a block of the last 100 ns of each simulation and averaged across the three repeats.

### Ion-Induced Phosphatidylserine Phase Change

The bilayer systems used to investigate ion-induced phosphatidylserine phase change were systematically constructed using COBY.^104^ Pure POPS systems (100% POPS) were prepared in simulation boxes measuring 12 × 12 × 22 nm, while mixed systems (50:50 POPC:POPS) were prepared in boxes of 20 × 20 × 22 nm. For systems containing calcium ions, a ratio of Ca^2+^:PS = 1.5 ratio was used.

Prior to production, the systems underwent 5000 energy minimization steps, followed by equilibration for 10 ns using a 10 fs time step. The production runs for each system were carried out for 10 μs using a 20 fs time step. Throughout the simulations, the temperatures of the lipids and solvent (including Ca^2+^) were independently maintained at 300 K using the v-rescale^100^ thermostat (τ_t_ = 1 ps), and the pressure was kept at 1 bar using the c-rescale^99^ barostat (τ_p_ = 4 ps) with semi-isotropic pressure coupling applied during both equilibration and production phases.^99^ Compressibility was set to 3 × 10^–4^ bar^–1^. As suggested by Kim et al.,^105^ the cutoff distance for the short-range neighbor list was set to 1.35 nm for proper neighbor list updates.

Membrane density profiles were calculated by determining the target particle’s distance to the center of the nearby bilayer and histogramming the z-distance component. The center of the nearby bilayer was defined as the center of geometry of all phosphate (PO4) particles. Lipid neighbor enrichment was calculated with LiPyphilic’s Neighbours module.^106^

### Phase Separation in Three-Component Bilayers

The three-component bilayers to investigate phase separation were systematically constructed using *insane* ^9^ in a 40 × 40 × 10 nm simulation box and solvated with water and 0.15 M NaCl. The resulting systems consisted of ∼6000 lipids and ∼130,000 total particles.

Before production runs, the systems underwent 1500 steps of energy minimization and were subsequently equilibrated for a total of ∼2 ns with increasing time steps (from 1 to 20 fs, see Table S6). The production run for each system was 10 μs. During the equilibration and production runs, LINCS^92^ constraints were used for ring systems and stiff bond constraints; the LINCS order and iteration were set to 12 and 2. The temperatures of lipids and solvent (NA, CL, and W beads) were independently maintained at 297 K with a time constant for coupling (τ_T_) of 1.0 ps, using the Berendsen^94^ and v-rescale^100^ thermostats during the equilibration and production, respectively. The pressure was maintained at 1 bar using the Berendsen^94^ barostat during equilibrations and the Parrinello–Rahman^95^ barostat during the production runs. The compressibility was set to 3 × 10^–4^ bar^–1^. As suggested by Kim et al.,^105^ the cutoff distance for the short-range neighbor list was set to 1.35 nm for proper neighbor list updates. To limit larger-scale bilayer undulations, z-coordinate positional restraints were applied to the PO_4_ beads of the phospholipids in the upper leaflet of the bilayer,^55^ with a force constant of 2 kJ/mol/nm^2^. Note: for smaller bilayer system the periodic boundary condition (PBC) sufficiently limits undulations that the simulated membranes are mostly flat without added positional restraints. However, depending on the lipid mixture used, larger-scale bilayer undulations can affect lipid mixing,^22,107^ complicating analysis, which was not explored here. Determination of phase coexistence is described in further detail in the Supporting Information.

### Simulation of Bilayers with Complex Lipid Compositions

A mammalian plasma membrane model composed of eight different lipid species asymmetrically distributed across the leaflets was built with *insane* ^9^ in a 40 nm × 40 nm × 10 nm simulation box and solvated with water and 0.15 M NaCl (see Tables S5–S6). While maintaining the preset ratio between the phospholipids, 313 randomly selected lipids in the cytoplasmic leaflet were removed to obtain equal surface areas of the leaflets, as described in Ozturk et al.^55^ A total of 4 independent simulation systems were built, each composed of a total of ∼ 128,000 CG beads.

After applying 1500 steps of energy minimization, the simulation system was equilibrated for about ∼ 2 ns with increasing time steps (from 1 to 20 fs, see Table S7 for more details). The production run for each replica was 20 μs. During the equilibration and production runs, LINCS^92^ constraints were used for ring systems and stiff bond constraints; the LINCS order and iteration were set to 12 and 2, respectively. The temperatures of lipids and solvent (NA, CL, and W beads) were separately kept at 310 K with a time constant for coupling (τ_T_) of 1.0 ps, using the Berendsen^94^ and v-rescale^100^ thermostats during the equilibration and production, respectively. The pressure was kept at 1 bar initially using Berendsen^94^ and Parrinello–Rahman^95^ barostats during the equilibration and production steps, respectively. The compressibility was set to 3 × 10^–4^ bar^–1^. The cutoff distance for the short-range neighbor list was set to 1.35 nm for proper neighbor list updates, as suggested by Kim et al.^105^ z-coordinate positional restraints were applied to the phosphate beads of POPC lipids only in the upper leaflet, with a force constant of 2 kJ/mol/nm^2^, to maintain a flat bilayer as explained in earlier work.^55^

For the atomistic simulations, we used the last snapshot of the 20 μs-long coarse-grained trajectory of the smaller plasma membrane patch from an earlier study,^55^ which was converted to an all-atomistic representation using *ezAlign*.^57^ The resulting atomistic systems were composed of ∼27,900 particles. Using these initial coordinates, three independent simulation systems were built, energy-minimized for 500 steps, and equilibrated following the six-step equilibration protocol suggested by CHARMM GUI’s membrane builder^108^ with the modification that the number of steps in each step was doubled. Finally, following the same protocol, three 500 ns-long production simulations were carried out at 310 K and 1 bar.

## Supplementary Material





## Data Availability

The Martini Lipid Benchmark, lipid parameters, and central analysis scripts are deposited as part of the Supporting Material of this work and on GitHub: https://github.com/Martini-Force-Field-Initiative/M3-Lipid-Parameters (commit identifier: 6d847335ac7f11fcd912c30ef3d23cbf5525d4fb). Additionally, the lipid parameters are also available in the new version of the Martini Database^109^ (MAD - https://mad.ens-lyon.fr/explore). Supporting results and methods are provided in the Supporting Information.

## Notes

Supplementary methods and simulation details; additional figures and tables including lipid bilayer properties, order parameters, diffusion constants, Ca^2+^ density profiles, protein–lipid binding, and ternary phase behavior; glossary of lipid abbreviations; extended benchmarking results (PDF)
Supporting materials and data; the Martini Lipid Benchmark, lipid parameters, and central analysis scripts (ZIP)
