# Supplementary material for: The Martini 3 Lipidome: Expanded and Refined Parameters Improve Lipid Phase Behavior
Source: ACS Cent Sci. 2025 Jul 31;11(9):1598–610. doi: 10.1021/acscentsci.5c00755 (PMC12464760; doi:10.1021/acscentsci.5c00755)
Supplement: Supplementary file 1 [file oc5c00755_si_001.pdf]

Supplementary Information:

The Martini 3 Lipidome: Expanded and Refined Parameters Improve Lipid Phase Behavior

Kasper B. Pedersen(1), Helgi I. Ingólfsson(2)†, Daniel P. Ramirez-Echemendia(3)†, Luís Borges-Araújo (4,5)†, Mikkel D. Andreasen(1), Charly Empereur-mot(9), Josef Melcr(8), Tugba N. Ozturk(2), W. F. Drew Bennett(2), Lisbeth R. Kjølbye(11), Christopher Brasnett(8), Valentina Corradi(3), Hanif M. Khan(3), Elio A. Cino(3), Jackson Crowley(12), Hyuntae Kim(14), Balázs Fábián(14), Ana C. Borges-Araújo(15), Giovanni M. Pavan(10), Guillaume Launay(4,5), Fabio Lolicato(6,7), Tsjerk A. Wassenaar(8), Manuel N. Melo(15), Sebastian Thallmair(16), Timothy S. Carpenter(2), Luca Monticelli(12, 13), D. Peter Tieleman(3), Birgit Schiøtt(1), Paulo C. T. Souza (4,5)**, and Siewert J. Marrink(8)*

(1) Department of Chemistry, Aarhus University, Langelandsgade 140, 8000 Aarhus C, Denmark
(2) Physical and Life Sciences (PLS) Directorate, Lawrence Livermore National Laboratory, Livermore, CA 94550, USA
(3) Centre for Molecular Simulation and Department of Biological Sciences, University of Calgary, 2500 University Dr. NW, Calgary, AB, Canada T2N 1N4
(4) Laboratoire de Biologie et Modélisation de la Cellule, CNRS, UMR 5239, Inserm, U1293, Université Claude Bernard Lyon 1, Ecole Normale Supérieure de Lyon, 46 Allée d’Italie, 69364, Lyon, France.
(5) Centre Blaise Pascal de Simulation et de Modélisation Numérique, Ecole Normale Supérieure de Lyon, 46 Allée d’Italie, 69364, Lyon, France.
(6) Heidelberg University Biochemistry Center, Heidelberg, Germany
(7) Department of Physics, University of Helsinki, Helsinki, Finland
(8) Groningen Biomolecular and Biotechnology Institute, Nijenborgh 7, 9747 AG Groningen, The Netherlands
(9) Department of Innovative Technologies, University of Applied Sciences and

Arts of Southern Switzerland, Polo Universitario Lugano, Campus Est, Via la Santa 1, 6962 Lugano-Viganello, Switzerland

(10) Politecnico di Torino, Department of Applied Science and Technology, Corso Duca degli Abruzzi 24, 10129 Torino, Italy

(11) Pharmaceutical Sciences, AstraZeneca R&D Gothenburg, Mölndal, 431 83, Sweden

(12) Molecular Microbiology and Structural Biochemistry (MMSB), UMR 5086 CNRS & Univ. Lyon, France

(13) Institut National de la Santé et de la Recherche Médicale (INSERM), France

(14) Department of Theoretical Biophysics, Max Planck Institute of Biophysics, Max-von-Laue Straße 3, 60438 Frankfurt am Main, Germany

(15) Instituto de Tecnologia Química e Biológica António Xavier, Universidade Nova de Lisboa, Av. da República, 2780-157 Oeiras, Portugal

(16) Frankfurt Institute for Advanced Studies, Ruth-Moufang-Str. 1, 60438 Frankfurt am Main, Germany

Corresponding authors:

*Siewert-Jan Marrink, email: s.j.marrink@rug.nl

**Paulo C. T. Souza, email: paulo.telles_de_souza@ens-lyon.fr

†These authors contributed equally.

Table of Contents

Lipid Glossary 4

Supplementary Results A) Bilayer Bending Moduli 4

Supplementary Results B) Automatically Generated Lipid Topologies Allows for Extensive Exploration of Bilayer Properties 7

Supplementary Results C) Density and Interfacial Tension of Di- and Triolein Neutral Lipids 11

Supplementary Results D) Additional Comments on Phosphatidylserine Phase Separation Modulated by Ionic Concentration 12

Supplementary Results E) Simulations of Non-lamellar Lipid Phases Have Potential in Drug Delivery and Food Applications 14

Supplementary Results F) The Martini 3 Lipidome Enables Simulation of Bilayers with Realistic Lipid Composition 19

Supplementary Results G) Modeling of Multi-Component Membranes Enables Studies of Protein-lipid Interactions 21

Supplementary Discussion H) Limitations of the Martini Model and Future Directions 34

Supplementary Methods I) Reference Simulations 37

Supplementary Methods J) Evaluation of Benchmarks 39

Supplementary Methods K) Construction of Complex Lipid Bilayers in Martini 3 44

Supplementary Methods L) Protein-lipid Interactions 45

Supplementary Methods M) Quantifying Bilayer Phase Behavior 49

Supplementary Data N) Martini Database and API 54

References 55

Lipid Glossary

PC: Phosphocholine
PE: Phosphoethanolamine
PG: Phosphoglycerol
PS: Phosphoserine
PA: Phosphatidic Acid
TAP: Trimethylammonium-propane
CL: Cardiolipin
BMP: Bis(monoacylglycerol)phosphate
POPC: 1-palmitoyl-2-oleoyl-*sn*-glycero-3-phosphocholine
PDPC: 1-palmitoyl-2-docosahexaenoyl-*sn*-glycero-3-phosphocholine
PLC18: 1-octadecenyl-2-oleoyl-*sn*-glycero-3-phosphocholine
POPCE: 1-O-hexadecyl-2-O-(9Z-octadecenyl)-sn-glycero-3-phosphocholinePSM: N-palmitoyl-D-*erythro*-sphingosylphosphorylcholine

Supplementary Results A) Bilayer Bending Moduli

Lipid bilayer bending and remodeling are extremely important phenomena in vivo. Simulations of vesicles and even whole-cell-scale systems have previously been investigated using the Martini force field[1-4]. The underlying biophysics of bilayer bending is therefore important to model and reproduce. We therefore estimated bilayer bending moduli (k_c) for a selection of lipids that have previously been studied by Doktorova et al. using atomistic CHARMM36 simulations[5]. Figure S1,A shows the correlation between k_c values obtained from lipid tail splay using the Real-Space Fluctuations (RSF) method[5], comparing bilayer simulations using Martini 2, Martini 3, and CHARMM36. The RSF results obtained using the reparametrized Martini 3 lipids show an improved correlation with CHARMM36 data compared to Martini 2. We also observe a general improvement in average P_2 bond order parameters compared to atomistic results (Figure S1,B), in agreement with the improved lipid tail splay quantified by the RSF method. APL values also show a better correlation with atomistic data compared to Martini 2, although they are systematically lower (Figure S2), as previously discussed.

We also estimated bending moduli (k_c) using an alternative method using bilayer buckling[6-8]. The buckling results correlate with our RSF results, however trending towards higher estimated values (Table S1).

While the k_c trends reported here are also consistent with experimentally obtained bending moduli of the lipids[5], we focused on a comparison with atomistic reference data because experimental values show considerable variation depending on the experimental technique used[5, 9, 10], as previously noticed[11]; in addition, other factors such as temperature and salt concentration[12] make a direct comparison problematic.

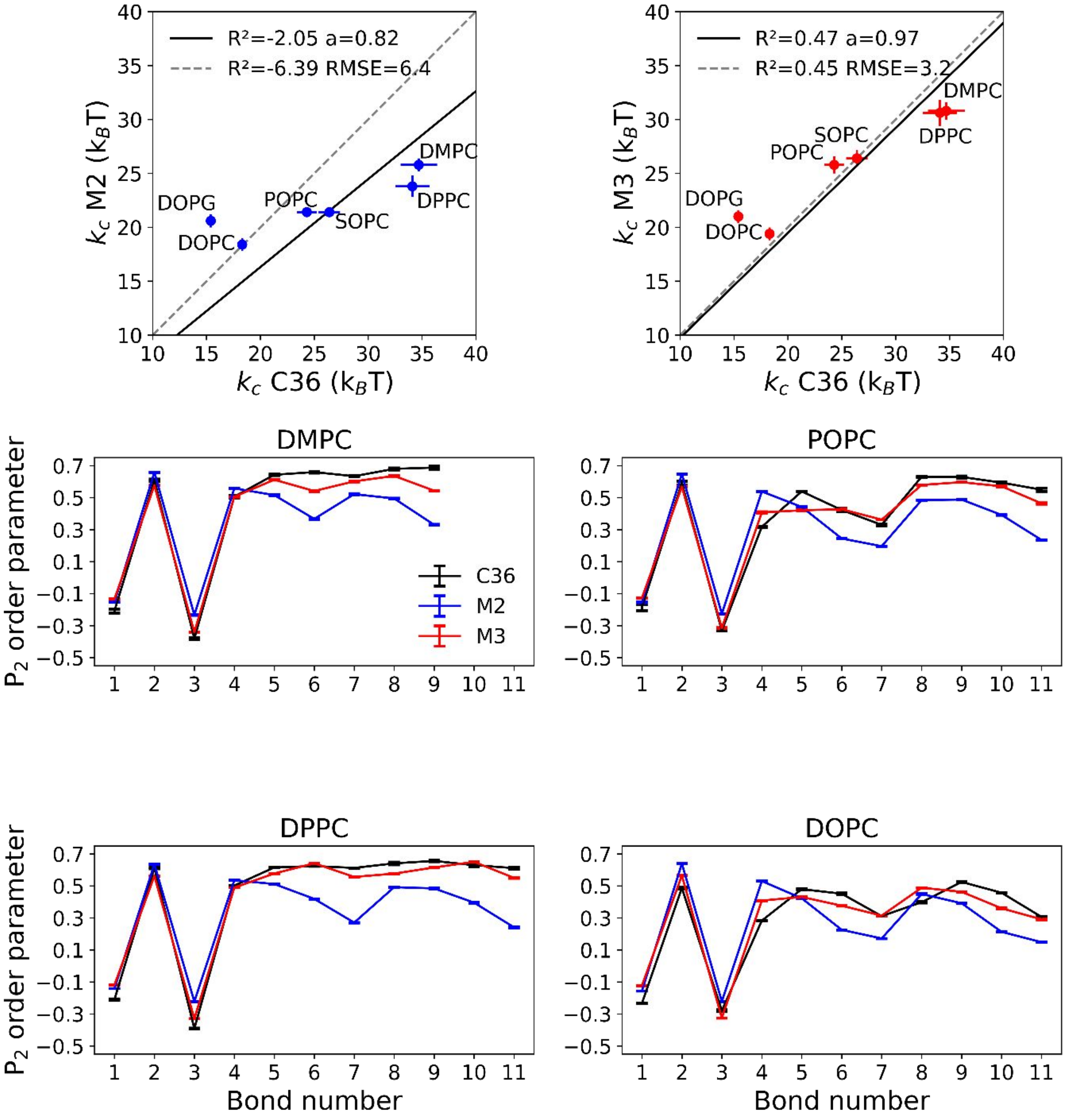

Figure S1. Reparametrized Martini 3 lipids improve bilayer bending modulus and lipid tail order. *(A) Comparison of bilayer bending moduli (k_c) values calculated by the RSF method from M2 (blue, left)*

and M3 (red, right) compared to CHARMM36 results. (B) Average lipid P_2 order parameters of M2 (blue) and M3 (red) lipid bilayers, compared to equivalent forward-mapped CHARMM36 simulations (black).

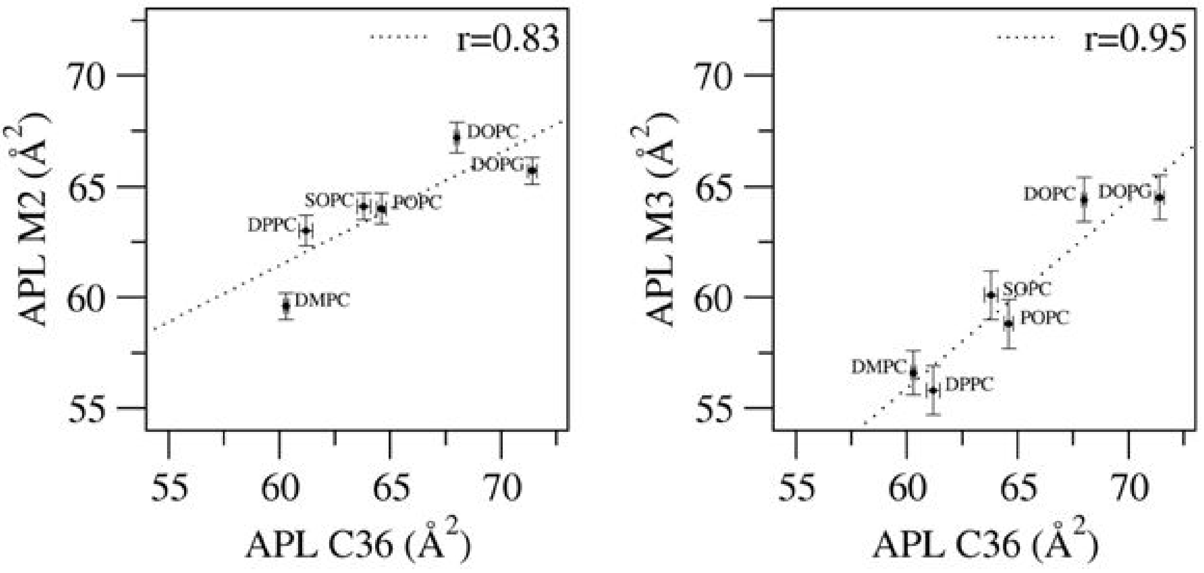

Figure S2. *Area per lipid of DMPC, DPPC, POPC, SOPC, DOPC, and DOPG lipid bilayers simulated using CHARMM36[5], Martini 2 and Martini 3.*

Table S1. *k_c (k_BT) values estimated by RSF and buckling. *SOPC and POPC have the same topology in Martini 2.*

	k_c RSF M3	k_c Buckling M3	k_c RSF M2	k_c Buckling M2	k_c RSF CHARMM36[5]
POPC (25ºC)	25.8±0.8	29.2±0.2	21.4±0.4	32.3±0.3	24.3±0.9
DPPC (50ºC)	30.6±1.2	26.9±0.3	23.8±1.0	31.6±0.3	34.1±1.6
DOPC (25ºC)	19.4±0.6	24.4±0.1	18.4±0.6	27.7±0.4	18.3±0.4
SOPC (25ºC)	26.4±0.8	28.8±0.4	*	*	26.4±1.0
DMPC (30ºC)	30.8±0.8	24.6±0.2	25.8±0.6	28.6±0.5	34.7±1.7
DOPG (25ºC)	21.0±0.6	24.9±0.2	20.6±0.6	21.3±0.2	15.4±0.4

Supplementary Results B) Automatically Generated Lipid Topologies Allows for Extensive Exploration of Bilayer Properties

To facilitate exploring a wide range of lipid types, a lipid topology generator was constructed using the refined Martini 3 lipidome parameters. Here, a set of 200 phospholipids was generated, their bilayer properties explored, and a comparison was made to Martini 2 lipids where parameters were available. We explored 5 different headgroup types: phosphatidylcholines (PC), phosphatidylethanolamine (PE), phosphoglycerol (PG), phosphatidylserine (PS), and phosphatidic acid (PA); each combined with 40 different acyl chain combinations of varying tail length and saturation. SI Table S2 shows the nomenclature used and examples of corresponding acyl chain names. Figure S3 contains eight calculated membrane properties, namely PO4-PO4, DHH, DB, and 2Dc bilayer thickness values as well as area per lipid, lipid diffusion, area compressibility (K_a), and P_2 order parameters. Additionally, the same lipids were explored by mixing them in at 20 mol% in a POPC, DLPE, or SSM-CHOL base, Figure S4 shows the APL for each lipid and lipid mixture, and all membrane properties for all systems are listed in SI Table S3.

Martini 2 captures membrane properties quite accurately[13-15], and the trends captured by both the reparametrized Martini 3 lipid parameters and Martini 2 are overall similar (Figure S1). However, Martini 3 results differ from Martini 2 in a few key areas. The APL of the reparametrized Martini 3 lipids is slightly lower for all tested lipids, a compromise made to improve known issues of too low gel-liquid phase transition temperatures of Martini 2[15]. In the reparametrized Martini 3 lipids, the APL, diffusion, and area compressibility (K_a) (Figure S3) properties show a clear distinction between liquid and gel-phase lipids. For example, saturated Martini 3 lipids transition from liquid to gel between di-myristoyl (14:0/14:0) and di-palmitoyl (16:0/16:0) lipid tails at the tested temperature of 310 K but Martini 2 lipids remain liquid, even at longer tail lengths. Another key difference is the higher granularity in resolving lipid tails, incrementing at 2 instead of 4 carbons and resulting in a more gradual change in lipid properties compared to Martini 2.

This automated study resulted in over 1300 different simulation systems and all analyzed bilayer properties are available in tabulated format in Table S3. The results

are valuable for comparison between lipids, to explore overall trends in membrane properties, and as a reference for expected properties of the tested lipids. However, as these are spatially small simulations, with specific temperature and pressure controls, performed at a specific lipid charge state, and all started in a semi-liquid phase, several caveats must be considered. For example, starting with small, preformed bilayers (stabilized by periodic boundary conditions) allows for simulations of planar membranes even with lipids that would normally not form lamellar phases. Additionally, the reported values are not corrected for finite size effects[16], and lipid phase transition can vary based on the initial state.

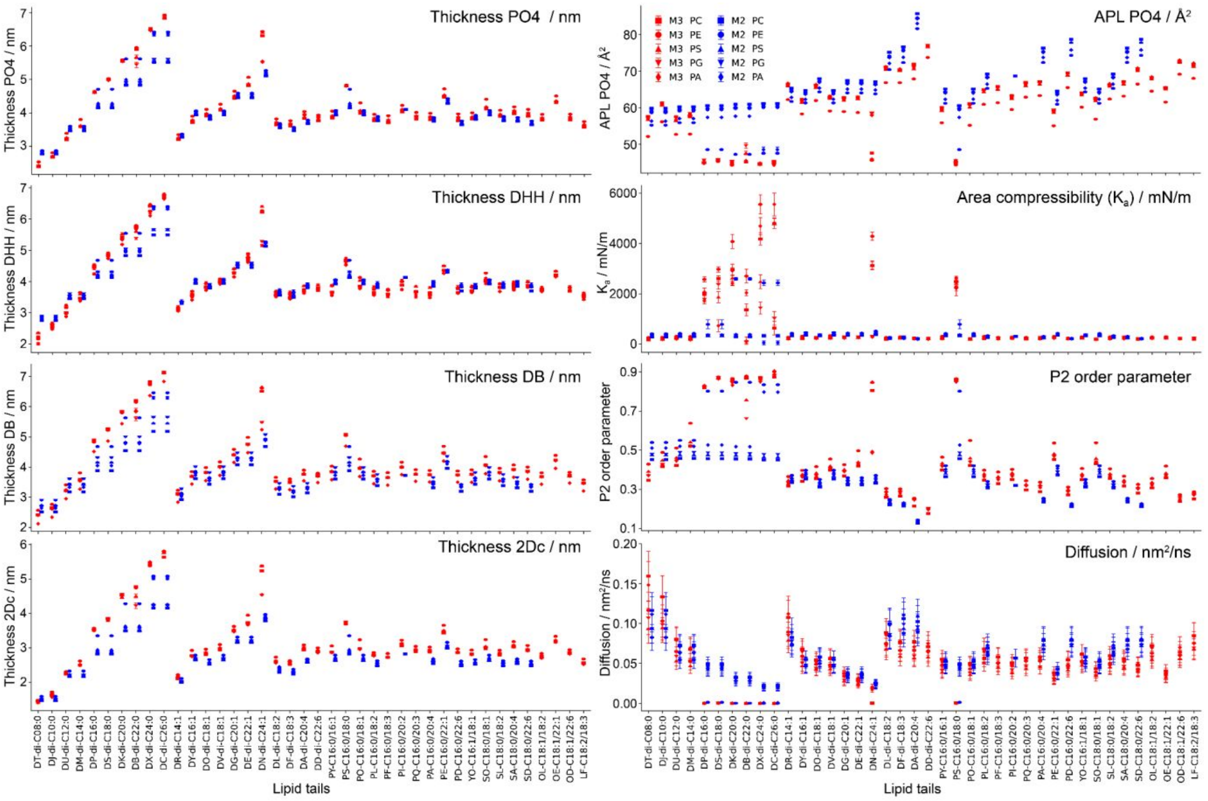

Figure S3. *Lipid properties of common diacylglycerol lipids. All calculated lipid properties of common diacylglycerol lipids. PO4, DHH, DB and 2Dc bilayer thickness values as well as area per lipid (APL), lipid diffusion, area compressibility (K_a) and P2 order parameters are shown for 40 different tail acyl chains combined with five different headgroup types all are simulated with Martini 3 as well as Martini 2 comparisons where parameters were available. At the simulation temperature of 310 K, as expected, several of the longer saturated-tailed lipids are in the gel phase in Martini 3 while only some of these lipids are in the gel phase in Martini 2. Note that Martini 2 does not differentiate between tails that differ by 2 carbons, we therefore put the same Martini 2 value for both DPPC (16:0/16:0) and DSPC (18:0/18:0). All values are provided in Table S3.*

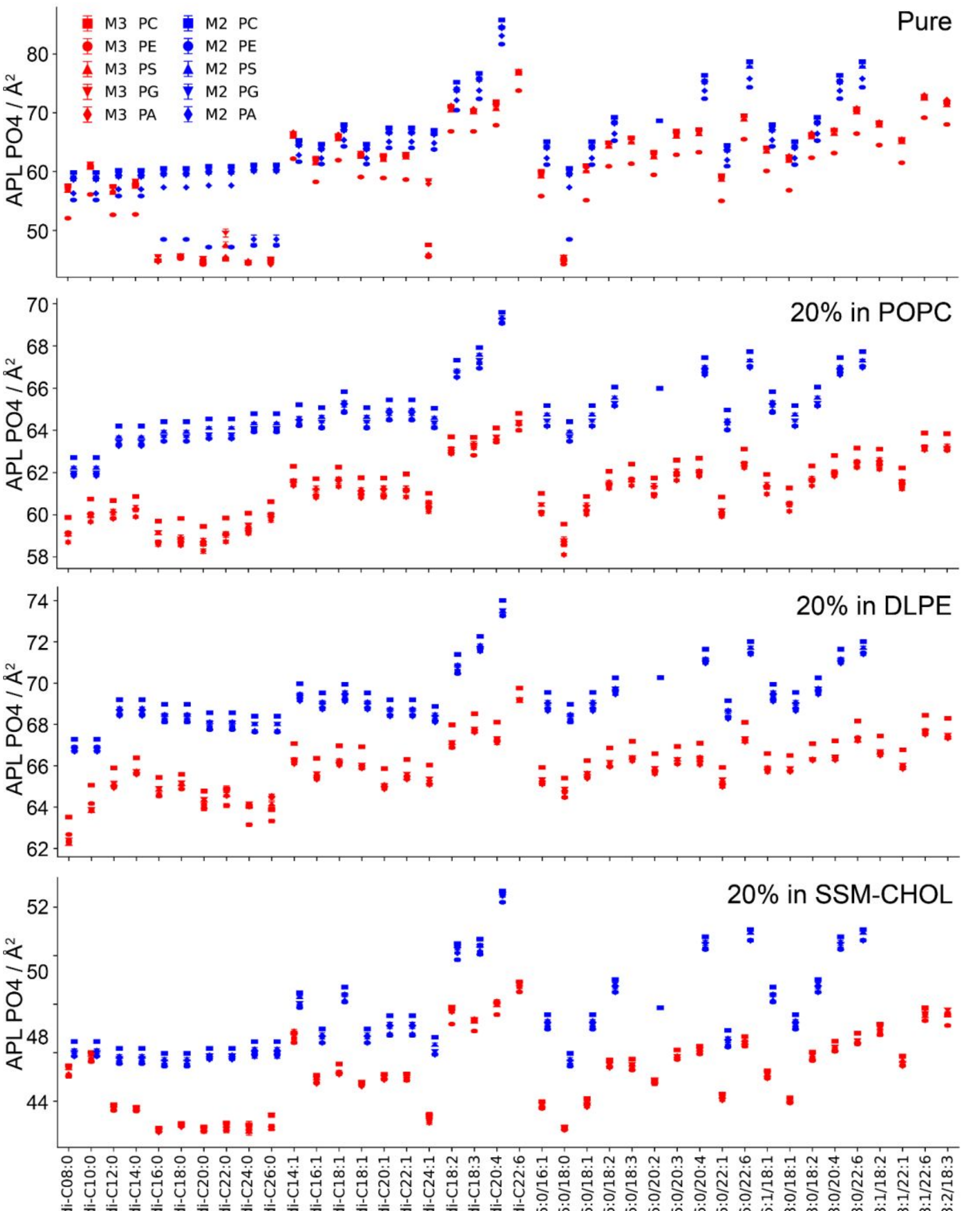

Figure S4. *Lipid properties of mixed lipid systems. In addition to the pure simulations of the 200 common diacylglycerol lipids shown in Figure 4 and S1 the same lipids were simulated at 20 mol% in a base of POPC, DLPE or SSM-CHOL to assess their relative changes to membrane properties. Here APL is shown for the pure as well at the three different base lipid mixtures. APL as well as other calculated lipid properties are provided in Table S3.*

Table S2. *Nomenclature for Martini 3 lipid fatty acid tails. [a]Note D can also stand for Di such as in DOPC for 1,2-Dioleoyl-sn-glycero-3-phosphocholine. [b]CG beads represent two to four carbon atoms (small and big letters respectively); the carbon chain is saturated for C beads, has 1 double bond in D beads, and ~1.5 in F beads. [c]Due to the CG mapping the underlying atomistic lipids are not unique, a few examples are given in the table.*

One letter name	Bead assignment[b]	Corresponding atomistic tails	Examples of corresponding fatty acid names[c]
T	cC	C8:0	C08:0 octanoyl
J	CC	C10:0	C10:0 decanoyl
U	cCC	C12:0	C12:0 lauroyl
M	CCC	C14:0	C14:0 myristoyl
P	cCCC	C16:0	C16:0 palmitoyl
S	CCCC	C18:0	C18:0 stearoyl
K	cCCCC	C20:0	C20:0 arachidoyl
B	CCCCC	C22:0	C22:0 behenoyl
X	cCCCCC	C24:0	C24:0 lignoceroyl
C	CCCCCC	C26:0	C26:0 hexacosanoyl
R	CDC	C14:1	C14:1(9c) myristoleoyl
Y	cCDC	C16:1	C16:1(9c) palmitoleoyl
O	CDCC	C18:1	C18:1(9c) oleoyl
G	cCDCC	C20:1	C20:1(11c) eicosenoyl (11-eicosenoic acid) or gondoic acid
E	CCDCC	C22:1	C22:1(11c) or C22:1(13c) erucoyl
N	cCCDCC	C24:1	C24:1(15c) nervonic acid
V	CCDC	C18:1	C18:1(11c) cis-vaccenic acid
L	CDDC	C18:2	C18:2(9c,12c) linoleoyl
F	CDDD	C18:3	C18:3(9c,12c,15c) alpha-linolenic acid
I	cCDDC	C20:2	C20:2(11c,14c) eicosadienoyl
Q	cDDDC	C20:3	C20:3(8c,11c,14c) eicosatrienoyl or dihomo-gamma-linolenic acid
A	cFFDC	C20:4	C20:4(5c,8c,11c,14c) arachidonoyl
D[a]	DFFDD	C22:6	C22:6(4c,7c,10c,13c,16c,19c) docosahexaenoic acid

Table S3. *Bilayer properties for common diacylglycerol lipids. Analysis of bilayer thickness (PO4 to PO4, DHH, DB, and 2Dc), area per lipid (APL), area compressibility (K_a), average P_2 lipid tail order parameter and lipid diffusion is provided for 238 different Martini 3 lipids, by themselves or mixed in 20 mol% in a base of POPC, DLPE or SSM-Cholesterol, as well as corresponding Martini 2 lipids when available.* This table is provided on GitHub (https://github.com/Martini-Force-Field-Initiative/M3-Lipid-Parameters).

Table S4. *Data plotted in Figure 1, D-G. Experimental and simulated area per lipid (APL) and bilayer thickness (DHH, DB, and 2Dc), This table is provided on GitHub (https://github.com/Martini-Force-Field-Initiative/M3-Lipid-Parameters/tree/main/Martini_lipid_Benchmark).*

Supplementary Results C) Density and Interfacial Tension of Di- and Triolein Neutral Lipids

To validate the lipid parameters in reproducing properties of neutral lipids, we simulated DOG (1-2-dioleoyl-*sn*-glycerol) and TOG (1,2,3-tri-oleoyl-glycerol) both in the bulk (NPT ensemble) and in a water interface system (NP*z*T ensemble). This allowed the calculation of density from the simulation box vectors and interfacial tension (IT) with water using the Irving-Kirkwood method[17] implemented in the GROMACS 2024.0 energy tool (Table S4).

Table S4. *Di- and triglyceride density and interfacial tension. *We were unable to find a citable source for diolein density, but the "ChemicalBook" webpage (https://www.chemicalbook.com/ChemicalProductProperty_EN_CB5179215.htm) lists it as 0.917 g/cm³.*

Step	Experimental density (g/cm³)[18]	Simulated density (g/cm³)	Experimental interfacial tension (mN/m)[18]	Simulated interfacial tension (mN/m)
Diolein	*	0.928 [0.001]	17.2 [1.4]	18.8 [0.1]
Triolein	0.909 [0.018]	0.914 [0.001]	30.6 [2.4]	29.9 [0.1]

Supplementary Results D) Additional Comments on Phosphatidylserine Phase Separation Modulated by Ionic Concentration

Phosphatidylserine (PS), the most abundant negatively charged lipid in mammalian cell membranes, has been a focal point in studies of cation-lipid interactions. Research has shown that Ca^{2+} and/or Mg^{2+} can promote vesicle fusion[19], induce aggregation and phase separation[20, 21] or even phase transitions[22] in PS-containing membranes. While PS has been extensively studied, similar behaviors have been noted in other anionic lipids, such as phosphoinositides[19, 23, 24], phosphatidylglycerol[25-27], and phosphatidic acid[20, 28]. We tested the capabilities of the new Martini 3 lipid models to successfully reproduce some of these cation-induced phenomena, focusing on the Ca^{2+}-induced aggregation and resulting phase change behavior of PS.

We started by examining the localization of Na^{+} and Ca^{2+} ions around the PS headgroup, which has been relatively well characterized. Both experimental data and AA MD simulations indicate that binding of Ca^{2+} ions to PS occurs primarily in the phosphodiester region[29, 30]. Consequently, this binding leads to dehydration of the region and displaces monovalent ions, such as K^{+}, Na^{+}, and Li^{+}, to less deeply buried locations[22, 31, 32]. Our Martini 3 simulations correctly captured this behavior, as seen by the density profiles derived from our simulations of POPS bilayers, in the presence and absence of Ca^{2+}.

Next, we aimed to assess whether our model accurately captures Ca^{2+}-induced PS phase change. Experimental evidence indicates that at room temperature and in the absence of Ca^{2+}, lipid bilayers composed of POPS exist in a liquid-crystalline phase (T_m~286 K)[22]. However, upon the addition of excess Ca^{2+}, POPS undergoes a phase transition from the liquid-crystalline phase to a gel phase (T_m~393 K)[22]. This Ca^{2+}-induced PS phase change is accurately captured by our model, as evidenced by the formation of the POPS gel phase in the presence of Ca^{2+} at 300 K. More extreme Ca^{2+}-to-PS ratios can lead to further phase transitions, namely the formation of cochleate phases[33, 34]. However, the formation of these complex macroscopic structures was beyond the scope of this test.

Ca^{2+} has also been shown to induce lateral-phase separation[21, 35] of PS in mixed PC:PS bilayers. To test this, we simulated lipid bilayers composed of 1:1 mixtures of POPC:POPS, in the presence and absence of Ca^{2+}. In the absence of Ca^{2+}, a single miscible fluid phase is observed. However, upon the addition of Ca^{2+}, POPS undergoes substantial lateral-phase separation from POPC, resulting in the formation of two distinct phases: a PS-rich phase and a PC-rich phase. This separation is visible in the bilayer snapshots and is further supported by the enrichment of POPS-POPS first neighbors and the simultaneous depletion of POPS-POPC first neighbors.

Overall, these findings demonstrate that the new Martini 3 lipid models can reproduce experimental Ca^{2+}-induced PS behavior. This behavior could not be reproduced with Martini 2, which accurately localized Na^{+} and Ca^{2+} ions around the PS headgroup but failed to replicate Ca^{2+}-induced phase changes and Ca^{2+}-induced phase separation (Figure S5). Accurately representing the interaction of anionic lipids with divalent cations is essential for modeling the complex nature of biological membranes.

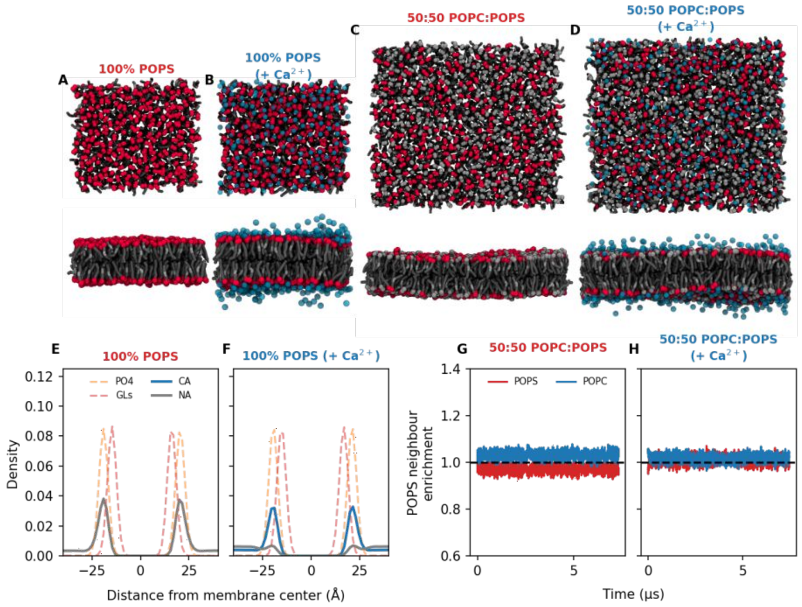

Figure S5. *Modeling the impact of Ca^{2+} on phosphatidylserine-containing bilayers using Martini 2. Bilayer snapshots of 12 nm^2 100% POPS bilayers, in the absence (A) and presence (B) of Ca^{2+}. Bilayer snapshots of 20 nm^2 POPS:POPC 50:50 bilayers, in the absence (A) and presence (B) of Ca^{2+}. A Ca^{2+}-to-PS ratio of 1.5 was used. Shown snapshots were taken from the end of each simulation (10 us). POPS and POPC GL* beads are shown in red and grey, respectively. Acyl-chains are colored in grey.*

Ca^{2+} is shown as translucent blue. The density profile of ions (Na^{+}, Ca^{2+}), phosphate (PO4) and glycerol linker (GLs) obtained from simulations of 12 nm^2 100% POPS bilayers, in the absence (E) and presence (F) of Ca^{2+}. All bilayers are centered around its hydrophobic center. Relative enrichment/depletion of POPS neighboring lipids (within 1.2 nm) obtained from simulations of 20 nm^2 POPS:POPC 50:50 bilayers, in the absence (G) and presence (H) of Ca^{2+}. POPC and POPS neighbor enrichment/depletion is shown in blue and red, respectively.

Supplementary Results E) Simulations of Non-lamellar Lipid Phases Have Potential in Drug Delivery and Food Applications

Lipids exhibit a diverse range of structural phases, including lamellar bilayers, and non-lamellar geometries like hexagonal- and cubic phases, depending on environmental factors such as temperature, hydration, and lipid composition[36, 37]. These non-lamellar phases, particularly the hexagonal and cubic arrangements, are characterized by their highly organized internal structures, which result in distinct physical and chemical properties[37]. The ability of lipids to transition between these phases is crucial for understanding their behavior in biological systems, such as membrane fusion[38-40]. Non-lamellar lipid phases are important in industrial applications due to their unique structural and functional properties[41]. They can be employed to create stable emulsions and encapsulate bioactive compounds while offering improved stability, with applications in drug delivery and vaccines[42, 43], functional food products[44, 45], and cosmetics[46, 47]. Specifically, the application of inverse hexagonal and cubic lipid phases has shown great potential for encapsulation of drugs in e.g. lipid nanoparticles (LNPs)[42]. Figure S6,D shows an example of an LNP with the ionizable lipid MC3, built and simulated using the recent parameters from Kjølbye et al[48]. LNPs can be tailored to accommodate specific drugs, making them excellent candidates for advanced drug delivery systems[42, 49-52]. The ability of lipid models to replicate the inverse hexagonal phase under appropriate conditions is a crucial aspect of these models. Specifically, phosphatidylethanolamine (PE) lipids, which are well-characterized in the literature, can form the inverse hexagonal phase at specific temperatures, pressures, and hydration levels[53-56]. The same is the case for monoolein (MO), a well-characterized and commonly used lipid for creating cubic phase systems[57, 58]. MO, and other monoacylglycerides, can form cubic phases

spontaneously at room temperature and pressure[59]. Here, we validate our refined Martini 3 lipid parameters in producing the inverse hexagonal phase of PE and cubic phases formed by MO under various conditions.

The formation of the hexagonal phase of PE lipids was tested using two approaches. First, the transition from lamellar to inverse hexagonal phase was simulated, secondly, the inverse hexagonal phase was pre-build and tested for stability, measuring the lattice distances to compare to experimental data. The chosen PE lipid for the tests is DOPE which is experimentally well-characterized[53, 54]. The phase diagram of DOPE ranging from 273 K to 319 K, and hydration levels from ~ 2 to 20 water molecules per lipid, displays a small region where the lamellar phase is present, but otherwise, the inverse hexagonal phase is observed, or possibly co-existing with a cubic phase[53]. The lamellar phase can be observed at temperatures ranging ~273 to 295 K and with ~6 to 11 water molecules per lipid[53]. The transition from stacked DOPE bilayers (Figure S6,A) to the inverse hexagonal phase (Figure S6,B) was tested for temperatures at 273, 295, 303, and 320 K, and a hydration level of ~3 and 11 water molecules per lipid. The inverse hexagonal phase was observed to form across all the temperatures at low hydration (~3 water molecules per lipid), while for the higher hydration levels, the inverse hexagonal phase was only observed at 320 K, whereas at 273, 295, and 303 K the lamellar phase remained. This aligns with experimental results[53], except for at 303K, where the inverse hexagonal phase is suggested to form experimentally. Considering that there could be some metastability in the simulated transition from lamellar to the inverse hexagonal phase, we also pre-constructed inverse hexagonal phases and measured the lattice distance D_{hex} across different hydration levels (Figure S6,C). At 295 K, Martini 3 simulations results in smaller lattices distances compared to Martini 2, which is in better agreement with experimental data[53-55]. Increasing the temperature to 320K, results in a decrease in the lattice distance for 12 to 16 waters per lipid, which has also been observed experimentally[55, 60].

The formation of cubic phases was tested through the self-assembly of MO molecules in water at the experimental excess water point (40% weight water). The formation of a unit cell of the expected Q_{II}^{D} mesophase was confirmed through a surface fit of the

ideal surface[61, 62] to the terminal tail bead of the system (Figure S6,E). The stability of the cubic phase was further confirmed by self-assembly simulations at multiple temperatures and compositions (Figure S7). While cubic phase self-assembly was achieved in both Martini 2 and Martini 3 force fields, the unit cell sizes in Martini 3 were in general slightly smaller at similar compositions. Further, the phase transition from cubic to hexagonal at high temperatures was confirmed through temperature annealing simulations (Figure S6,F). The Q_{II}^{D}-H_{II} the transition was observed at around 347±4 K, slightly lower than the experimental transition at around 363 K, which again could be complicated by metastability. One challenge we faced was the simulation of multiple cubic unit cells. While multiple unit cell simulations have been performed previously on DOPE/fusion peptide systems using Martini[63], we could not simulate a stable array of 2x2x2 unit cells of the pure MO Q_{II}^{D} mesophases neither through self-assembly nor by artificially translating them before conducting dynamics, which may require more advanced system building and relaxation. For studies of the cubic phase as a host system for drugs and macromolecules, simulation of a single unit cell of the cubic phase with periodic boundaries could be sufficient.

The ability to simulate non-lamellar phases, such as hexagonal and cubic structures, using Martini 3 is significant for advancing research in novel therapies and opens opportunities for applications in drug delivery, food, and cosmetic industries, highlighting the potential of applying Martini 3 simulations across multiple fields.

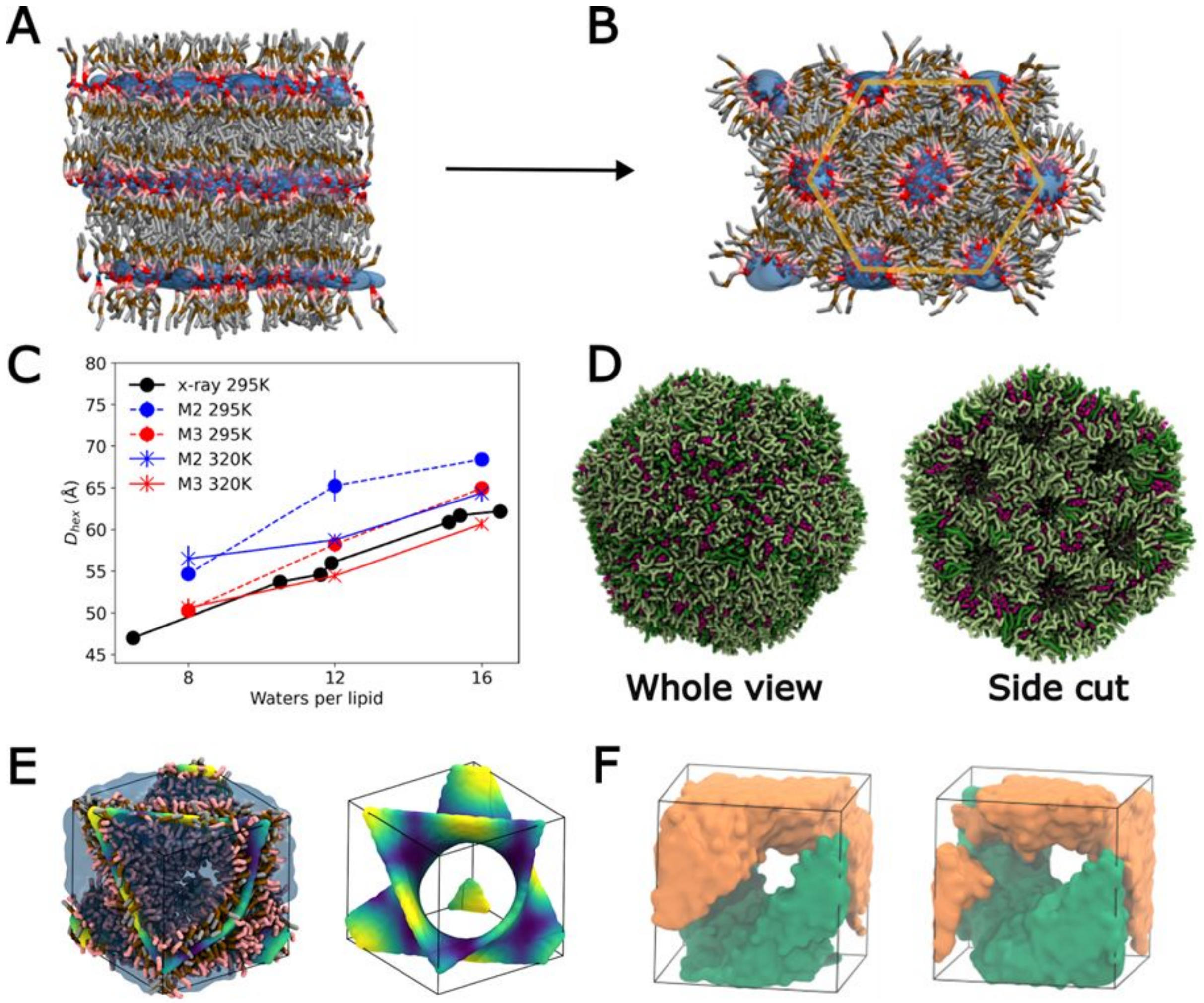

Figure S6. Hexagonal and cubic lipid phases. (*A) Snapshot of two stacked DOPE bilayers. (B) snapshot of the stacked DOPE bilayers changing into an inverse hexagonal phase in Martini 3 simulations. The water and ions are shown in light blue, the lipid headgroups in red, the linker in brown, and the tails in gray. (C) The lattice distance (D_{hex}) measured for Martini 2 and Martini 3 simulations of the pre-constructed inverse hexagonal phase. The experimental data is obtained from Rand and Fuller. (D) A snapshot of an MC3 LNP. The MC3 lipids are shown in green, cholesterol in magenta, and DSPC in dark green. No PEGylated lipids nor solvent or cargo are shown. (E) Simulation snapshot of a single cell of a MO Q_{II}^{D} phase, fitted with the underlying triply periodic minimal surface, and the surface on its own, coloured by Gaussian curvature. Regions of more negative curvature are in blue, and flatter regions are shown in yellow. (F) Water channels break during temperature annealing, marking the beginning of a phase transition from the Q_{II}^{D} to H_{II} phase.*

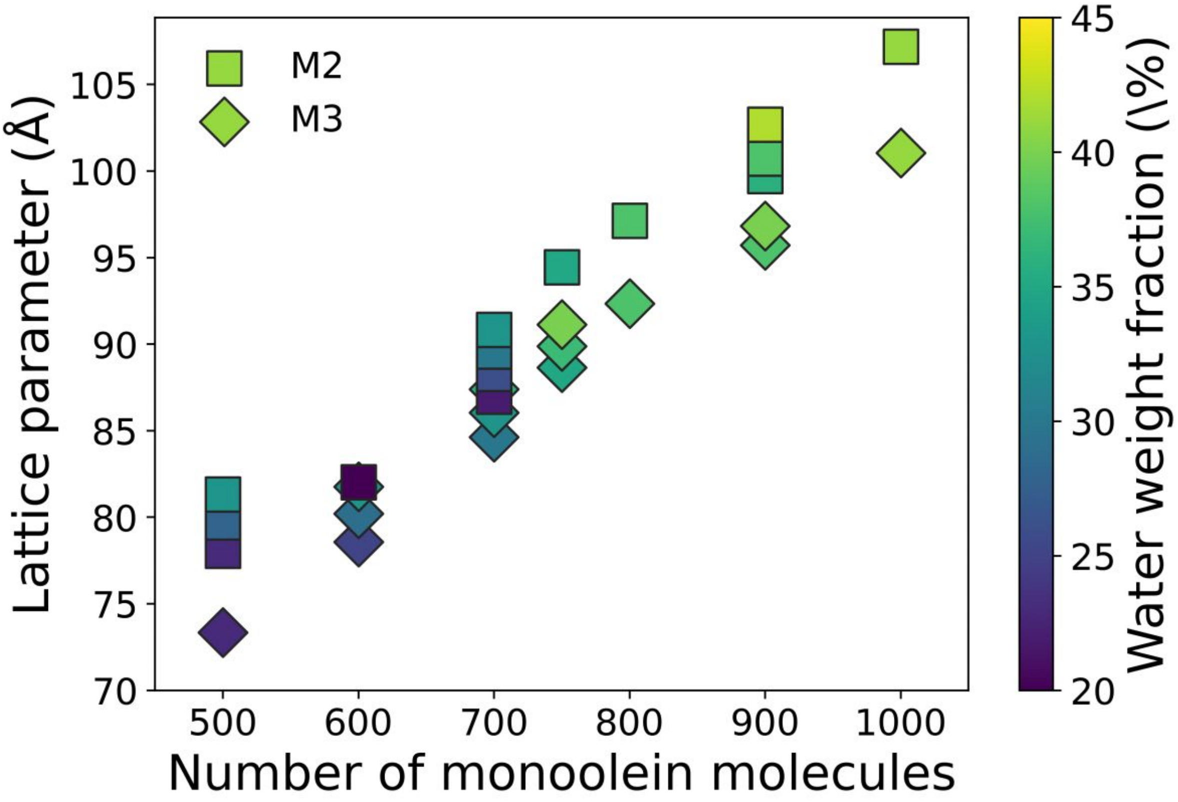

Figure S7. *Lattice parameters for QIID unit cells at varying water/lipid compositions, comparing Martini 2 and Martini 3 parameters.*

Supplementary Results F) The Martini 3 Lipidome Enables Simulation of Bilayers with Realistic Lipid Composition

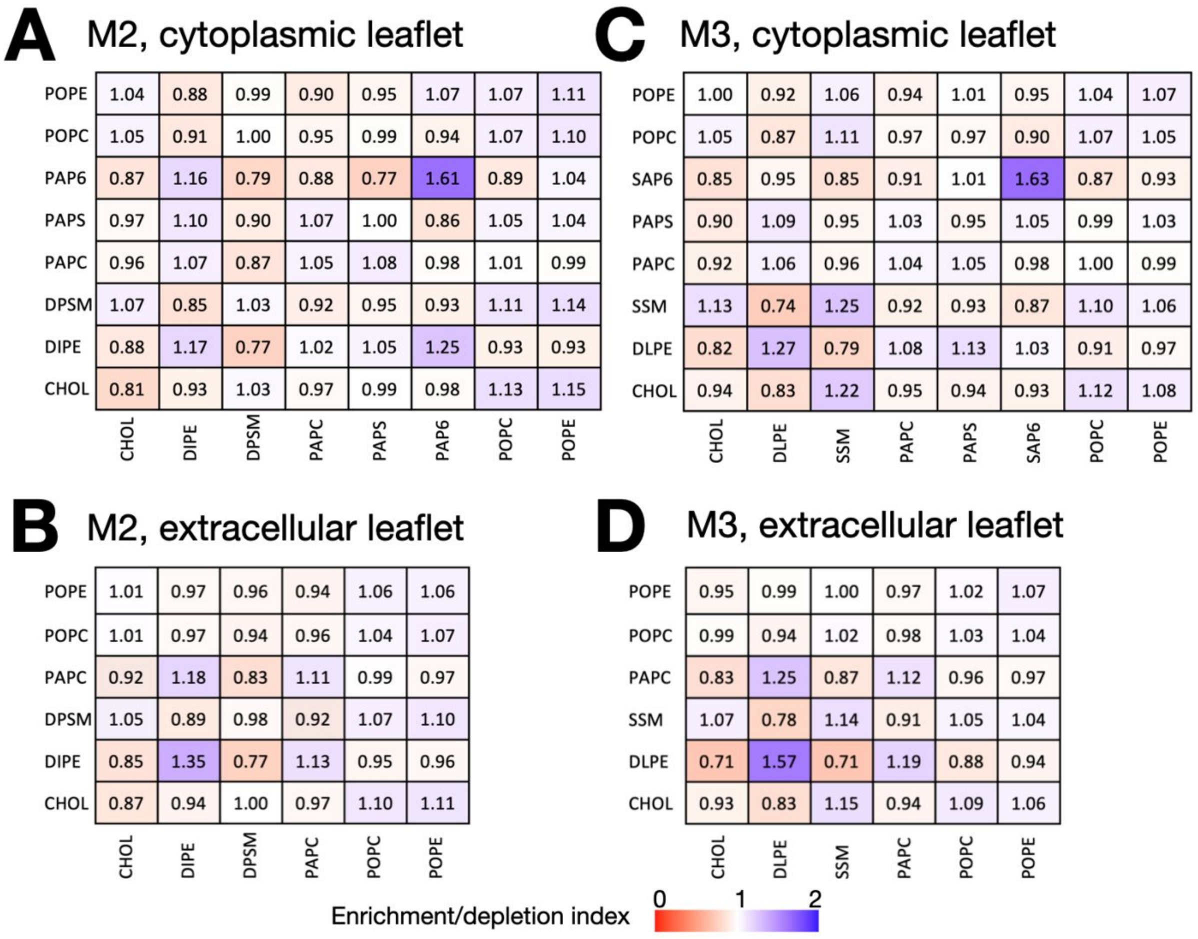

Figure S8. *Lipid enrichment/depletion indices for the cytoplasmic leaflet (A – Martini 2 (M2), C – Martini 3 (M3)) and extracellular leaflet (B – Martini 2, D – Martini 3) of the plasma membrane models. The indices are color-coded. The strongest red data point shows the most depleted lipid pairs whereas the strongest blue data point shows the most enriched lipid pairs. The indices were computed with, using a 10 μs-long trajectory for the Martini 2 model and a 40 μs-long trajectory for the Martini 3 model, LiPyphilic*[64] *Python package with a distance cut-off of 1.5 nm.*

Table S5. *Average membrane properties of the eight-lipid type mammalian plasma membrane model from Martini 2*[65] *and Martini 3.*

**Average properties**	**Martini 2**[65]	**Martini 3**
Bilayer thickness (nm)	3.94±0.02 (mean±SD)	4.22±0.01
Cholesterol flip-flop rate (s^{-1})	3.73±0.02 $\times 10^{6}$	5.86±0.02 $\times 10^{6}$
Diffusion coefficient (cm^2/s)	4.22±0.07 $\times 10^{-7}$	4.51±0.05 $\times 10^{-7}$

	Extracellular	*Cytoplasmic*	*Extracellular*	*Cytoplasmic*
Number of unsaturation per tail	0.72	1.30	0.72	1.30
Fraction of cholesterol	0.55	0.45	0.59	0.41
Position 3 order parameter	0.41±0.01	0.30±0.01	0.63±0.01	0.48±0.01
Area per lipid (nm^2)	0.515±0.001	0.556±0.001	0.466±0.001	0.527±0.002
Diffusion coefficient ($\times 10^{-7} cm^2/s$)	3.67±0.07	4.79±0.07	4.09±0.16	4.93±0.13

All standard deviation (SD) values were computed from the mean values from 4 equal parts of a 10 μs-long trajectory for the Martini 2 model and from 4 independent 20 μs-long trajectories for the Martini 3 model. The diffusion rate was computed only for phospholipids.

Table S6. *Lipid composition of the mammalian plasma membrane model adapted to the Martini 3 force field.*

Leaflet	CHOL	DLPE	SSM	PAPC	PAPS	SAP6	POPC	POPE
Extracellular	33.8%	5.9%	23.4%	11.6%	-	-	23.4%	1.9%
Cytoplasmic	25.3%	16.8%	11.2%	7.7%	16.8%	2.3%	14.4%	5.5%

Table S7. *Simulation protocol for the Martini 3 mammalian plasma membrane systems.*

Step	Time step	Total time	Thermostat	Barostat (semi-isotropic)
Minimization	-	1500 steps	-	-
Equilibration 1	1 fs	5 ps	Berendsen	Berendsen (τ_p = 10 ps)
Equilibration 2	5 fs	100 ps	Berendsen	Berendsen (τ_p = 3 ps)
Equilibration 3	10 fs	1000 ps	Berendsen	Berendsen (τ_p = 3 ps)
Equilibration 4	20 fs	1000 ps	v-rescale	Parrinello-Rahman (τ_p = 12 ps)
Production	20 fs	40 μs	v-rescale	Parrinello-Rahman (τ_p = 12 ps)

Supplementary Results G) Modeling of Multi-Component Membranes Enables Studies of Protein-lipid Interactions

The ability to model transmembrane proteins in a realistic lipid environment at extended timescales has been a hallmark of the Martini model for almost two decades[66-68]. Here, we tested the ability of the reparametrized Martini 3 lipids to identify known lipid-protein interactions in selected transmembrane and peripheral proteins.

During parameterization of the original Martini v3.0.0 lipid model, we found that the model could reproduce experimental PI(4,5)P_2 binding sites in the Kir2.2 protein (PDB ID 6M84)[69], a homotetrameric potassium channel activated by this lipid species (Figure S9,A)[70]. Here, we show that the results are reproducible with the reparametrized Martini 3 lipids. We performed a 30 µs-long simulation of Kir2.2 in a POPC bilayer with 5% PI(4,5)P_2 (called POP6 in the four-letter Martini 3 nomenclature) in the lower leaflet. Several POP6 lipids remain in close proximity to the protein for several microseconds (Figure S10,A), and lipid exchange can be observed at the four PI(4,5)P_2 sites (Figure S10,B). The fractional occupancy analysis shows that POP6 lipids occupy the four PI(4,5)P_2 headgroup sites in the tetramer, with occupancy maps confined around residues that are known to interact with PI(4,5)P_2 (Figures S9,A and S10,C).

We also simulated the bovine ADP/ATP carrier (PDB ID 1OKC)[71] to test interactions with cardiolipins bearing PO tails (namely, POCL) using Martini 3. The experimental structure shows three cardiolipins, (CDL800, CDL801, CDL802) which are bound in the lower leaflet. Occupancy maps of POCL, derived from two independent 30 µs-long simulations, show higher occupancy in the lower leaflet, without clearly defining individual sites (Figures S9,B and S11,A). At the chosen density isovalues, for POPE we retrieved no occupancy near the protein, while for POPC we identified confined regions in the upper leaflet in both simulations (Figures S9,B and S11,A). This is consistent with the results of a 30 µs-long Martini 2 simulation, where, however, POCL occupancy is more prominent in Martini 2 (Figure S11,B). The timescale of the

interactions between the protein and cardiolipins is also different between Martini 3 and Martini 2: The Martini 3 simulations show a much faster exchange of lipids in both the upper and the lower leaflet and near the POCL experimental sites (Figure S12,A,B), compared to Martini 2, wherein the lower leaflet a few POCLs remain within 5 Å from the protein for several µs (Figure S12,C), as previously shown[72] for tetra-oleoyl cardiolipins using Martini 2. Interestingly, bulk lipid diffusion is not significantly different between Martini 2 and Martini 3 (Figure S3), and the main difference in interaction patterns and timescales can be attributed to differences in the non-bonded interactions between the protein and lipid models in Martini 2 versus Martini 3. Martini 2 is known to be overly "sticky" for a range of applications[73, 74], and the faster exchange of lipids in Martini 3 may be realistic. However, further studies are needed to investigate exchange rates of lipids (residence times) and occupation of binding sites as compared with crystal structures, ideally compared to experimental measurements across many lipid types and protein systems. We are currently working on improvements in the protein model, which could help alleviate issues related to protein-lipid interactions.

Another useful application of Martini is the investigation of the binding mechanisms of peripheral membrane proteins. Here, we test several such protein-lipid systems including *Bt*PI-PLC[75-78], wild-type *Sa*PI-PLC[79, 80], PLC-d1 PH domain[81-83], FGF2[84-86] and Ebola virus VP40 matrix protein[87, 88].

For *Bt*PI-PLC, we performed binding simulations with pure DMPC or POPC bilayers. We calculated the minimum distance between the protein and membrane, with focus on two important membrane binding residues[75, 76, 78, 89], Lys44 and Tyr246. For the DMPC system, we ran five independent 1 µs-long simulations, where we did not observe stable binding at the known protein-lipid interface (Figure S13,A). Five more independent 5 µs-long simulations were carried out to test if the timescale of the simulations could impact binding: We retrieved a similar trend (Figure S13,B), with some simulations showing a rolling motion of the protein on the membrane surface. In contrast, simulations performed with POPC lipids did retrieve the binding of the protein to the membrane with the right orientation (Figure S9,C), with binding persisting over the timescale of the simulations (Figure S13,C). Even when binding to the membrane occurs through the wrong interface, the protein reorients and binds with the right

orientation (Figure S13,C). The different results obtained from the DMPC and POPC simulations highlight the tightness of the DMPC membrane, possibly linked to the general slight decrease in APL resulting from the new parameters. This highlights the impact of membrane composition on protein binding, and how it may affect membrane penetrating residues, proteins, or peptides.

The wild-type *Sa*PI-PLC was tested with both POPG and POPC membranes as positive and negative controls, respectively, as this protein binds to POPG and has no affinity for POPC membrane[77, 79, 90]. 1 μs-long simulations reproduce the expected behavior: Stable binding was retrieved in the presence of POPG lipids, while in the presence of POPC lipids bound states are short-lived, and binding/unbinding events are frequent (Figure S14).

Simulations of the PH domain were set up in the presence of two different bilayers: POPC:POPS:PIP_2:PIP_3 = 73:20:5:2 and POPC:PIP_2 = 95:5, and for each bilayer, we performed two 5 μs-long simulations. For the first bilayer, which carries 27% of negatively charged lipids, the PH domain binds to the bilayer (Figures S9,D and Figure S15,A) in the right orientation, through Lys30 and Arg40, which are known PIP binding residues[70]. However, with the less negatively charged bilayer, which includes only 5% PIP_2 lipids, the protein does not form a stable complex with the membrane even for 1 μs timescale (Figures S15,B and S16).

We also tested the interactions between the Fibroblast growth factor II (FGF2)[91-93], a PI(4,5)P_2 binding protein[93-96], and a membrane with 5 mol% of PI(4,5)P_2 in a POPC background. Like the above observations for the PH domain, FGF2 does not form a stable complex with the membrane over the 10 μs simulation (Figure S17).

Finally, we tested the viral protein 40 (VP40) dimer and PS lipid interactions in a membrane composed of 30 mol% POPS, and 30 mol% cholesterol in a POPC background. In previous CHARMM36m simulations, we observed and quantified the binding of the VP40 dimer with the membrane and successfully reproduced the interactions with experimentally known binding pockets, including K224, K225, K274, and K275[97]. Here, in Martini 3 simulations, while binding to the membrane was not an issue, achieving the correct orientation of the protein with the experimental binding residues facing the membrane was challenging. The choice of the initial system from which the protein was coarse-grained made a significant difference for the VP40

dimer. The best results were achieved by coarse-graining the protein from an atomistic model that was pre-bound to the membrane. In this configuration, the protein interacted with the membrane via the experimentally identified binding residues in ~90% of the frames where membrane interaction occurred (Figure S9E, right panel).

Taken together, we have demonstrated that our reparametrized lipids together with the Martini 3 protein model can be used to study a broad range of protein-membrane systems at long-microsecond timescales, needed to effectively investigate protein-lipid interactions. However there is still room for improvement in the reproduction of certain protein-lipid systems, e.g. stable binding of peripheral proteins to known lipid partners, which we expect will require not only improvements in the lipid parameters but also changes to the current protein model[98, 99]. With the number of available Martini 3 lipid topologies now in the thousands, together with the natural variation of protein sequences, the possibilities of constructing and studying proteins in *ad-hoc* membrane environments are limitless. We therefore rely heavily on community feedback, both positive and which areas we should focus on improving, ideally backed by experimental results.

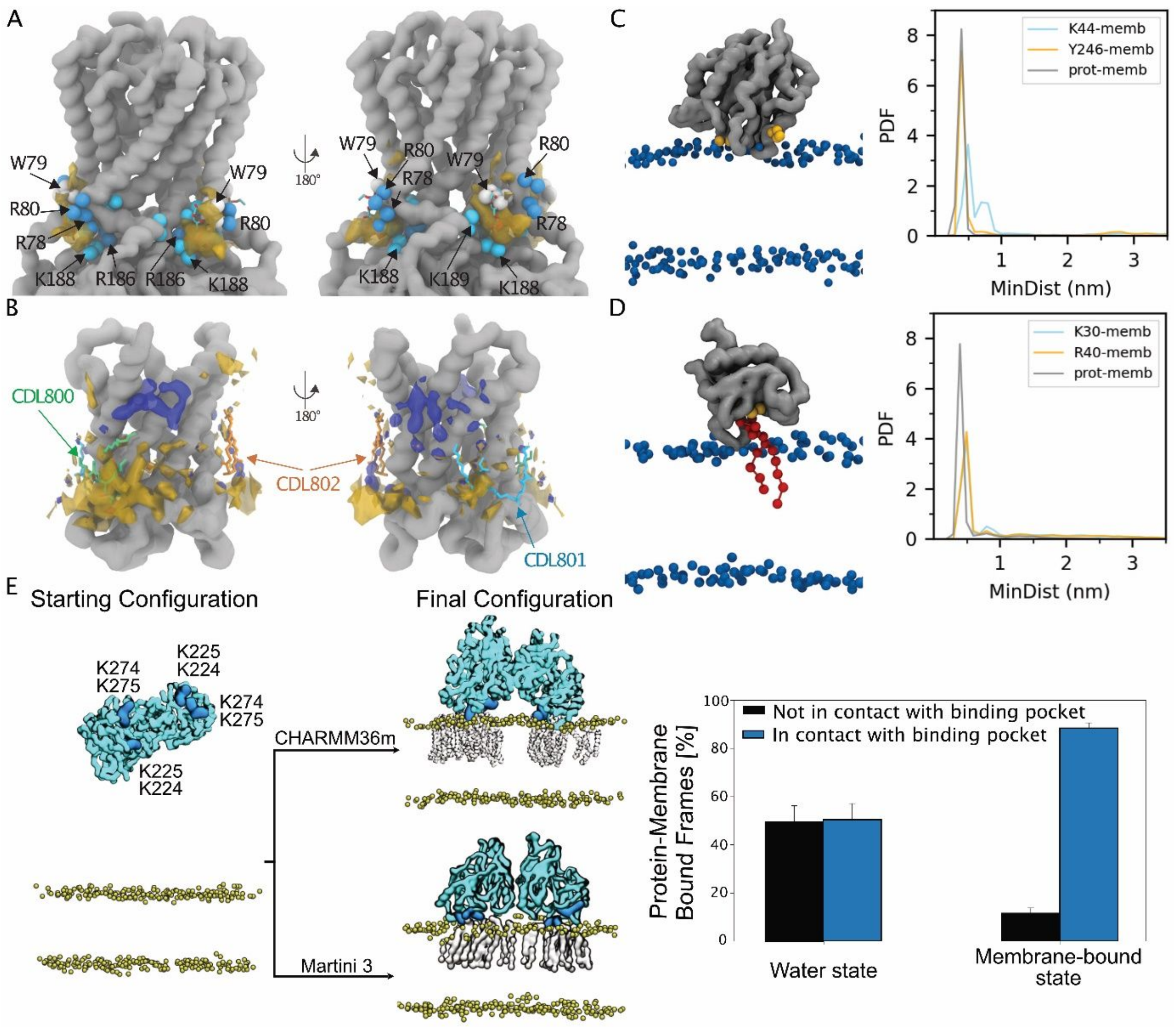

Figure S9. Lipid-protein interaction studies are facilitated by the Martini 3 lipidome. *(A-B) POP6 lipids and cardiolipins near transmembrane proteins. (A) POP6 lipids arrangement around the Kir2.2 channel. Fractional occupancy maps for POP6 lipids, calculated over the 30 µs-long simulation, are shown at density isovalues of 0.2 (red) and 0.05 (yellow), on a scale from 0 to 1. Arginine, lysine, and tryptophan residues surrounding the sites are shown as dark cyan, light cyan and white spheres, respectively. The protein structure corresponds to the last frame of the simulation, and the backbone is shown as a gray surface. Shown as licorice are the four PI(4,5)P_2 headgroups resolved in the experimental structure (PDB ID 6M84), which was superimposed to the last frame of the simulation. (B) Cardiolipin and POPC lipids arrangement near the bovine ADP/ATP carrier. Fractional occupancy maps from one of the two Martini 3 simulations (B) are shown for cardiolipins (POCL) at isovalues of 0.2 (red) and 0.1 (yellow), and for POPC lipids (in blue) at isovalue of 0.1 around the ADP/ATP carrier. The CG structure corresponds to the last frame of the simulation, and superimposed is the experimental structure (PDB ID 1OKC), with the three bound cardiolipins shown in green (CDL800), cyan (CDL801), and orange (CDL802) sticks. (C-D) Membrane binding of BtPI-PLC (C) and PLC-d1 PH domain (D). Snapshots from simulation systems showing (C) BtPI-PLC and (D) the PLC-d1 PH domain bound to the corresponding membranes. The protein backbone is shown as a gray surface; phosphate beads are shown as blue spheres, and selected membrane binding residues as yellow spheres. The bound PIP*

lipid is shown in red sphere-stick representations. Probability Density Function (PDF) of minimum protein-membrane distances and minimum distances between selected protein residues with the membrane. PDFs were calculated across all the replicates. (E) The left panel displays the system configuration where the VP40 dimer was positioned more than 2 nm away from the membrane in 10 different orientations, with each replica simulated for 10 µs. The final frames of the protein-membrane interaction for both the atomistic model and the Martini 3 model are shown for comparison. The right panel shows the percentage of frames in which the protein is in contact with the experimentally identified binding pockets (K224, K225, K274, and K275) or not, relative to the total number of frames where the protein interacts with the membrane.

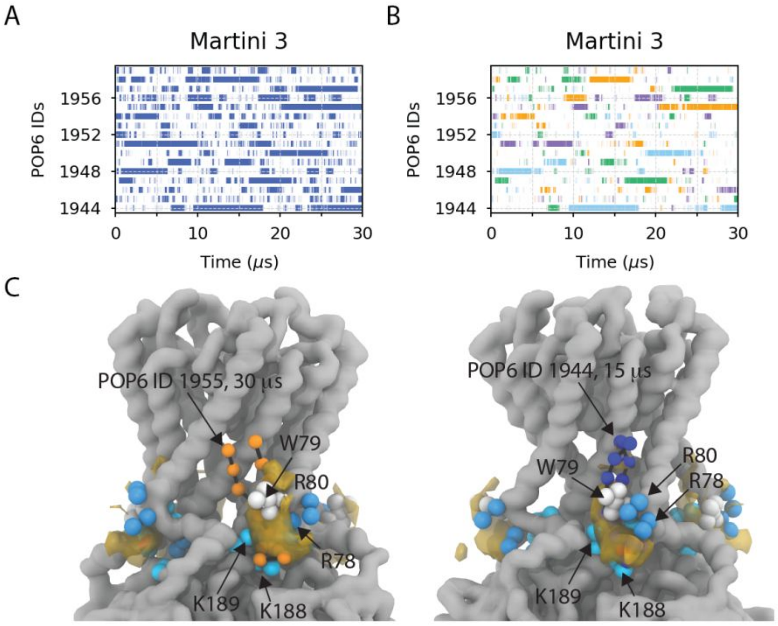

Figure S10. *POP6 lipids in close contact with the Kir2.2 channel. POP6 lipids that as a function of time are found within 5 Å of (A) protein residues and (B) each of the four PI(4,5)P_2 binding sites (colored in cyan, orange, green and purple). In B, the minimum distance from each site was measured considering residues R78, W79 and K188. C. Snapshots at 30 μs (left) and at 15 μs (right) with two POP6 lipids at the corresponding binding sites (POP6 ID 1955, shown with orange spheres, and POP6 ID 1944, shown with dark blue spheres). The fractional occupancy maps for POP6 lipids of Figure S9 are also shown as a reference. Arginine, lysine and tryptophan residues surrounding the sites are shown as dark cyan, light cyan and white spheres, respectively.*

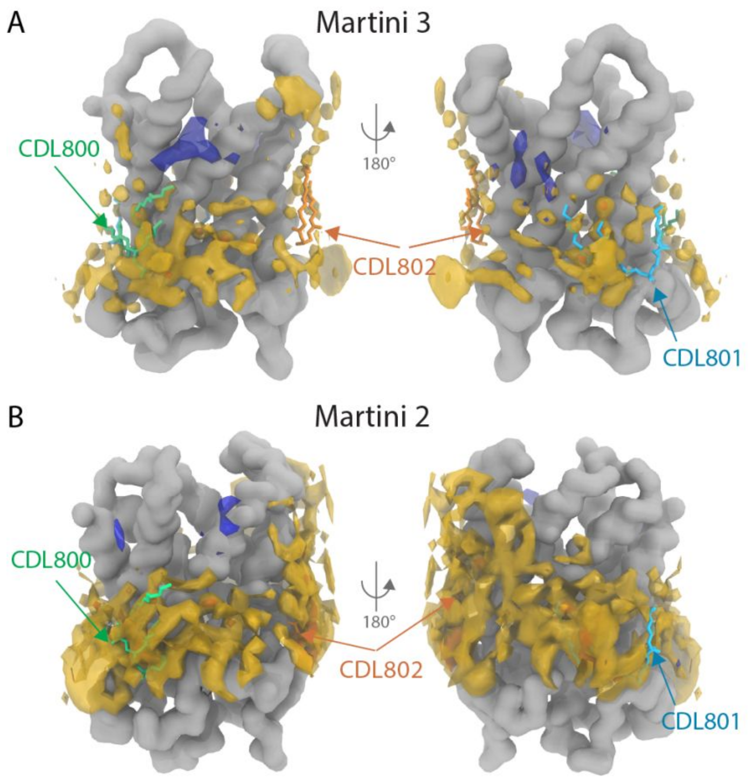

Figure S11. *POCL and POPC arrangement around the ADP/ATP carrier (PDB ID 1OKC). Fractional occupancy maps resulting from a second 30 µs-long Martini 3 (A) and a Martini 2 (B) simulation. Maps are shown for cardiolipins (POCL) at isovalues of 0.2 (red) and 0.1 (yellow), and for POPC lipids at the isovalue of 0.1 (blue) around the ADP/ATP carrier. The CG structure corresponds to the last frame of the simulation, and superimposed is PDB ID 1OKC, with the three bound cardiolipins shown in green (CDL800), cyan (CDL801) and orange (CDL802) sticks.*

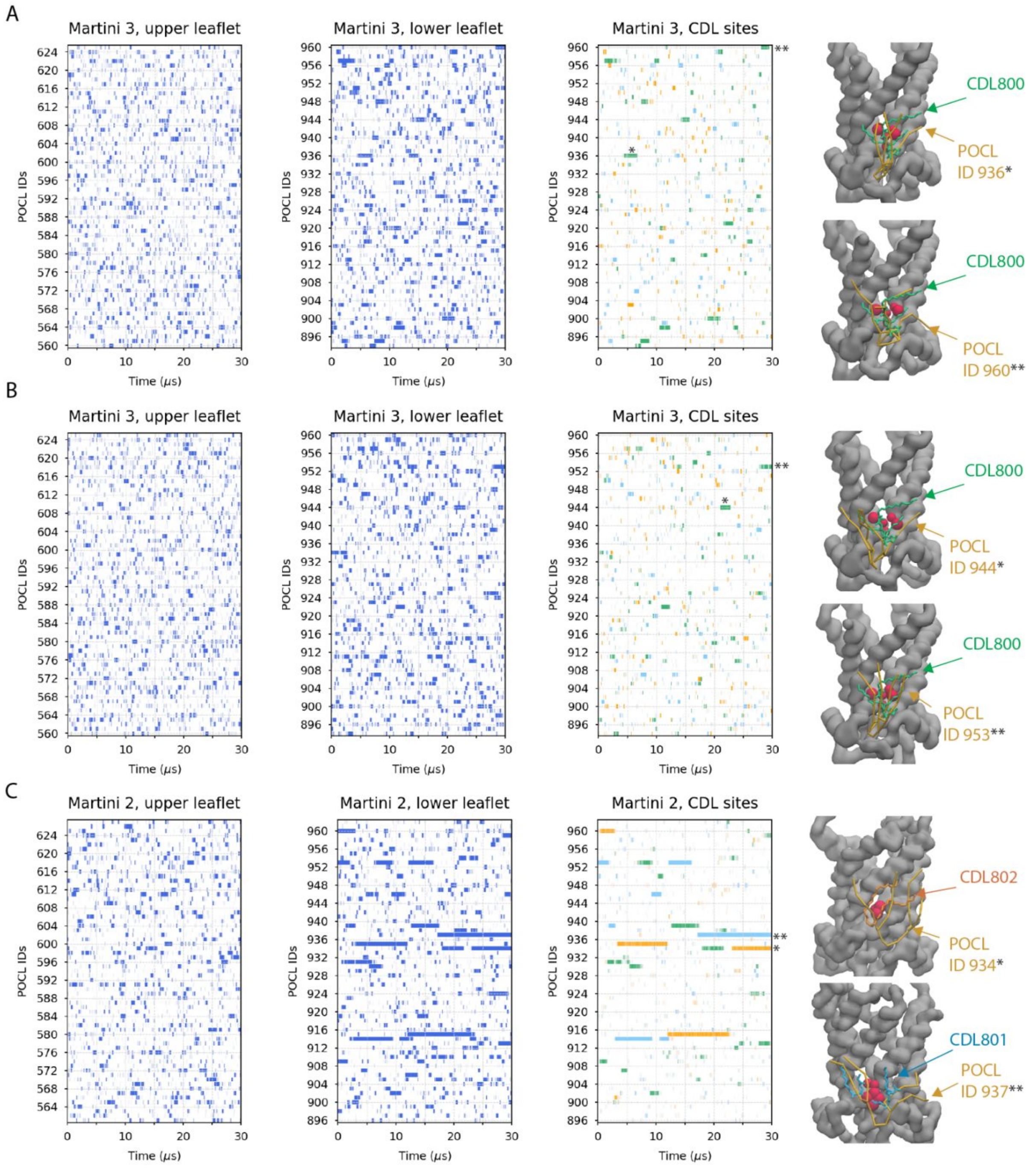

Figure S12. *Cardiolipins (POCL) in close contact with the ADP/ATP transporter as a function of time in two Martini 3 (A and B) and one Martini 2 (C) simulations. The first and second panel show the POCL molecules that are within 5 Å of the protein, for both upper and lower leaflet. The third panel for each simulation shows POCL molecules within 5 Å of the experimental CDL sites, with CDL800 in green, CDL801 in blue and CDL802 in orange. Snapshots of POCL molecules at the CDL sites are shown in the right most panel. The minimum distance for each CDL site was calculated with respect to residues 72-74 for CDL800, 272-274 for CDL801 and residue 176 for CDL802, shown as red spheres.*

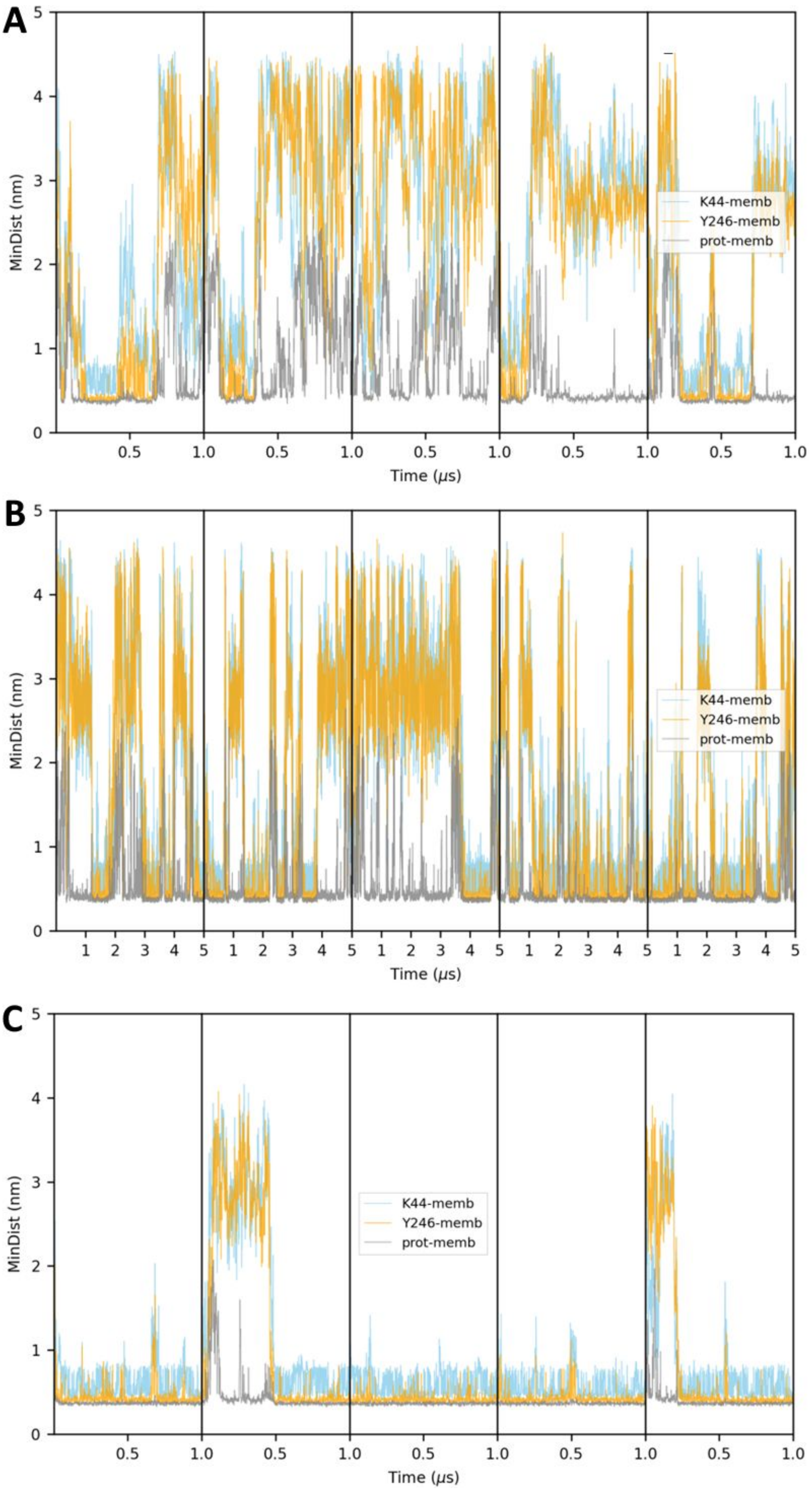

A
B
C
MinDist (nm)
Time (μs)
K44-memb
Y246-memb
prot-memb

Figure S13. *(A) Interaction of BtPI-PLC with a DMPC membrane. None of the replicates led to binding in the right orientation for a long time. (B) Interaction of BtPI-PLC with a DMPC membrane over longer timescales. These simulations were performed independently from the previous sets to test timescale effects. (C) Interaction of BtPI-PLC with a POPC membrane. The protein binds correctly in all the replicates. Even when the initial interaction with the membrane was via the non-binding face of the protein, the protein reoriented on the membrane surface to find the correct binding mode (panels B and E).*

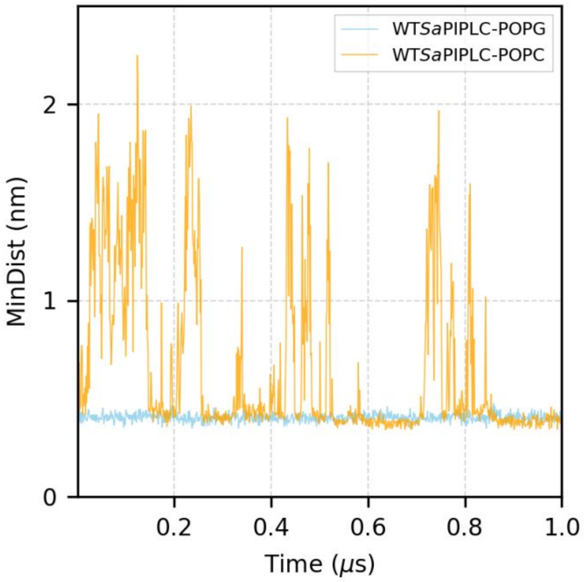

Figure S14. *Interaction of wild-type SaPI-PLC with a POPC (negative control) and POPG (positive control) membrane. Both the cases lead to the desired outcome meaning no binding with POPC and binding with POPG.*

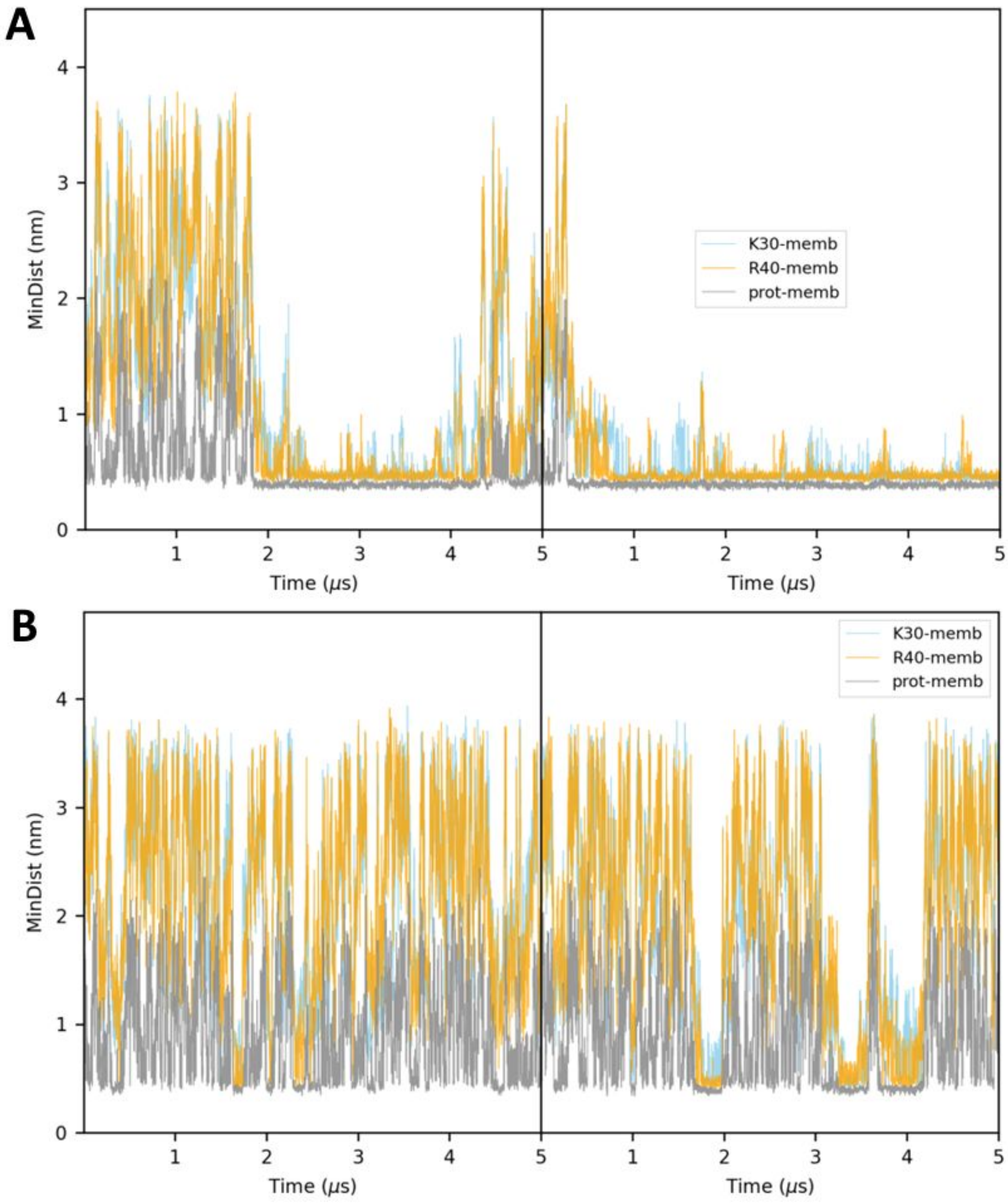

Figure S15. *(A) Interaction of the PH domain with a bilayer containing POPC:POPS:PIP$_2$:PIP$_3$ (73:20:5:2). Both replicates led to membrane binding in the right orientation. (B) Interaction of the PH domain with a bilayer containing POPC:PIP$_2$ (95:5). The two replicates did not lead to membrane binding in the right orientation or complex formation for long time scales.*

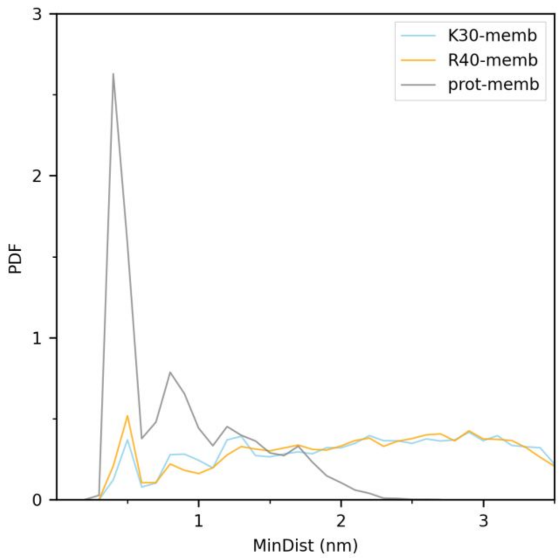

Figure S16. *Distributions of minimum protein-membrane distances and minimum distances between selected membrane binding residues with the membrane for PH domain with a bilayer containing POPC:PIP$_2$ (95:5). These distributions highlight the non-binding events.*

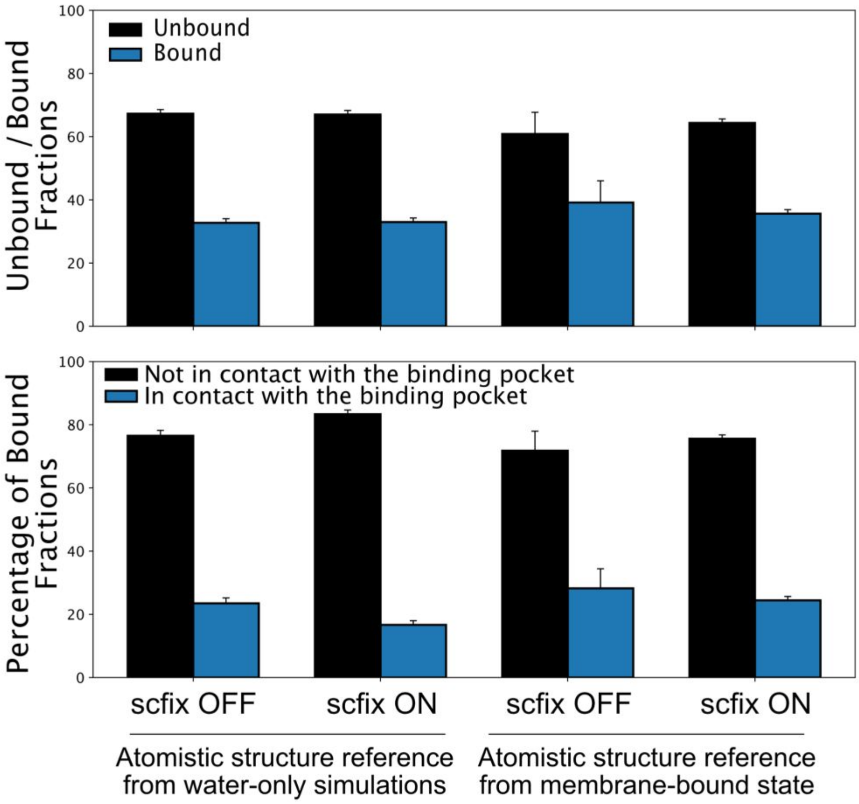

Figure S17. *Analysis of the interaction between FGF2 and the membrane. In the upper panel, the unbound and bound fractions were calculated by determining the contact occupancy. The bottom panel illustrates the percentage of frames in the bound fraction where the protein was in contact with the experimentally known binding pockets (K127, R128 and K133).*

Supplementary Discussion H) Limitations of the Martini Model and Future Directions

Despite the considerable advancements achieved with the reparametrized Martini 3 lipidome in accurately representing lipid membranes, several areas require further refinement to broaden its applicability and enhance model accuracy. Here, we outline possible future directions for the Martini lipidome.

First, one of the compromises we made for the presented lipid parameter set, was a small systematic decrease in APL across most lipid types in favor of drastically improved phase transition behavior. One axis requiring further exploration is the properties of the phosphate bead, for which we currently use the Q5 bead, a bead type assignment which is shared with other chemical groups such as carboxylates. It is possible that phosphate could be independently optimized to represent the distinct behavior of phospholipids. Additionally, fine-tuning the interactions between the phosphate and metal ions, like calcium[100], could also be explored to improve the model's accuracy. Such parameterization could focus on accurate reproduction of experimental APL values while preserving the improved phase transition properties achieved with the current models. Additional refinements of the Martini 3 bead interaction matrix, specifically for the lipid headgroup region, are expected to further improve consistency with experimental and atomistic benchmarks.

Second, while Martini 3 lipids show compatibility with protein-lipid interaction patterns observed with Martini 2 lipids, further validation is needed. Martini 2 has been extensively tested for diverse protein-membrane systems, but forthcoming improvements in Martini 3 protein models, such as improved side chain thermodynamics and structural flexibility, will require re-evaluation of these interactions. A systematic assessment of the Martini 3 lipidome together with updated Martini 3 protein models will be essential to ensure compatibility across a wide range of systems. In this work, we have established a set of test cases that could be iterated in future parameterization work, and we encourage the broader Martini community to report both positive and negative results of the protein-lipid interactions studied.

Third, while the lipidome described here is significantly expanded to thousands of lipids, it is still incomplete. Important classes of lipids, such as glycolipids, branched

lipids, and specialized signaling lipids, remain absent. The inclusion of glycolipids will benefit from the development of Martini 3-compatible sugar parameters[101]. Further optimization of the lipidome would benefit from leveraging automated parameterization tools such as Bartender[102], CGCompiler[103], and Swarm-CG[104]. These workflows allow systematic refinement of lipid models while incorporating critical properties, such as phase behavior, directly into the cost function. By automating parameter optimization, the Martini lipidome can be expanded to include new lipids more efficiently, facilitating the exploration of novel lipid structures and a broader range of biological membranes.

Finally, it is important to acknowledge the inherent limitations of coarse-grained modeling, which also apply to the Martini 3 lipidome. For instance, the entropy-enthalpy compensation limits the transferability of the lipid models to state points other than those used in the parametrization process. The entropy-enthalpy tradeoff also substantially complicates reproduction of certain delicate processes like bilayer pore formation, where free energy barriers are too high in Martini[105]. While the reparametrized Martini 3 lipidome slightly improves this property over Martini 2 (Figure S18), the free energy profile of pore formation is still significantly larger compared to atomistic models. Furthermore, although the current Martini lipidome can capture phase transitions quite well, in general, capturing temperature dependent behavior will remain challenging. A temperature-dependent approach, as suggested in earlier works[106, 107], could mitigate some of these issues and improve the robustness of Martini simulations across a wider range of conditions. Another issue pertains to the artificially enhanced dynamics of CG models. Due to the neglect of atomic degrees of freedom, friction is being reduced, leading to a speedup in the overall dynamics[108]. For the new Martini lipidome, we do not expect a much different speedup factor compared to previous lipid models, as seen by similar lipid diffusion constants between Martini 2 and 3 models (Figure S3).

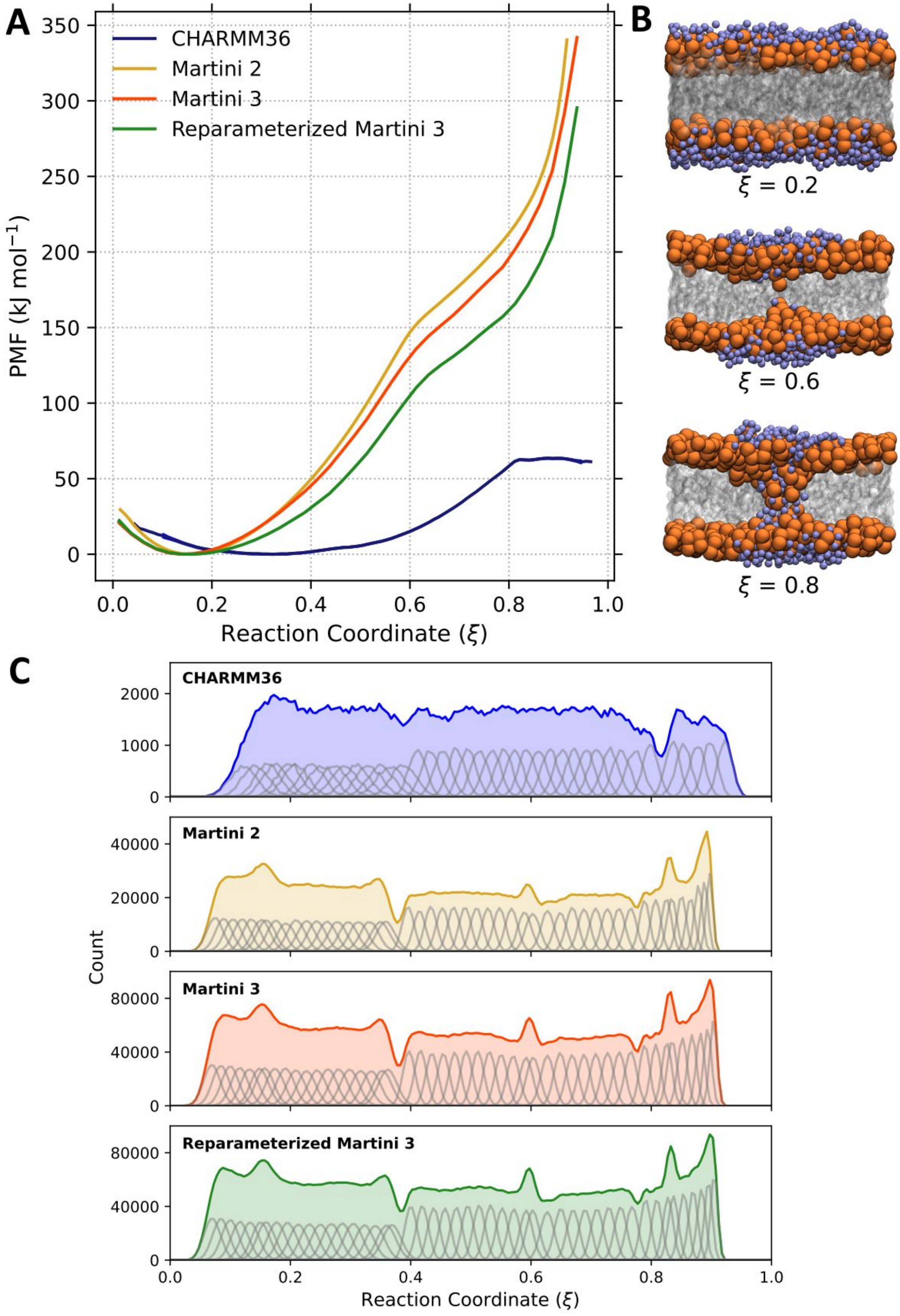
A
B
C
CHARMM36
Martini 2
Martini 3
Reparameterized Martini 3
PMF (kJ mol^{-1})
Reaction Coordinate (ξ)
ξ = 0.2
ξ = 0.6
ξ = 0.8
Count
0.0
0.2
0.4
0.6
0.8
1.0
0
50
100
150
200
250
300
350
1000
2000
20000
40000
80000

Figure S18. (A) Potential Mean Force (PMF) of pore formation in atomistic and coarse-grained DPPC membrane systems along pore formation coordinate ξ; systems were simulated with CHARMM36 (blue), Martini 2 (yellow), original Martini 3 (orange), and reparametrized Martini 3 (green) lipid models. Curve uncertainty (in the single-digit kJ mol^{-1} range) is represented by shading in the same colors along the corresponding curve (for some points at the edges of the reaction coordinate range an error could not be estimated). Onset of a water-continuous membrane defect occurs at the PMF inflection points: close to ξ=0.8 for the all-atom system and ξ=0.6 for the Martini systems. While pore formation is overall too energetically unfavorable under Martini, the Martini 3 lipids, and especially the reparametrized models, have improved significantly on the energy difference to atomistic values. (B) Snapshots of the DPPC membrane systems with the reparametrized lipid model along the reaction coordinate; lipid PO4 beads are represented in orange and water beads in blue (only those in the vicinity of the lipid headgroups or the established defect are represented); the rest of the membrane is represented as a translucent surface. (C) Pore-formation reaction coordinate coverage in the umbrella sampling simulations. Histograms show the coverage of each individual window (gray lines) as well as the overall sum for the entire system (in color).

For both AA and CG pore simulations, we used the GROMACS[109] 2018 simulation package to run the lipid pore umbrella sampling simulations. We followed the reaction coordinate described by Hub and Awasthi[110] implemented using PLUMED[111] version 2.5.0. 40 or 48 umbrella windows were used per AA or CG system, respectively, with force constants between 5000 and 17000 kJ mol^{-1}. Simulation times were 66 ns for the AA systems and between 730 and 2100 ns for the CG systems (reaction coordinates were sampled every 10 ps for AA systems and every 20 ps for CG ones). The same pore reaction coordinate settings were employed for AA and CG systems: the coordinate was computed over 20 cylindrical slices, of 0.2 nm thickness and 1.2 nm radius, with a slice occupancy switch function ζ parameter of 0.25. Because of the different resolutions, the reaction coordinate values are not directly comparable between AA and CG; still, the energy differences between PMF minima (the unbiased membrane state) and the inflection points (onset of defect formation) can be compared.

The CHARMM36 force field[112] was used for all AA pore simulations. Membranes were built using CHARMM-GUI[113, 114] and equilibrated according to its protocol. Systems had 144 DPPC lipids per leaflet, solvated with ~13300 TIP3P water molecules with 150 mM NaCl ionic strength, in a 9.5×9.5×7.5 cubic box. Particle-Mesh Ewald[115] (PME) summation was used to compute electrostatics and Van der Waals interactions were switched off smoothly from 1.0 to 1.2 nm. The Verlet list scheme was employed to update the particle neighbor list. A Nosé-Hoover[116] thermostat with a coupling time of 1.0 ps was used to maintain the temperature at 323 K. A semi-isotropic pressure coupling to 1.0 bar was employed using the Parrinello-Rahman[117] barostat with a relaxation time of 5.0 ps.

The PMF was estimated using the WHAM program[118] by Alan Grossfield version 2.0.11.

Supplementary Methods I) Reference Simulations

All-atom Reference Simulations

The atomistic reference simulation data included three sets of alkanes with a length of 16, 20, and 24 carbon atoms together with 5 different alkenes with a length of 20 carbon atoms, with both mono and poly-unsaturated chains, mimicking common lipid tails; glycero-, ether-, and sphingomyelin phospholipid bilayers of pure composition of POPC (16:0/18:1), POPE (16:0/18:1), POPG (16:0/18:1), POPS (16:0/18:1), PAPC (16:0/20:4), PDPC (16:0/22:6), PSM (d18:1/16:0), SSM (d18:1/18:0), DMPCE (e14:0/e14:0), DHPCE (e16:0/e16:0) and plasmalogen PLC18 (ve18:0/18:1);

ceramide PCER (d18:1/16:0) in bulk; monolein (18:1) in bulk; di- and triglycerides in three different environments (bulk, bi-phasic system with water, and inside a POPC bilayer) of triolein (18:1/18:1/18:1) and diolein (18:1/18:1) kindly provided by Campomanes et al.[18].

Hydrocarbon and Neutral Lipids Reference Simulations

We simulated in bulk three alkane types with a length of 16 (hexadecane), 20 (eicosane), and 24 carbon atoms (tetracosane) together with 5 different alkenes with a length of 20 carbon atoms, with both mono and poly-unsaturated chains, mimicking common lipid tails (9Z-eicosene, 6Z,9Z-eicosadiene, 3Z,6Z,9Z-eicosatriene, 3Z,6Z,9Z,12Z-eicosatetraene, 3Z,6Z,9Z,12Z,15Z-eicosapentaene). Monolein (18:1) and ceramide (d18:1/16:0) systems were simulated using parameters obtained from the CHARMM-GUI web server[113, 119]. The hydrocarbon molecules were parameterized using the CHARMM36 CGenFF force field[112] version 2.5. For each hydrocarbon, monoolein (MO), or ceramide (PCER), 200 identical molecules were inserted into a cubic box of volume ≈ 5.5 nm^3. Generic CHARMM36 simulation settings were used: Van der Waals non-bonded interactions were switched from 1.0 nm to a cutoff of 1.2 nm. Particle Mesh Ewald[115] (PME) summation was used for electrostatics with a real-space cutoff of 1.2 nm. Simulations were integrated using 2 fs steps and hydrogen bonds were restrained for all systems using LINCS[120]. The systems were minimized and equilibrated for 10 ns at 300 K and 1 bar using the Nose-Hoover thermostat (τ_t = 1) and Berendsen barostat (τ_p = 4)[116, 121]. Production simulations were 10 ns at 300 K and 1 bar using the Nose-Hoover thermostat (τ_t = 1) and Parinello-Rahman (PR) barostat[117] (τ_p = 5). Simulations were performed using GROMACS[109, 122] v2019.4.

Di- and triglycerides reference simulations were kindly provided by Campomanes et al.[18] and includes diolein (DO, 18:1/18:1) and triolein (TO, 18:1/18:1/18:1), simulated in three different environments: bulk, bi-phasic system with water, and inside a POPC bilayer using the CHARMM36-p parameters.

Phospholipid Reference Simulations

Phospholipid bilayers of pure composition were prepared using the CHARMM-GUI web server: POPC (16:0/18:1, 1-palmitoyl-2-oleoyl-*sn*-glycero-3-phosphocholine), POPE (16:0/18:1, 1-palmitoyl-2-oleoyl-*sn*-glycero-3-phosphoethanolamine), POPG (16:0/18:1, 1-palmitoyl-2-oleoyl-*sn*-glycero-3-phosphoglycerol), POPS (16:0/18:1, 1-

palmitoyl-2-oleoyl-*sn*-glycero-3-phosphoserine), PAPC (16:0/20:4, 1-palmitoyl-2-arachidonoyl-*sn*-glycero-3-phosphocholine), PDPC (16:0/22:6, 1-palmitoyl-2-docosahexaenoyl-*sn*-glycero-3-phosphocholine), DMPCE (e14:0/e14:0, 1,2-di-O-tetradecyl-*sn*-glycero-3-phosphocholine) DHPCE (e16:0/e16:0, 1,2-di-O-hexadecyl-*sn*-glycero-3-phosphocholine), PLC18 (ve18:0/18:1, 1-octadecenyl-2-oleoyl-*sn*-glycero-3-phosphocholine), PSM (d18:1/16:0, N-palmitoyl-D-*erythro*-sphingosylphosphorylcholine), SSM (d18:1/18:0, N-stearoyl-D-*erythro*-sphingosylphosphorylcholine).

The CHARMM36 force field was used for all reference phospholipid simulations[18, 123-125]. Each bilayer consists of 200 lipid molecules with a water layer of 2.25 nm on top and bottom of the bilayer. For the anionic PG and PS lipid systems, 200 sodium counter ions were added to the solution but otherwise, the systems were simulated without salt. The systems were first minimized and equilibrated in a series of small equilibrations, with gradually weaker restraints on protein and lipids using generic CHARMM-GUI simulation settings. Production simulations were 500 ns long at 310 K (POPC, POPE, POPG, POPS, PAPC, PDPC, PLC18), 323 K (PSM, SSM) or 333 K (DMPCE, DHPCE) and 1 bar using the v-rescale thermostat (τ_T = 1 ps) and Parrinello-Rahman barostat (τ_p = 5 ps) using semi-isotropic pressure coupling (Bussi, 2007). The non-bonded settings were identical to the hydrocarbon simulations. Simulations were performed using GROMACS v2019.4.

Supplementary Methods J) Evaluation of Benchmarks

Evaluating the Martini Lipid Benchmark

We set up lipid bilayers of 200 lipids (100 in each leaflet) in a cubic periodic box of dimensions ≈ 8 nm x 8 nm x 10 nm using *insane*[13], matching each lipid type and temperature for each experimental data point in the Martini lipid Benchmark (the benchmark data can be found at GitHub: https://github.com/Martini-Force-Field-Initiative/M3-Lipid-Parameters/tree/main/Martini_lipid_Benchmark). The systems were solvated with regular water beads. No ions were added for phosphocholine (PC), phosphoethanolamine (PE), and sphingomyelin (SM) systems. 200 Na^+ counter ions were added to the phosphoglycerol (PG) systems. A generic sample GROMACS

simulation settings file (.mdp) with default Martini 3 settings can be found on our GitHub page (https://github.com/Martini-Force-Field-Initiative/M3-Lipid-Parameters/tree/main/tools/resources). For each system, the simulation temperature matches the experimental temperature using the v-rescale thermostat (τ_T = 1 ps) and we maintain 1 bar pressure using the c-rescale barostat (τ_p = 4 ps) using semi-isotropic pressure coupling for both equilibration and production simulations[126]. Each system was minimized and equilibrated for 50 ns, followed by a 100 ns production simulation. Frames were saved each 1 ns. Simulations were performed using GROMACS v2024.0.

APL was calculated using FatSlim[127] v0.2.2. DHH is the electron density peak-peak distance between bilayer leaflets in a scattering experiment, along the surface normal going through the bilayer. The peaks are usually located close to the phosphate regions, due to the high density of electrons of the phosphorus and oxygen atoms[128]. The average distance between phosphate beads (PO4-PO4) is often used when comparing the bilayer thickness of CG simulations to experimentally determined DHH[104]. However, the DHH and PO4-PO4 distances are in general not equal because phospholipid headgroups are dynamic and the headgroup is often located at the same plane as the phosphate region, which slightly changes the location of the electron peak[129]. Instead, we calculate the DHH directly from the CG simulation by assigning the appropriate number of electrons to each CG bead and calculating the peak-peak distance from the resulting electron density (using *gmx density* in GROMACS). We find this protocol leads to better agreement with DHH values obtained from SDP modeling compared to using the PO4-PO4 distances. The electron density was smoothened using a convolution window of 5 ns (5 frames).

DB is another measure of bilayer thickness that provides information on the water-excluded volume of the bilayer[130] and is therefore an important measure of the water penetration into the lipid bilayer. The Gibbs dividing surface between the bilayer region and the water region is defined as the location DB/2 (focusing on a single leaflet)[128]. DB can be calculated from the water density $P(W)_z$ across the bilayer:

$$DB/2 = D_z - \int_0^{D_z} P(W)_z dz \tag{4}$$

where 0 is the center of the bilayer and D_z is a point in the water region where $P(W)_z$ = 1. In our calculations, DB is integrated over both leaflets simultaneously to avoid

defining a bilayer center. The water density was smoothened using a convolution window of 5 before integration.

Finally, the hydrophobic thickness 2Dc reports on the thickness of the aliphatic hydrocarbon region of the bilayer[128]. Here, we calculate the full width at half maximum (FWHM) of the number density of the apolar tail beads (starting immediately after the glycerol ester bead). The number density was smoothened using a convolution window of 5 ns (5 frames).

Block averaging using 10 blocks (10 ns per block) was used to determine uncertainties of APL, DHH, DB, and 2Dc quantities.
We compare the experimentally derived bilayer properties to the equivalent properties obtained from CG simulations through a mean normalized root-squared error (MNRSE) measure:

$$MNRSE = \frac{1}{N}\sum_{i=1}^{N}\frac{\sqrt{(X_{i,exp}-X_{i,sim})^2}}{\sigma_{i,exp}} \qquad (1)$$

We report MNRSE calculated across all data points and for each lipid headgroup class.

Estimation of Bilayer Bending Moduli

The bending modulus (k_c) quantifies the amount of energy that is required to deform a bilayer membrane from its spontaneous curvature and is an important physical constant for characterizing membranes mechanical properties. Two different methods were used to estimate k_c: Real-space fluctuation (RSF) of lipid tilt and splays[5, 131] and simulated buckling[7]. As for the RSF method, we used a published Python implementation[132]. Bilayer patches of 169 lipids per leaflet were built with *insane*[13] and simulated using GROMACS 2024.0 for 10 µs, with the 5-10 µs portions utilized for analysis. The DOPG system contained one sodium ion per lipid to achieve charge neutrality.

For the buckling approach, we used in-house scripts[8]. We used *insane*[13] to build bilayers with size (32 x 8 x 20) nm. The total number of lipids per system was 924. After equilibration, we carried out an equilibrium simulation to calculate the length of the uncompressed bilayer. We then compressed the bilayer by 30% in the x dimension, inducing the formation of a buckle (while keeping the y dimension fixed).

Each system consisted of two production runs, starting from both the uncompressed and buckled bilayers. The uncompressed bilayer production run was under anisotropic conditions, with compressibility set to 0 in the y-dimension such that the bilayer length can only fluctuate in the x-dimension. The buckled bilayer production run was under semi-isotropic conditions, with compressibility in and x y set to 0, fixing the buckle in place. The bending modulus can be calculated from the force exerted by the buckled membrane in the x-dimension that is proportional to k_c according to:

$$F_x(y) = 4\pi^2 k_c \frac{L_y}{{L_x}^2} \sum b_i \gamma^i \quad (5)$$

where γ is the strain, L_x is the length of the system along x without compression, L_y is the system length in y, and b_i is a set of coefficients described by Hu et al.[7]. For each bilayer we carried out 5 production runs of 8 μs each, the errors reported are the standard errors considering the 5 independent samples. At the time of simulation, GROMACS did not support the c-rescale barostat for fully anisotropic pressure coupling, and so the Parinello-Rahman barostat[117] was used instead.

Systematic Generation and Testing of Martini 3 Lipid Topologies

The lipid topology generation script[13] was updated to create Martini 3 lipid topologies following the new lipid mapping scheme defined in this manuscript, as well as for PIP headgroup parameters[70]. For a given input of headgroup, linker, and tail specification the lipid topology generator assembles the complete lipid parameters. Currently, the script supports both glycerol and sphingosine backbones, tail beads for small (representing two carbon) and normal (representing four carbons) beads that are saturated, have one cis or trans double bond or more than one double bond, as well as headgroup types to build choline, ethanolamine, glycerol, serine, phosphatidic acid and several PIP variants. Note, due to the flexibility of the building script nonsensical lipids can be built and all generated parameters should be tested and used with care.

A set of common lipids was generated, combining 5 headgroups: phosphatidylcholines (PC), phosphatidylethanolamine (PE), phosphoglycerol (PG), phosphatidylserine (PS), and phosphatidic acid (PA) lipids (here all used with -1 charge) with 40 different acyl chain combinations of varying length and saturation (see tail nomenclature in SI Table S1). Additionally, 18 different PIPs varying in headgroup and tail composition as well as sphingomyelin and ceramides each with 10 different tail combinations were included.

Each lipid type was simulated with 100 lipids in each leaflet, in either pure, or 20% in POPC, DLPE, or SSM-CHOL membranes. The membrane builder *insane*[13] was used to setup all systems, recently extended to handle both Martini 3 and 2 lipid nomenclature[133] (https://github.com/Tsjerk/Insane). The initial box size was 8 nm x 8 nm x 10 nm and solved with water, neutralizing ions and 150 mM NaCl. Eeach system ran for 1 μs at 310 K and the first 200 ns were excluded from analysis. For comparison, available Martini 2 lipids[13, 14] corresponding to any of the tested Martini 3 lipids were also simulated following the same protocol. Note, some Martini 2 analysis is shown in duplicate as the same Martini 2 lipids can correspond to up to two Martini 3 lipids due to the finer acyl chain granularity (2 carbon increments compared to 4 in Martini 2).

For each system eight different properties were calculated: four different bilayer thickness values (PO4, DHH, DB, 2Dc) and APL. Additionally, the bilayer area compressibility modulus (K_A), the P_2 lipid tail order parameter and lipid diffusion were calculated. K_A was calculated from the amplitude of the box area fluctuations or $K_A = kT\langle A\rangle/(N\langle(A - A_0)^2\rangle)$, where A is the box area, A_0 is the equilibrium area, N is the number of lipids pear leaflet, and kT is the Boltzmann constant and temperature in kelvin. The P_2 lipid tail order parameter was calculated from the angle between the normal of the bilayer surface and the vector along each bond in the lipid tails (θ), as $P_2 = ½\ (3\cos^2\theta - 1)$ and results for different tail bonds and leaflets were averaged. Lipid lateral diffusion coefficients were calculated with diffusion-GLS[134]. Diffusion-GLS uses a generalized least squares estimator to provide an optimal trade-off between potential systematic errors due to non-diffusive dynamics at short time lags, and statistical errors due to increasing uncertainties in the MSD values at longer time lags. Prior to analysis, the trajectories of the lipid centers of mass were unwrapped using an updated unwrapping scheme[135] which preserves the diffusive character of the wrapped trajectory[136]. For each property the average over the last 800 ns was reported with SE, evaluated with block averaging.

Scripts for lipid topology generation, automated system setup, and analysis are available on GitHub (https://github.com/Martini-Force-Field-Initiative/M3-Lipid-Parameters/tree/main/tools).

Supplementary Methods K) Construction of Complex Lipid Bilayers in Martini 3

Coarse-Grained Molecular Dynamics Simulation Systems of Complex Bilayers

A mammalian plasma membrane model composed of eight different lipid species asymmetrically distributed across the leaflets was built with *insane*[13] in a 40 nm × 40 nm × 10 nm simulation box and solvated with water and 0.15 M NaCl (see Table S5). While maintaining the preset ratio between the phospholipids, 313 randomly selected lipids in the cytoplasmic leaflet were removed to obtain equal surface areas of the leaflets, as described in Ozturk et al.[133]. A total of 4 independent simulation systems were built, each composed of a total of ~128K CG beads.

After applying 1500 steps of energy minimization, the simulation system was equilibrated for about ~2 ns with increasing time steps (from 1 fs to 20 fs, see Table S6 for more details). The production run for each replica was 20 µs. During the equilibration and production runs, LINCS[120] constraints were used for ring systems and stiff bond constraints; the LINCS order and iteration were set to 12 and 2, respectively. The temperatures of lipids and solvent (NA, CL, and W beads) were separately kept at 310 K with a time constant for coupling (τ_T) of 1.0 ps, using the Berendsen[121] and v-rescale[137] thermostats during the equilibration and production, respectively. The pressure was kept at 1 bar initially using Berendsen[121] and Parrinello-Rahman[117] barostats during the equilibration and production steps, respectively. The compressibility was set to 3×10^{-4} bar^{-1}. The cut-off distance for the short-range neighbor list was set to 1.35 nm for proper neighbor list updates, as suggested by Kim et al.[138] z-coordinate positional restraints were applied to the phosphate beads of POPC lipids only in the upper leaflet, with a force constant of 2 $kJ/mol/nm^2$, to maintain a flat bilayer as explained in earlier work[133].

All-Atom Molecular Dynamics Simulation Systems of Complex Bilayers

The last snapshot of the 20 µs-long trajectory of the smaller plasma membrane patch simulated from an earlier study[133] was converted to an all-atomistic representation using *ezAlign*[139] with its default options. The resulting atomistic systems were composed of ~27.9K particles. Using these initial coordinates, three independent simulation systems were built, energy-minimized for 500 steps, and equilibrated

following the six-step equilibration protocol suggested by CHARMM GUI's membrane builder[114] with the modification that the number of steps in each step was doubled. Finally, following the same protocol, a 100 ns-long production was carried out at 310 K and 1 bar.

Analysis of Complex Bilayers

The density profiles of the following beads were computed using the *gmx density* command: chloride (CL), phosphate (PO4 in the Martini models and P in the CHARMM36 model), sodium (NA), water (W), cholesterol polar bead (ROH in the Martini models and O3 in the CHARMM36 model), phospholipid linkers (GL1, GL2, OH1, AM2 in the Martini models and C21, C31 in the CHARMM36 model) and phospholipid tails (C1A, C2A, C3A, C4A, D1A, D2A, D3A, T1A, C1B, C2B, C3B, C4B, D2B, and D3B in the Martini models and all heavy atoms in the lipid tails for the CHARMM36 model). The lipid enrichment/depletion indices, membrane thickness, cholesterol flip-flop rate and order parameter were calculated with LiPyphilic[64], and for the analyses the mid-plane cutoff distance to assign cholesterols to cytoplasmic and extracellular leaflets was set to 0.7 nm. The cholesterol flip-flop rate was computed with LiPyphilic's FlipFlop module[64], while the membrane thickness was calculated as the peak-to-peak distance of lipid headgroup (PO4 beads in Martini models and P atoms in the CHARMM36 model) density in the membrane normal using LiPyphilic's Membrane thickness module[64]. Lipid enrichment/depletion indices were computed with LiPyphilic's Neighbours module[64] using a distance cutoff of 1.5 nm to count neighboring lipids based on the position of the linker (GL1, GL2, OH1, AM) beads for phospholipids and ROH beads for cholesterol, as explained in a previous work[140]. The diffusion coefficients of all phospholipids laterally on the membrane surface were estimated with the *gmx msd* command after unwrapping and skipping the first and last μs of the trajectories.

Supplementary Methods L) Protein-lipid Interactions

<u>Transmembrane proteins</u>

Kir2.2. The protein structure (PDB ID 6M84)[69] was downloaded from the OPM database[141] and processed with the Chimera Dock Prep tool[142] to add any missing

atoms. The CG model of the protein was generated using *martinize2*[143], version 0.9.6. For the elastic network, the four chains of the tetramer were merged, and we used a force constant of 700 kJ mol^{-1} nm^{-2} with an elastic bond upper cutoff of 0.8 nm. The termini were set neutral and side chain corrections (scFix option) were applied. Using *insane*[13], the protein was embedded in a lipid bilayer consisting of 100% POPC lipids in the upper leaflet, and 95% POPC and 5% PI(4,5)P_2 (POP6) lipids in the lower leaflet; water and 0.15M NaCl were also added.

ADP/ATP carrier. The protein structure (PDB ID 1OKC)[71] was prepared as described above for Kir2.2. For Martini 3, the simulation systems were prepared with a lipid bilayer consisting of 45% POPC, 35% POPE, and (i) 20% cardiolipins with PO tails (POCL) or (ii) 20% tetra-linoleoyl cardiolipins (TLCL). Martini 2 CG system: A Martini 2 system was prepared using the python3 version of the martinize.py script, version 2.6_3, available from the Martini website. The elastic network was applied with the same upper bond cutoff and force constant as for the Martini 3 systems, using the elnedyn22 option[144]. The POCL lipid mixture and water and ions were added with *insane*[13].

Peripheral membrane proteins.

For BtPI-PLC (PDB ID 3EA1/2OR2), SaPI-PLC (PDB ID 4F2T), and PH domain (PDB ID 1MAI), the proteins were converted to CG representations with an elastic network force constant of 700 kJ mol^{-1} nm^{-2} and with an elastic bond upper cut-off of 0.8 nm. Side chain corrections were applied using the scFix option. These CG structures were placed in a box of dimension 8.96 x 8.96 x 15 nm^3 using *insane*[13] to build the corresponding bilayers. These resulted in 288 lipids for single component lipids (DMPC/POPC/POPG) and 144 lipids in each leaflet for BtPI-PLC and SaPI-PLC systems.

For the PH domain, two bilayer compositions were prepared. The four components bilayer had POPC:POPS:PIP_2:PIP_3 in a 73:20:5:2 ratio and two components bilayer had POPC:PIP_2 in a 95:5 ratio. The four-component bilayer had 284 lipids (142 in each leaflet), and the two-component bilayer had 286 lipids (143 in each leaflet). We used counter ions (Na^+ or Cl^-) for the single components systems (PI-PLCs) and 150 mM NaCl for the multi-component systems (PH domain). During the placement of the

proteins in the box, a center of mass distance of 6 nm was maintained between the proteins and the bilayers.

The FGF2 protein was placed 2 nm away from the membrane (consisting of 5 mol% $PI(4,5)P_2$ in a POPC background) in 10 different orientations, and each replica was simulated for 10 µs. We coarse-grained the atomistic model from previous CHARMM36m simulations as a starting point. We used two reference models: FGF2 after a 1-µs relaxation in water and the FGF2 model bound to the membrane via its binding pocket.

The VP40 dimer initial configurations were generated following the same protocol using two reference models: VP40 after a 1-µs relaxation in water and the VP40 model bound to the membrane via K224, K225, K274, and K275. The side chain correction (scFix) was applied for all systems.

Simulation Setup

Transmembrane proteins. Using GROMACS 2024.1[109], the Martini 3 systems were energy minimized using the steepest descent algorithm, setting the convergence criteria to 100,000 steps and a maximum force of 0.1 kJ mol^{-1} nm^{-1}. Position restraints were applied to the backbone beads of the protein (with a force constant of 1000 kJ mol^{-1} nm^{-2}). A short NPT 5 ns-long equilibration was performed with the same position restraints. For Martini 3, we performed a production run of 30 ms for Kir2.2, and two production runs of 30 µs each for the ADP/ATP carrier at 310 K with the standard Martini 3 parameters. The Martini 2 system was prepared following the same protocol and simulated for 30 µs-long with GROMACS 2023.2, using parameters from de Jong et al.[145].

Peripheral membrane proteins. The peripheral protein-membrane systems were energy minimized using the steepest descent algorithm followed by 1 ns equilibration in NPT ensemble with a semi-isotropic pressure coupling. The production simulations were carried out at 310 K and with semi-isotropic pressure coupling. No position restraints were applied to the protein.

Simulations Analysis

Minimum distance analysis for transmembrane proteins. For a given lipid species of interest, we tracked as a function of time if each lipid molecule satisfies a distance cutoff of 5 Å from any protein residues and from selected protein residues that define binding sites: For Kir2.2, in each monomer the minimum distance was computed for residues R78, W79 and K188; for the ADP/ATP carrier, we used residues 272-274, residues 72-74 and residue 176 for CDL sites 801, 800 and 802, respectively. The analysis was performed using the Distance analysis module from MDAnalysis[146].

Fractional occupancy. Fractional occupancy for selected lipid species was calculated using the VolMap Tool in VMD[147], setting the resolution to 2 Å. The entire length of each trajectory was used to derive the occupancy maps. Prior to the calculation, the trajectory was processed with the GROMACS *gmx trjconv* tool, using progressive fitting on the backbone beads of the protein.

Minimum distance analysis for peripheral proteins. To quantify membrane-binding events for BtPI-PLC, SaPI-PLC, and the PH domain, the minimum distance between the peripheral proteins and the membrane was calculated using the *gmx mindist* tool. To evaluate the correct binding mode, the minimum distance between selected membrane binding residues with the membrane was calculated.

Contact occupancy for the FGF2 and VP40 systems. Contact occupancy was measured by counting the number of contacts, where a contact is defined as having any bead of the protein within less than 0.6 nm from any atom of the membrane. These contact counts were converted into binary results (0 for no contact, 1 for contact). Frames, where the protein was in contact with the membrane, were used to calculate the fraction of frames where the protein was in contact with the experimentally known binding pockets. The average was calculated over 10 repeats for the entire 10 μs of simulation, and the error bars represent the standard error with $n = 10$.

Molecular representations. All figures representing simulation systems were prepared with VMD[147].

Supplementary Methods M) Quantifying Bilayer Phase Behavior

Simulated Annealing

Four systems were built consisting of pure DPPC (16:0/16:0), DSPC (18:0/18:0), PSM (d18:1/16:0), SSM (d18:1/18:0) using *insane*[13]. Each system was prepared in a hexagonal periodic box (box-vectors ≈ v1(x) = 9.0, v2(y) = 7.8 nm, v3(z) = 9.0 nm, v2(x) = 4.5 nm, v1(y) = v1(z) = v2(z) = v3(x) = v3(y) = 0 nm), to avoid packing artifacts arising from periodic boundary conditions in the gel state. The systems were solvated with regular water beads and 0.15 M NaCl was added. The bilayer was minimized and equilibrated for 50 ns at 373 K and 1 bar. The annealing simulation was initialized from the equilibrated structure and a quenching cycle going from 373 to 273 K at a rate of 1 K/10 ns was performed followed by a heating cycle from 273 to 373 K. The full cycle was 2 μs in total. 5 repeat simulations of the full cycle were performed, starting from the equilibrated structure each time and resampling velocities at 373 K. Frames were saved every 100 ps. Temperature and pressure were maintained using the v-rescale[137] thermostat (τ_t = 1 ps) and the Parrinello-Rahman[117] barostat (τ_p = 12 ps) using semi-isotropic pressure coupling for both equilibration and annealing simulations. Simulations were performed using GROMACS[109] v2024.0.

Seeding Simulations

The seeding setup for evaluation of the bilayer transition temperature was prepared in the following way. Two membranes consisting of 200 lipids were created using COBY[148] (Coarse-grained System Builder). The two systems were then minimized and equilibrated for 50 ns at 30 K above or below the known experimental transition of the given lipid type, to obtain the APL of the liquid and gel phases, respectively. Utilizing the found APL, COBY was then used to create a membrane consisting of 200 lipids in a liquid phase surrounding a gel patch also consisting of 200 lipids (the gel patch was inserted into the liquid bilayer). The system was minimized and equilibrated for 10 ns with separate temperature baths for the gel patch, simulated at 30 K below the experimental transition, while the surrounding liquid membrane was simulated at 30 K above the experimental transition temperature. Three independent repeats of the equilibration were performed with resampled velocities, resulting in three independent

starting structures for the seeds simulations. The seed systems were then each simulated for 500 ns using a constant temperature bath for the entire simulation box, at temperatures ranging from 15 K below the experimental transition temperature to 15 K above, with steps of 2.5 K. The c-rescale barostat (τ_p = 4 ps) was used for equilibrations of the initial membranes where APLs and the gel patch were obtained as well as the final temperature range simulations. The Berendsen[121] barostat (τ_p = 4 ps) was used for equilibrations of the phase-separated systems since c-rescale cannot be used with separate temperature baths. The v-rescale[137] thermostat (τ_t = 1 ps) was used for all simulations. Simulations were performed using GROMACS v2024.0. The Lindemann Index[149] was calculated using a block of the last 100 ns of each simulation and averaged across the three repeats.

Ion-Induced Phosphatidylserine Phase Change

The bilayer systems used to investigate ion-induced phosphatidylserine phase change were systematically constructed using COBY[148]. Pure POPS systems (100% POPS) were prepared in simulation boxes measuring 12 × 12 × 22 nm, while mixed systems (50:50 POPC:POPS) were prepared in boxes of 20 × 20 × 22 nm. For systems containing calcium, a 1.5 Ca^{2+} to PS ratio was used.

Prior to production, the systems underwent 5000 energy minimization steps, followed by equilibration for 10 ns using a 10 fs time step. The production runs for each system were carried out for 10 µs. Throughout the simulations, the temperatures of the lipids and solvent (including Ca^{2+}) were independently maintained at 300 K using the v-rescale[137] thermostat (τ_t = 1 ps), and the pressure was kept at 1 bar using the c-rescale[126] barostat (τ_p = 4 ps) with semi-isotropic pressure coupling applied during both equilibration and production phases[126]. Compressibility was set to 3×10^{-4} bar^{-1}. As suggested by Kim et al.[138], the cut-off distance for the short-range neighbor list was set to 1.35 nm for proper neighbor list updates.

Membrane density profiles were calculated by determining the target particle's distance to the center of the nearby bilayer and histogramming the z-distance component. The center of the nearby bilayer was defined as the center of geometry of all phosphate (PO4) particles. Lipid neighbor enrichment was calculated with LiPyphilic's Neighbours module[64].

Phase Separation in Three-Component Bilayers

The three-component bilayers to investigate phase separation were systematically constructed using *insane*[13] in a 40 × 40 × 10 nm simulation box and solvated with water and 0.15 M NaCl. The resulting systems consisted of ~6000 lipids and ~130,000 total particles.

Before production runs, the systems underwent 1500 steps of energy minimization and were subsequently equilibrated for a total of ~2 ns with increasing time steps (from 1 fs to 20 fs, see Table S7). The production run for each system was 10 μs. During the equilibration and production runs, LINCS[120] constraints were used for ring systems and stiff bond constraints; the LINCS order and iteration were set to 12 and 2. The temperatures of lipids and solvent (NA, CL, and W beads) were independently maintained at 297 K with a time constant for coupling (τ_T) of 1.0 ps, using the Berendsen[121] and v-rescale[137] thermostats during the equilibration and production, respectively. The pressure was maintained at 1 bar using the Berendsen[121] barostat during equilibrations and the Parrinello-Rahman[117] barostat during the production runs. The compressibility was set to 3×10^{-4} bar^{-1}. As suggested by Kim et al.[138], the cut-off distance for the short-range neighbor list was set to 1.35 nm for proper neighbor list updates. To maintain a flat bilayer[133], z-coordinate positional restraints were applied to the PO4 beads of the phospholipids in the upper leaflet, with a force constant of 2 kJ/mol/nm^2.

When determining which phases we present within a single ternary mixture simulation, we found it difficult to correctly extract the correct information when using 'bulk' properties measured for each lipid species (DPPC or DOPC). This could partly be due to significant fractions of a lipid species being in multiple different phases (for instance, both L_b and L_d). Thus, the bulk property of the lipid species would report an average/amalgamation of the features of all the phases present, proving unfeasible to disentangle the signal and elucidate which phases were present.

As such, we instead measured the properties of each individual lipid. In this case, from the radial distribution function (RDF) we extracted a measure of the density of the number of lipids within the first shell of each individual lipid. Specifically, the non-

normalized RDF was measured for the second tail beads for each tail (C2A, C2B, D2A, D2B) and the R1 bead for cholesterol relative to the second tail bead of each lipid. This RDF was averaged over the final 1 μs of simulation.

Using simulation systems with clear, easily identifiable phases present, thresholds were identified to determine the phase of each lipid. If the peak height was *below* the *lower* threshold, a lipid was assigned in the L_d phase. Conversely, if the peak height was *above* the *upper* threshold, a lipid was assigned in the L_b phase. If the peak height was *between* the two thresholds, a lipid was assigned in the L_o phase. Using the information from this analysis, the properties and phase assignments for each lipid were plotted on the final positions of the lipid tails and inspected to determine which phases were present.

Simulations of Non-lamellar Lipid Phases

The stacked bilayer systems were constructed using *insane*[13] with different hydration levels. The single bilayer configuration was then minimized for 1000 steps using the deepest descent algorithm.

The minimized bilayer was then duplicated in z using GROMACS and minimized again using the same approach as before. The stacked bilayer systems were then relaxed for 15 ns followed by 2 μs production run, with a timestep of respectively 15 and 20 fs. The settings applied during all the steps are according to the recent paper by Kim et al. to avoid neighbor list artifacts[138]. The Berendsen[121] and Parinello-Rahman[117] barostats were applied with an anisotropic pressure coupling during the relaxation and production run, respectively, with τ_p = 1ps and 12 ps and with the compressibility set to $3 \cdot 10^{-4}$ bar^{-1}.

The Berendsen[121] and v-rescale[137] thermostat were used during relaxation and production run, respectively. During both steps τ_T = 1 ps and lipids were coupled separately from the solvent.

The reaction field method was used to treat the electrostatics and the van der Waals interactions were truncated after 11 Å[145].

In order to pre-built 4 channels in a hexagonal box, a single channel was first constructed and then replicated 3 more times, using a combination of the tool TS2CG[3]

to construct a single channel of lipids, with a 10 nm length, and PACKMOL[150] to insert the desired number of water beads and ions into the channel.

GROMACS[109] 2023.3 was used for simulations. The minimization, relaxation and production run settings were as described above for the double bilayer systems. The pre-built channels were simulated for 1 μs and repeated three times.

For the pre-constructed H_{11}-phase systems, the lattice distance D_{Hex} was calculated using an in-house script. First, the four water channels were identified using DBSCAN[151, 152] followed by a cylindering fit to obtain the center coordinates for the lattice distance measure.

The protocol for building and simulating an LNP with an H_{11} phase in the core was done as described in Kjølbye et al.[48].

Cubic phases were self-assembled from random starting configurations of fixed MO/water ratios. The required number of MO and water molecules were randomly inserted into a 15 x 15 x 15 nm box and energy was minimized using the steepest descent algorithm for 1000 steps. The system was then equilibrated for 10ns with all nonbonded interactions set to the equivalent of the water self-interaction for the Martini 3 force field, following previously described approaches for correctly optimizing the box volume[63]. After equilibration, the system nonbonded interactions were reset to their default values to allow self-assembly to take place in a 1 μs simulation.

For both equilibration and production simulations, isotropic pressure coupling was used, with the Berendsen[121] barostat used during equilibration, and the Parrinello-Rahman[117] barostat in production. The velocity-rescale thermostat was used for both equilibration and production, with the solvent and the membrane coupled separately.

For temperature annealing simulations, a pre-assembled cubic phase was simulated with an annealed temperature for 10 μs. The temperature increased in 10 K intervals from 298.15 to 368.15 K, increased over 666 ns and held constant for the same interval again. Anisotropic pressure coupling was used with the Parrinello-Rahman[117] barostat with the time constant set to 12 ps and the compressibility set to $3 \cdot 10^{-4}$ bar^{-1}. The velocity-rescale thermostat was used with a time constant of 1 ps.

Supplementary Data N) Martini Database and API

The latest Martini Database[153] (MAD) web service provides access to all new lipid models featured in this publication. Through the MAD RESTful API, users can retrieve the complete list of lipids: http://mad.ens-lyon.fr/api/molecule/list?force_fields=martini3001_NLT&limit=999

Alternatively, the individual can be downloaded as molecule bundles. Each bundle includes the ITP file from this publication, along with coordinate files in both GRO and PDB formats. Here is an example for PYPS: http://mad.ens-lyon.fr/api/molecule/get/alias/PYPS?force_field=martini3001_NLT&version=1.0.

Additionally, MAD integrates the full set of lipid models with two widely used Martini tools: Insane and Polyply. Using MAD:Insane, any lipid can be employed to build membranes with Insane. With MAD:Polyply, the lipid models can be used to generate polymers. Finally, the bonded parameter file used in this publication can be directly downloaded from the Force Fields section of the MAD website: https://mad.ens-lyon.fr/force_fields.

References

1. Risselada, H.J. & Marrink, S.J. Curvature effects on lipid packing and dynamics in liposomes revealed by coarse grained molecular dynamics simulations. *Physical Chemistry Chemical Physics* **11**, 2056--2067 (2009).
2. Marrink, S.J. & Mark, A.E. Molecular dynamics simulation of the formation, structure, and dynamics of small phospholipid vesicles. *Journal of the American Chemical Society* **125**, 15233--15242 (2003).
3. Pezeshkian, W., König, M., Wassenaar, T.A. & Marrink, S.J. Backmapping triangulated surfaces to coarse-grained membrane models. *Nature Communications* **11**, 2296 (2020).
4. Stevens, J.A. et al. Molecular dynamics simulation of an entire cell. *Frontiers in Chemistry* **11**, 1106495 (2023).
5. Doktorova, M., Harries, D. & Khelashvili, G. Determination of bending rigidity and tilt modulus of lipid membranes from real-space fluctuation analysis of molecular dynamics simulations. *Physical Chemistry Chemical Physics* **19**, 16806--16818 (2017).
6. Noguchi, H. Anisotropic surface tension of buckled fluid membranes. *Physical Review E—Statistical, Nonlinear, and Soft Matter Physics* **83**, 061919 (2011).
7. Hu, M., Diggins, P. & Deserno, M. Determining the bending modulus of a lipid membrane by simulating buckling. *The Journal of Chemical Physics* **138** (2013).
8. Eid, J., Razmazma, H., Jraij, A., Ebrahimi, A. & Monticelli, L. On calculating the bending modulus of lipid bilayer membranes from buckling simulations. *The Journal of Physical Chemistry B* **124**, 6299--6311 (2020).
9. Drabik, D., Chodaczek, G., Kraszewski, S. & Langner, M. Mechanical properties determination of DMPC, DPPC, DSPC, and HSPC solid-ordered bilayers. *Langmuir* **36**, 3826--3835 (2020).
10. Fernandez-Puente, L., Bivas, I., Mitov, M.D. & Mlard, P. Temperature and chain length effects on bending elasticity of phosphatidylcholine bilayers. *Europhysics Letters* **28**, 181 (1994).
11. Bochicchio, D. & Monticelli, L. The membrane bending modulus in experiments and simulations: a puzzling picture. *Advances in Biomembranes and Lipid Self-Assembly* **23**, 117--143 (2016).
12. Karal, M.A.S., Billah, M.M., Ahmed, M. & Ahamed, M.K. A review on the measurement of bending rigidity of lipid membranes. *Soft Matter* (2023).
13. Wassenaar, T.A., Ingólfsson, H.I., Bockmann, R.A., Tieleman, D.P. & Marrink, S.J. Computational lipidomics with insane: a versatile tool for generating custom membranes for molecular simulations. *Journal of Chemical Theory and Computation* **11**, 2144--2155 (2015).
14. Marrink, S.J., Risselada, H.J., Yefimov, S., Tieleman, D.P. & de Vries, A.H. The MARTINI force field: coarse grained model for biomolecular simulations. *The Journal of Physical Chemistry B* **111**, 7812--7824 (2007).
15. Marrink, S.J., Risselada, J. & Mark, A.E. Simulation of gel phase formation and melting in lipid bilayers using a coarse grained model. *Chemistry and Physics of Lipids* **135**, 223--244 (2005).
16. Venable, R.M. et al. Lipid and Peptide Diffusion in Bilayers: The Saffman--Delbrück Model and Periodic Boundary Conditions. *The Journal of Physical Chemistry B* **121**, 3443--3457 (2017).
17. Irving, J.H. & Kirkwood, J.G. The statistical mechanical theory of transport processes. IV. The equations of hydrodynamics. *The Journal of Chemical Physics* **18**, 817--829 (1950).
18. Campomanes, P., Prabhu, J., Zoni, V. & Vanni, S. Recharging your fats: CHARMM36 parameters for neutral lipids triacylglycerol and diacylglycerol. *Biophysical Reports* **1** (2021).
19. Tarafdar, P.K., Chakraborty, H., Dennison, S.M. & Lentz, B.R. Phosphatidylserine inhibits and calcium promotes model membrane fusion. *Biophysical Journal* **103**, 1880--1889 (2012).
20. Mirza, M., Guo, Y., Arnold, K., Oss, C.J.v. & Ohki, S. Hydrophobizing effect of cations on acidic phospholipid membranes. *Journal of Dispersion Science and Technology* **19**, 951--962 (1998).
21. Ohnishi, S. & Ito, T. Calcium-induced phase separations in phosphatidylserine-phosphatidylcholine membranes. *Biochemistry* **13**, 881--887 (1974).
22. Casal, H.L., Martin, A., Mantsch, H.H., Paltauf, F. & Hauser, H. Infrared studies of fully hydrated unsaturated phosphatidylserine bilayers. Effect of lithium and calcium. *Biochemistry* **26**, 7395--7401 (1987).
23. Sarmento, M.J., Coutinho, A., Fedorov, A., Prieto, M. & Fernandes, F. Ca2+ induces PI (4, 5) P2 clusters on lipid bilayers at physiological PI (4, 5) P2 and Ca2+ concentrations. *Biochimica et Biophysica Acta (BBA)-Biomembranes* **1838**, 822--830 (2014).

24. Borges-Araújo, L. et al. Acyl-chain saturation regulates the order of phosphatidylinositol 4, 5-bisphosphate nanodomains. *Communications Chemistry* **4**, 164 (2021).
25. Macdonald, P.M. & Seelig, J. Calcium binding to mixed phosphatidylglycerol-phosphatidylcholine bilayers as studied by deuterium nuclear magnetic resonance. *Biochemistry* **26**, 1231--1240 (1987).
26. Picas, L. et al. Calcium-induced formation of subdomains in phosphatidylethanolamine-phosphatidylglycerol bilayers: a combined DSC, 31P NMR, and AFM study. *The Journal of Physical Chemistry B* **113**, 4648--4655 (2009).
27. Harlos, K. & Eibl, H. Influence of calcium on phosphatidylglycerol. Two separate lamellar structures. *Biochemistry* **19**, 895--899 (1980).
28. Ito, T. & Ohnishi, S.-I. Ca2+-induced lateral phase separations in phosphatidic acid-phosphatidylcholine membranes. *Biochimica et Biophysica Acta (BBA)-Biomembranes* **352**, 29--37 (1974).
29. Melcrov The complex nature of calcium cation interactions with phospholipid bilayers. *Scientific Reports* **6**, 38035 (2016).
30. Dluhy, R., Cameron, D.G., Mantsch, H.H. & Mendelsohn, R. Fourier transform infrared spectroscopic studies of the effect of calcium ions on phosphatidylserine. *Biochemistry* **22**, 6318--6325 (1983).
31. Roux, M. & Bloom, M. Ca2 Li+, Na+, and K distributions in the headgroup region of binary membranes of phosphatidylcholine and phosphatidylserine as seen by deuterium NMR. *Biochemistry* **29**, 7077--7089 (1990).
32. Antila, H. et al. Headgroup structure and cation binding in phosphatidylserine lipid bilayers. *The Journal of Physical Chemistry B* **123**, 9066--9079 (2019).
33. Roux, M. & Bloom, M. Calcium binding by phosphatidylserine headgroups. Deuterium NMR study. *Biophysical Journal* **60**, 38--44 (1991).
34. Feigenson, G.W. On the nature of calcium ion binding between phosphatidylserine lamellae. *Biochemistry* **25**, 5819--5825 (1986).
35. Silvius, J.R. Calcium-induced lipid phase separations and interactions of phosphatidylcholine/anionic phospholipid vesicles. Fluorescence studies using carbazole-labeled and brominated phospholipids. *Biochemistry* **29**, 2930--2938 (1990).
36. van Meer, G., Voelker, D.R. & Feigenson, G.W. Membrane lipids: where they are and how they behave. *Nature Reviews Molecular Cell Biology* **9**, 112--124 (2008).
37. Phase Transitions and Phase Behavior of Lipids , bookTitle= Encyclopedia of Biophysics. (Springer Berlin Heidelberg, Berlin, Heidelberg; 2013).
38. Jouhet, J. Importance of the hexagonal lipid phase in biological membrane organization. *Frontiers in Plant Science* **4**, 494 (2013).
39. Chernomordik, L.V. & Kozlov, M.M. Mechanics of membrane fusion. *Nature Structural \& Molecular Biology* **15**, 675--683 (2008).
40. Yang, L. & Huang, H.W. Observation of a membrane fusion intermediate structure. *Science* **297**, 1877--1879 (2002).
41. Yu, H. et al. Inverse cubic and hexagonal mesophase evolution within ionizable lipid nanoparticles correlates with mRNA transfection in macrophages. *Journal of the American Chemical Society* **145**, 24765--24774 (2023).
42. Kjølbye, L.R. et al. Towards design of drugs and delivery systems with the Martini coarse-grained model. *QRB Discovery* **3**, e19 (2022).
43. Sivadasan, D., Sultan, M.H., Alqahtani, S.S. & Javed, S. Cubosomes in drug delivery—a comprehensive review on its structural components, preparation techniques and therapeutic applications. *Biomedicines* **11**, 1114 (2023).
44. Yaghmur, A. in Lipid-based Nanostructures for Food Encapsulation Purposes 483--522 (2019).
45. Tan, C., Hosseini, S.F. & Jafari, S.M. Cubosomes and hexosomes as novel nanocarriers for bioactive compounds. *Journal of Agricultural and Food Chemistry* **70**, 1423--1437 (2022).
46. Oliveira, C., Coelho, C., Teixeira, J.A., Ferreira-Santos, P. & Botelho, C.M. Nanocarriers as active ingredients enhancers in the cosmetic industry—The European and North America regulation challenges. *Molecules* **27**, 1669 (2022).
47. Vinardell, M.P. & Mitjans, M. Nanocarriers for delivery of antioxidants on the skin. *Cosmetics* **2**, 342--354 (2015).
48. Kjølbye, L.R., Valrio, M. & Paloncov Martini 3 building blocks for Lipid Nanoparticle design. (2024).
49. Dully, M. et al. Modulating the release of pharmaceuticals from lipid cubic phases using a lipase inhibitor. *Journal of Colloid and Interface Science* **573**, 176--192 (2020).

50. Barriga, H.M.G., Holme, M.N. & Stevens, M.M. Cubosomes: the next generation of smart lipid nanoparticles? *Angewandte Chemie International Edition* **58**, 2958--2978 (2019).
51. Philipp, J., Dabkowska, A., Reiser, A., Frank, K. & Krzyszto pH-dependent structural transitions in cationic ionizable lipid mesophases are critical for lipid nanoparticle function. *Proceedings of the National Academy of Sciences* **120**, e2310491120 (2023).
52. Iscaro, J. et al. Lyotropic Liquid Crystalline Phase Nanostructure and Cholesterol Enhance Lipid Nanoparticle Mediated mRNA Transfection in Macrophages. *Advanced Functional Materials*, 2405286 (2024).
53. Rand, R.P. & Fuller, N.L. Structural dimensions and their changes in a reentrant hexagonal-lamellar transition of phospholipids. *Biophysical Journal* **66**, 2127--2138 (1994).
54. Yang, L., Ding, L. & Huang, H.W. New phases of phospholipids and implications to the membrane fusion problem. *Biochemistry* **42**, 6631--6635 (2003).
55. Ramezanpour, M. et al. Structural properties of inverted hexagonal phase: A hybrid computational and experimental approach. *Langmuir* **36**, 6668--6680 (2020).
56. Marrink, S.-J. & Mark, A.E. Molecular view of hexagonal phase formation in phospholipid membranes. *Biophysical Journal* **87**, 3894--3900 (2004).
57. Kulkarni, C.V., Wachter, W., Iglesias-Salto, G., Engelskirchen, S. & Ahualli, S. Monoolein: a magic lipid? *Physical Chemistry Chemical Physics* **13**, 3004--3021 (2011).
58. Qiu, H. & Caffrey, M. The phase diagram of the monoolein/water system: metastability and equilibrium aspects. *Biomaterials* **21**, 223--234 (2000).
59. Kulkarni, C.V. et al. Engineering bicontinuous cubic structures at the nanoscale—the role of chain splay. *Soft Matter* **6**, 3191--3194 (2010).
60. Harper, P.E., Mannock, D.A., Lewis, R.N.A.H., McElhaney, R.N. & Gruner, S.M. X-ray diffraction structures of some phosphatidylethanolamine lamellar and inverted hexagonal phases. *Biophysical Journal* **81**, 2693--2706 (2001).
61. Schwarz, U.S. & Gompper, G. Systematic approach to bicontinuous cubic phases in ternary amphiphilic systems. *Physical Review E* **59**, 5528 (1999).
62. Khelashvili, G. et al. Why GPCRs behave differently in cubic and lamellar lipidic mesophases. *Journal of the American Chemical Society* **134**, 15858--15868 (2012).
63. Fuhrmans, M., Knecht, V. & Marrink, S.J. A single bicontinuous cubic phase induced by fusion peptides. *Journal of the American Chemical Society* **131**, 9166--9167 (2009).
64. Smith, P. & Lorenz, C.D. LiPyphilic: A Python toolkit for the analysis of lipid membrane simulations. *Journal of Chemical Theory and Computation* **17**, 5907--5919 (2021).
65. Ingólfsson, H.I. et al. Capturing biologically complex tissue-specific membranes at different levels of compositional complexity. *The Journal of Physical Chemistry B* **124**, 7819--7829 (2020).
66. Monticelli, L. et al. The MARTINI coarse-grained force field: extension to proteins. *Journal of Chemical Theory and Computation* **4**, 819--834 (2008).
67. Periole, X., Knepp, A.M., Sakmar, T.P., Marrink, S.J. & Huber, T. Structural determinants of the supramolecular organization of G protein-coupled receptors in bilayers. *Journal of the American Chemical Society* **134**, 10959--10965 (2012).
68. Zeppelin, T., Pedersen, K.B., Berglund, N.A., Periole, X. & Schioett, B. Effect of palmitoylation on the dimer formation of the human dopamine transporter. *Scientific Reports* **11**, 4164 (2021).
69. Zangerl-Plessl, E.-M. et al. Atomistic basis of opening and conduction in mammalian inward rectifier potassium (Kir2. 2) channels. *Journal of General Physiology* **152**, e201912422 (2019).
70. Borges-Araújo, L., Souza, P.C.T., Fernandes, F. & Melo, M.N. Improved parameterization of phosphatidylinositide lipid headgroups for the martini 3 coarse-grain force field. *Journal of Chemical Theory and Computation* **18**, 357--373 (2021).
71. Pebay-Peyroula, E. et al. Structure of mitochondrial ADP/ATP carrier in complex with carboxyatractyloside. *Nature* **426**, 39--44 (2003).
72. Duncan, A.L., Ruprecht, J.J., Kunji, E.R.S. & Robinson, A.J. Cardiolipin dynamics and binding to conserved residues in the mitochondrial ADP/ATP carrier. *Biochimica et Biophysica Acta (BBA)-Biomembranes* **1860**, 1035--1045 (2018).
73. Javanainen, M., Martinez-Seara, H. & Vattulainen, I. Excessive aggregation of membrane proteins in the Martini model. *PloS One* **12**, e0187936 (2017).
74. Alessandri, R. et al. Pitfalls of the Martini model. *Journal of Chemical Theory and Computation* **15**, 5448--5460 (2019).
75. Grauffel, C.d. et al. Cation-pi interactions as lipid-specific anchors for phosphatidylinositol-specific phospholipase C. *Journal of the American Chemical Society* **135**, 5740--5750 (2013).

76. Khan, H.M. et al. Improving the force field description of tyrosine--choline cation-pi interactions: QM investigation of phenol--N (Me) 4+ interactions. *Journal of Chemical Theory and Computation* **12**, 5585--5595 (2016).
77. Roberts, M.F., Khan, H.M., Goldstein, R., Reuter, N. & Gershenson, A. Search and subvert: minimalist bacterial phosphatidylinositol-specific phospholipase C enzymes. *Chemical Reviews* **118**, 8435--8473 (2018).
78. Yang, B. et al. Quantifying transient interactions between Bacillus phosphatidylinositol-specific phospholipase-C and phosphatidylcholine-rich vesicles. *Journal of the American Chemical Society* **137**, 14--17 (2015).
79. Cheng, J., Goldstein, R., Stec, B., Gershenson, A. & Roberts, M.F. Competition between anion binding and dimerization modulates Staphylococcus aureus phosphatidylinositol-specific phospholipase C enzymatic activity. *Journal of Biological Chemistry* **287**, 40317--40327 (2012).
80. Khan, H.M. et al. Capturing choline--aromatics cation-pi interactions in the MARTINI force field. *Journal of Chemical Theory and Computation* **16**, 2550--2560 (2020).
81. Cho, W. & Stahelin, R.V. Membrane-protein interactions in cell signaling and membrane trafficking. *Annu. Rev. Biophys. Biomol. Struct.* **34**, 119--151 (2005).
82. Ferguson, K.M., Lemmon, M.A., Schlessinger, J. & Sigler, P.B. Structure of the high affinity complex of inositol trisphosphate with a phospholipase C pleckstrin homology domain. *Cell* **83**, 1037--1046 (1995).
83. Yamamoto, E., Kalli, A.C., Yasuoka, K. & Sansom, M.S.P. Interactions of pleckstrin homology domains with membranes: adding back the bilayer via high-throughput molecular dynamics. *Structure* **24**, 1421--1431 (2016).
84. Nickel, W. & Seedorf, M. Unconventional mechanisms of protein transport to the cell surface of eukaryotic cells. *Annual Review of Cell and Developmental Biology* **24**, 287--308 (2008).
85. Sparn, C., Meyer, A., Saleppico, R. & Nickel, W. Unconventional secretion mediated by direct protein self-translocation across the plasma membranes of mammalian cells. *Trends in Biochemical Sciences* **47**, 699--709 (2022).
86. Lolicato, F. & Nickel, W. A role for liquid-ordered plasma membrane nanodomains coordinating the unconventional secretory pathway of fibroblast growth factor 2? *Frontiers in Cell and Developmental Biology* **10**, 864257 (2022).
87. Feldmann, H. & Klenk, H.-D. Marburg and Ebola viruses. *Advances in Virus Research* **47**, 1--52 (1996).
88. Geisbert, T.W. & Jahrling, P.B. Differentiation of filoviruses by electron microscopy. *Virus Research* **39**, 129--150 (1995).
89. Khan, H.M. et al. A role for weak electrostatic interactions in peripheral membrane protein binding. *Biophysical Journal* **110**, 1367--1378 (2016).
90. Cheng, J., Goldstein, R., Gershenson, A., Stec, B. & Roberts, M.F. The cation-π box is a specific phosphatidylcholine membrane targeting motif. *Journal of Biological Chemistry* **288**, 14863--14873 (2013).
91. Müller, H.-M. et al. Formation of disulfide bridges drives oligomerization, membrane pore formation, and translocation of fibroblast growth factor 2 to cell surfaces. *Journal of Biological Chemistry* **290**, 8925--8937 (2015).
92. Steringer, J.P. & Nickel, W. The molecular mechanism underlying unconventional secretion of Fibroblast Growth Factor 2 from tumour cells. *Biology of the Cell* **109**, 375--380 (2017).
93. Steringer, J.P. et al. Key steps in unconventional secretion of fibroblast growth factor 2 reconstituted with purified components. *eLife* **6**, e28985 (2017).
94. Temmerman, K. et al. A direct role for phosphatidylinositol-4, 5-bisphosphate in unconventional secretion of fibroblast growth factor 2. *Traffic* **9**, 1204--1217 (2008).
95. Lolicato, F. et al. Disulfide bridge-dependent dimerization triggers FGF2 membrane translocation into the extracellular space. *eLife* **12**, RP88579 (2024).
96. Lolicato, F. et al. Cholesterol promotes clustering of PI (4, 5) P2 driving unconventional secretion of FGF2. *Journal of Cell Biology* **221**, e202106123 (2022).
97. Winter, S.L. et al. The Ebola virus VP40 matrix layer undergoes endosomal disassembly essential for membrane fusion. *The EMBO Journal* **42**, e113578 (2023).
98. Thomasen, F.E. et al. Rescaling protein-protein interactions improves Martini 3 for flexible proteins in solution. *Nature Communications* **15**, 6645 (2024).
99. Soni, J., Gupta, S. & Mandal, T. Recalibration of MARTINI-3 Parameters for Improved Interactions between Peripheral Proteins and Lipid Bilayers. *Journal of Chemical Theory and Computation* (2024).

100. Jarin, Z., Venable, R.M., Han, K. & Pastor, R.W. Ion-Induced PIP2 Clustering with Martini3: Modification of Phosphate--Ion Interactions and Comparison with CHARMM36. *The Journal of Physical Chemistry B* **128**, 2134--2143 (2024).
101. Grünewald, F. et al. Martini 3 coarse-grained force field for carbohydrates. *Journal of Chemical Theory and Computation* **18**, 7555--7569 (2022).
102. Pereira, G.P. et al. Bartender: Martini 3 Bonded Terms via Quantum Mechanics-Based Molecular Dynamics. *Journal of Chemical Theory and Computation* **20**, 5763--5773 (2024).
103. Stroh, K.S., Souza, P.C.T., Monticelli, L. & Risselada, H.J. CGCompiler: Automated Coarse-Grained Molecule Parametrization via Noise-Resistant Mixed-Variable Optimization. *Journal of Chemical Theory and Computation* **19**, 8384--8400 (2023).
104. Empereur-Mot, C. et al. Automatic optimization of lipid models in the Martini force field using SwarmCG. *Journal of Chemical Information and Modeling* **63**, 3827--3838 (2023).
105. Bennett, W.F.D. & Tieleman, D.P. Water defect and pore formation in atomistic and coarse-grained lipid membranes: pushing the limits of coarse graining. *Journal of Chemical Theory and Computation* **7**, 2981--2988 (2011).
106. Jin, J., Yu, A. & Voth, G.A. Temperature and phase transferable bottom-up coarse-grained models. *Journal of Chemical Theory and Computation* **16**, 6823--6842 (2020).
107. Elahi, A., Bidault, X. & Chaudhuri, S. Temperature-transferable coarse-grained model for poly (propylene oxide) to study thermo-responsive behavior of triblock copolymers. *The Journal of Physical Chemistry B* **126**, 292--307 (2022).
108. Marrink, S.J. & Tieleman, D.P. Perspective on the Martini model. *Chemical Society Reviews* **42**, 6801--6822 (2013).
109. Abraham, M.J. et al. GROMACS: High performance molecular simulations through multi-level parallelism from laptops to supercomputers. *SoftwareX* **1**, 19--25 (2015).
110. Hub, J.S. & Awasthi, N. Probing a continuous polar defect: A reaction coordinate for pore formation in lipid membranes. *Journal of Chemical Theory and Computation* **13**, 2352--2366 (2017).
111. Promoting transparency and reproducibility in enhanced molecular simulations. *Nature Methods* **16**, 670--673 (2019).
112. Vanommeslaeghe, K. et al. CHARMM general force field: A force field for drug-like molecules compatible with the CHARMM all-atom additive biological force fields. *Journal of Computational Chemistry* **31**, 671--690 (2010).
113. Lee, J. et al. CHARMM-GUI input generator for NAMD, GROMACS, AMBER, OpenMM, and CHARMM/OpenMM simulations using the CHARMM36 additive force field. *Biophysical Journal* **110**, 641a (2016).
114. Lee, J. et al. CHARMM-GUI membrane builder for complex biological membrane simulations with glycolipids and lipoglycans. *Journal of Chemical Theory and Computation* **15**, 775--786 (2018).
115. Darden, T., York, D. & Pedersen, L. Particle mesh Ewald: An N log (N) method for Ewald sums in large systems. *The Journal of Chemical Physics* **98**, 10089--10092 (1993).
116. Evans, D.J. & Holian, B.L. The nose--hoover thermostat. *The Journal of Chemical Physics* **83**, 4069--4074 (1985).
117. Parrinello, M. & Rahman, A. Polymorphic transitions in single crystals: A new molecular dynamics method. *Journal of Applied physics* **52**, 7182--7190 (1981).
118. Grossfield, A. (2014).
119. Jo, S., Kim, T., Iyer, V.G. & Im, W. CHARMM-GUI: a web-based graphical user interface for CHARMM. *Journal of Computational Chemistry* **29**, 1859--1865 (2008).
120. Hess, B., Bekker, H., Berendsen, H.J.C. & Fraaije, J.G.E.M. LINCS: a linear constraint solver for molecular simulations. *Journal of Computational Chemistry* **18**, 1463--1472 (1997).
121. Berendsen, H.J.C., Postma, J.P.M.v., Van Gunsteren, W.F., DiNola, A. & Haak, J.R. Molecular dynamics with coupling to an external bath. *The Journal of Chemical Physics* **81**, 3684--3690 (1984).
122. Berendsen, H.J.C., van der Spoel, D. & van Drunen, R. GROMACS: A message-passing parallel molecular dynamics implementation. *Computer Physics Communications* **91**, 43--56 (1995).
123. Klauda, J.B. et al. Update of the CHARMM all-atom additive force field for lipids: validation on six lipid types. *The Journal of Physical Chemistry B* **114**, 7830--7843 (2010).
124. Leonard, A.N., Pastor, R.W. & Klauda, J.B. Parameterization of the charmm all-atom force field for ether lipids and model linear ethers. *The Journal of Physical Chemistry B* **122**, 6744--6754 (2018).

125. West, A. et al. How do ethanolamine plasmalogens contribute to order and structure of neurological membranes? *The Journal of Physical Chemistry B* **124**, 828--839 (2020).
126. Bernetti, M. & Bussi, G. Pressure control using stochastic cell rescaling. *The Journal of Chemical Physics* **153** (2020).
127. Buchoux, S. FATSLiM: a fast and robust software to analyze MD simulations of membranes. *Bioinformatics* **33**, 133--134 (2017).
128. Kučerka, N. et al. Lipid bilayer structure determined by the simultaneous analysis of neutron and X-ray scattering data. *Biophysical journal* **95**, 2356--2367 (2008).
129. Kučerka, N., Nieh, M.-P. & Katsaras, J. Fluid phase lipid areas and bilayer thicknesses of commonly used phosphatidylcholines as a function of temperature. *Biochimica et Biophysica Acta (BBA)-Biomembranes* **1808**, 2761--2771 (2011).
130. Tristram-Nagle, S., Petrache, H.I. & Nagle, J.F. Structure and interactions of fully hydrated dioleoylphosphatidylcholine bilayers. *Biophysical Journal* **75**, 917--925 (1998).
131. Khelashvili, G., Kollmitzer, B., Heftberger, P., Pabst, G. & Harries, D. Calculating the bending modulus for multicomponent lipid membranes in different thermodynamic phases. *Journal of Chemical Theory and Computation* **9**, 3866--3871 (2013).
132. Johner, N., Harries, D. & Khelashvili, G. Implementation of a methodology for determining elastic properties of lipid assemblies from molecular dynamics simulations. *BMC Bioinformatics* **17**, 1--11 (2016).
133. Ozturk, T.N. et al. Building complex membranes with Martini 3. *Methods in Enzymology* (2024).
134. Bullerjahn, J.T., Von Blow, S. & Hummer, G. Optimal estimates of self-diffusion coefficients from molecular dynamics simulations. *The Journal of Chemical Physics* **153** (2020).
135. von Bülow, S., Bullerjahn, J.T. & Hummer, G. Systematic errors in diffusion coefficients from long-time molecular dynamics simulations at constant pressure. *The Journal of Chemical Physics* **153** (2020).
136. Bullerjahn, J.T., von Bülow, S.r., Heidari, M., Hnin, J. & Hummer, G. Unwrapping NPT simulations to calculate diffusion coefficients. *Journal of Chemical Theory and Computation* **19**, 3406--3417 (2023).
137. Bussi, G., Donadio, D. & Parrinello, M. Canonical sampling through velocity rescaling. *The Journal of Chemical Physics* **126** (2007).
138. Kim, H., Fábián, B. & Hummer, G. Neighbor list artifacts in molecular dynamics simulations. *Journal of Chemical Theory and Computation* **19**, 8919--8929 (2023).
139. Bennett, W.F.D. et al. ezAlign: A Tool for Converting Coarse-Grained Molecular Dynamics Structures to Atomistic Resolution for Multiscale Modeling. *Molecules* **29**, 3557 (2024).
140. Ingólfsson, H.I. et al. Lipid organization of the plasma membrane. *Journal of the American Chemical Society* **136**, 14554--14559 (2014).
141. Lomize, M.A., Pogozheva, I.D., Joo, H., Mosberg, H.I. & Lomize, A.L. OPM database and PPM web server: resources for positioning of proteins in membranes. *Nucleic Acids Research* **40**, D370--D376 (2012).
142. Pettersen, E.F. et al. UCSF Chimera—a visualization system for exploratory research and analysis. *Journal of Computational Chemistry* **25**, 1605--1612 (2004).
143. Kroon, P.C. et al. Martinize2 and vermouth: Unified framework for topology generation. *eLife* (2023).
144. Periole, X., Cavalli, M., Marrink, S.-J. & Ceruso, M.A. Combining an elastic network with a coarse-grained molecular force field: structure, dynamics, and intermolecular recognition. *Journal of Chemical Theory and Computation* **5**, 2531--2543 (2009).
145. de Jong, D.H., Baoukina, S., Ingólfsson, H.I. & Marrink, S.J. Martini straight: Boosting performance using a shorter cutoff and GPUs. *Computer Physics Communications* **199**, 1--7 (2016).
146. Michaud-Agrawal, N., Denning, E.J., Woolf, T.B. & Beckstein, O. MDAnalysis: a toolkit for the analysis of molecular dynamics simulations. *Journal of Computational Chemistry* **32**, 2319--2327 (2011).
147. Humphrey, W., Dalke, A. & Schulten, K. VMD: visual molecular dynamics. *Journal of Molecular Graphics* **14**, 33--38 (1996).
148. Andreasen, M.D.a.S., Paulo C. T. and Schiøtt, Birgit and Zuzic, Lorena Creating Coarse-Grained Systems with COBY: Towards Higher Accuracy in Membrane Complexity. (2024).
149. Lindemann, F.A. The calculation of molecular vibration frequencies. *Phys. Z.* **11**, 609 (1910).
150. Martnez, L., Andrade, R., Birgin, E.G. & Martnez, J.M. PACKMOL: A package for building initial configurations for molecular dynamics simulations. *Journal of Computational Chemistry* **30**, 2157--2164 (2009).

151. Schubert, E., Sander, J., Ester, M., Kriegel, H.P. & Xu, X. DBSCAN revisited, revisited: why and how you should (still) use DBSCAN. *ACM Transactions on Database Systems (TODS)* **42**, 1--21 (2017).
152. Ester, M., Kriegel, H.-P., Sander, J., Xu, X. & et al., Vol. 96 226--231 (1996).
153. Hilpert, C. et al. Facilitating CG simulations with MAD: The MArtini database server. *Journal of chemical information and modeling* **63**, 702--710 (2023).
