# Supplementary material for: The Martini 3 Lipidome: Expanded and Refined Parameters Improve Lipid Phase Behavior
Source: ACS Cent Sci. 2025 Jul 31;11(9):1598–610. doi: 10.1021/acscentsci.5c00755 (PMC12464760; doi:10.1021/acscentsci.5c00755)
Supplement: Supplementary file 2 [file oc5c00755_si_002.zip › M3-Lipidome_Data/Martini_lipid_Benchmark/martini_benchmark.pdf]

MARTINI LIPID BENCHMARK
APL

Ref Name	Lipid Tail	APL [nm^2]	Uncertainty [nm^2]	T [K]	NaCl [M]	Ref
DLPC	12:0/12:0	0.596	0.012	293.15	no	https://doi.org/10.1016/j.bbamem.2011.07.022
DLPC	12:0/12:0	0.608	0.012	303.15	no	https://doi.org/10.1016/j.bbamem.2011.07.022
DLPC	12:0/12:0	0.648	0.013	323.15	no	https://doi.org/10.1016/j.bbamem.2011.07.022
DLPC	12:0/12:0	0.659	0.013	333.15	no	https://doi.org/10.1016/j.bbamem.2011.07.022
DMPC	14:0/14:0	0.599	0.012	303.15	no	https://doi.org/10.1016/j.bbamem.2011.07.022
DMPC	14:0/14:0	0.633	0.013	323.15	no	https://doi.org/10.1016/j.bbamem.2011.07.022
DMPC	14:0/14:0	0.657	0.013	333.15	no	https://doi.org/10.1016/j.bbamem.2011.07.022
DPPC	16:0/16:0	0.631	0.013	323.15	no	https://doi.org/10.1016/j.bbamem.2011.07.022
DPPC	16:0/16:0	0.65	0.013	333.15	no	https://doi.org/10.1016/j.bbamem.2011.07.022
DSPC	18:0/18:0	0.638	0.013	333.15	no	https://doi.org/10.1016/j.bbamem.2011.07.022
MSPC	14:0/18:0	0.622	0.012	323.15	no	https://doi.org/10.3390/sym13081441
SMPC	18:0/14:0	0.62	0.012	323.15	no	https://doi.org/10.3390/sym13081441
PMPC	16:0/14:0	0.629	0.013	323.15	no	https://doi.org/10.3390/sym13081441
DRPC	14:1/14:1	0.642	0.01	303.15	no	https://doi.org/10.1016/j.bpj.2009.06.050
DYPC	16:1/16:1	0.658	0.01	303.15	no	https://doi.org/10.1016/j.bpj.2009.06.050
DOPC	18:1/18:1	0.643	0.013	293.15	no	https://doi.org/10.1039/C6SM02727J
DOPC	18:1/18:1	0.669	0.01	303.15	no	https://doi.org/10.1016/j.bpj.2009.06.050
DGPC	20:1/20:1	0.666	0.01	303.15	no	https://doi.org/10.1016/j.bpj.2009.06.050
DEPC	22:1/22:1	0.657	0.01	303.15	no	https://doi.org/10.1016/j.bpj.2009.06.050
DNPC	24:1/24:1	0.627	0.01	303.15	no	https://doi.org/10.1016/j.bpj.2009.06.050
PSM	d18:1/16:0	0.6	0.012	318.15	no	https://doi.org/10.1021/acs.jpcb.0c03389
PSM	d18:1/16:0	0.619	0.012	328.15	no	https://doi.org/10.1021/acs.jpcb.0c03389
SSM	d18:1/18:0	0.625	0.013	328.15	no	https://doi.org/10.1021/acs.jpcb.0c03389
SSM	d18:1/18:0	0.649	0.013	338.15	no	https://doi.org/10.1021/acs.jpcb.0c03389
POPC	16:0/18:1	0.627	0.013	293.15	no	https://doi.org/10.1016/j.bbamem.2011.07.022
POPC	16:0/18:1	0.643	0.013	303.15	no	https://doi.org/10.1016/j.bbamem.2011.07.022
POPC	16:0/18:1	0.673	0.013	323.15	no	https://doi.org/10.1016/j.bbamem.2011.07.022
POPC	16:0/18:1	0.681	0.014	333.15	no	https://doi.org/10.1016/j.bbamem.2011.07.022
SOPC	18:0/18:1	0.638	0.013	293.15	no	https://doi.org/10.1016/j.bbamem.2011.07.022
SOPC	18:0/18:1	0.655	0.013	303.15	no	https://doi.org/10.1016/j.bbamem.2011.07.022
SOPC	18:0/18:1	0.681	0.013	323.15	no	https://doi.org/10.1016/j.bbamem.2011.07.022
SOPC	18:0/18:1	0.694	0.014	333.15	no	https://doi.org/10.1016/j.bbamem.2011.07.022
PDPC	16:0/22:6	0.693	0.013	293.15	no	https://doi.org/10.1016/j.chemphyslip.2020.104892
PDPC	16:0/22:6	0.711	0.014	303.15	no	https://doi.org/10.1016/j.chemphyslip.2020.104892
PDPC	16:0/22:6	0.729	0.015	313.15	no	https://doi.org/10.1016/j.chemphyslip.2020.104892
SDPC	18:0/22:6	0.704	0.014	303.15	no	https://doi.org/10.1016/j.chemphyslip.2020.104892
DLPE	12:0/12:0	0.517	0.01	308.15	no	https://doi.org/10.1021/jp511159q
DLPE	12:0/12:0	0.539	0.011	318.15	no	https://doi.org/10.1021/jp511159q
DLPE	12:0/12:0	0.559	0.011	328.15	no	https://doi.org/10.1021/jp511159q
POPE	16:0/18:1	0.58	0.012	308.15	no	https://doi.org/10.1021/jp511159q
POPE	16:0/18:1	0.592	0.012	313.15	no	https://doi.org/10.1021/jp511159q
POPE	16:0/18:1	0.613	0.012	323.15	no	https://doi.org/10.1021/jp511159q

SOPE	18:0/18:1	0.568	0.011	308.15	no	https://doi.org/10.1021/jp511159q
SOPE	18:0/18:1	0.578	0.012	313.15	no	https://doi.org/10.1021/jp511159q
SOPE	18:0/18:1	0.601	0.012	323.15	no	https://doi.org/10.1021/jp511159q
DLPG	12:0/12:0	0.602	0.012	293.15	neutralizing	https://doi.org/10.1016/j.bbamem.2014.08.009
DLPG	12:0/12:0	0.621	0.012	303.15	neutralizing	https://doi.org/10.1016/j.bbamem.2014.08.009
DLPG	12:0/12:0	0.653	0.013	323.15	neutralizing	https://doi.org/10.1016/j.bbamem.2014.08.009
DLPG	12:0/12:0	0.671	0.013	333.15	neutralizing	https://doi.org/10.1016/j.bbamem.2014.08.009
DMPG	14:0/14:0	0.625	0.013	303.15	neutralizing	https://doi.org/10.1016/j.bbamem.2014.08.009
DMPG	14:0/14:0	0.66	0.013	323.15	neutralizing	https://doi.org/10.1016/j.bbamem.2014.08.009
DMPG	14:0/14:0	0.675	0.014	333.15	neutralizing	https://doi.org/10.1016/j.bbamem.2014.08.009
DPPG	16:0/16:0	0.647	0.013	323.15	neutralizing	https://doi.org/10.1016/j.bbamem.2014.08.009
DPPG	16:0/16:0	0.668	0.013	333.15	neutralizing	https://doi.org/10.1016/j.bbamem.2014.08.009
DSPG	18:0/18:0	0.668	0.013	333.15	neutralizing	https://doi.org/10.1016/j.bbamem.2014.08.009
POPG	16:0/18:1	0.625	0.013	293.15	neutralizing	https://doi.org/10.1016/j.bbamem.2014.08.009
POPG	16:0/18:1	0.643	0.013	303.15	neutralizing	https://doi.org/10.1016/j.bbamem.2014.08.009
POPG	16:0/18:1	0.684	0.014	323.15	neutralizing	https://doi.org/10.1016/j.bbamem.2014.08.009
POPG	16:0/18:1	0.696	0.014	333.15	neutralizing	https://doi.org/10.1016/j.bbamem.2014.08.009
SOPG	18:0/18:1	0.629	0.013	293.15	neutralizing	https://doi.org/10.1016/j.bbamem.2014.08.009
SOPG	18:0/18:1	0.643	0.013	303.15	neutralizing	https://doi.org/10.1016/j.bbamem.2014.08.009
SOPG	18:0/18:1	0.676	0.014	323.15	neutralizing	https://doi.org/10.1016/j.bbamem.2014.08.009
SOPG	18:0/18:1	0.69	0.014	333.15	neutralizing	https://doi.org/10.1016/j.bbamem.2014.08.009
DOPG	18:1/18:1	0.679	0.014	293.15	neutralizing	https://doi.org/10.1016/j.bbamem.2014.08.009
DOPG	18:1/18:1	0.691	0.014	303.15	neutralizing	https://doi.org/10.1016/j.bbamem.2014.08.009
DOPG	18:1/18:1	0.711	0.014	323.15	neutralizing	https://doi.org/10.1016/j.bbamem.2014.08.009
DOPG	18:1/18:1	0.717	0.014	333.15	neutralizing	https://doi.org/10.1016/j.bbamem.2014.08.009

DHH

Ref Name	Lipid Tail	DHH [nm]	Uncertainty [nm]	T [K]	NaCl [M]	Ref
DLPC	12:0/12:0	3	0.06	293.15	no	https://doi.org/10.1016/j.bbamem.2011.07.022
DLPC	12:0/12:0	2.96	0.06	303.15	no	https://doi.org/10.1016/j.bbamem.2011.07.022
DLPC	12:0/12:0	2.96	0.06	323.15	no	https://doi.org/10.1016/j.bbamem.2011.07.022
DLPC	12:0/12:0	2.96	0.06	333.15	no	https://doi.org/10.1016/j.bbamem.2011.07.022
DMPC	14:0/14:0	3.45	0.07	303.15	no	https://doi.org/10.1016/j.bbamem.2011.07.022
DMPC	14:0/14:0	3.22	0.07	323.15	no	https://doi.org/10.1016/j.bbamem.2011.07.022
DMPC	14:0/14:0	3.22	0.07	333.15	no	https://doi.org/10.1016/j.bbamem.2011.07.022
DPPC	16:0/16:0	3.86	0.08	323.15	no	https://doi.org/10.1016/j.bbamem.2011.07.022
DPPC	16:0/16:0	3.46	0.07	333.15	no	https://doi.org/10.1016/j.bbamem.2011.07.022
DSPC	18:0/18:0	4.33	0.09	333.15	no	https://doi.org/10.1016/j.bbamem.2011.07.022
MSPC	14:0/18:0	3.57	0.07	323.15	no	https://doi.org/10.3390/sym13081441
SMPC	18:0/14:0	3.48	0.07	323.15	no	https://doi.org/10.3390/sym13081441
PMPC	16:0/14:0	3.39	0.07	323.15	no	https://doi.org/10.3390/sym13081441
DRPC	14:1/14:1	2.96	0.06	303.15	no	https://doi.org/10.1016/j.bpj.2009.06.050
DYPC	16:1/16:1	3.21	0.06	303.15	no	https://doi.org/10.1016/j.bpj.2009.06.050
DOPC	18:1/18:1	3.7	0.07	293.15	no	https://doi.org/10.1039/C6SM02727J
DOPC	18:1/18:1	3.68	0.07	303.15	no	https://doi.org/10.1016/j.bpj.2009.06.050

DGPC	20:1/20:1	3.89	0.08	303.15	no	https://doi.org/10.1016/j.bpj.2009.06.050
DEPC	22:1/22:1	4.55	0.09	303.15	no	https://doi.org/10.1016/j.bpj.2009.06.050
DNPC	24:1/24:1	4.79	0.1	303.15	no	https://doi.org/10.1016/j.bpj.2009.06.050
PSM	d18:1/16:0	3.89	0.08	318.15	no	https://doi.org/10.1021/acs.jpcb.0c03389
PSM	d18:1/16:0	3.78	0.08	328.15	no	https://doi.org/10.1021/acs.jpcb.0c03389
SSM	d18:1/18:0	4	0.08	328.15	no	https://doi.org/10.1021/acs.jpcb.0c03389
SSM	d18:1/18:0	3.94	0.08	338.15	no	https://doi.org/10.1021/acs.jpcb.0c03389
POPC	16:0/18:1	3.74	0.07	293.15	no	https://doi.org/10.1016/j.bbamem.2011.07.022
POPC	16:0/18:1	3.65	0.07	303.15	no	https://doi.org/10.1016/j.bbamem.2011.07.022
POPC	16:0/18:1	3.6	0.07	323.15	no	https://doi.org/10.1016/j.bbamem.2011.07.022
POPC	16:0/18:1	3.59	0.07	333.15	no	https://doi.org/10.1016/j.bbamem.2011.07.022
SOPC	18:0/18:1	3.85	0.08	293.15	no	https://doi.org/10.1016/j.bbamem.2011.07.022
SOPC	18:0/18:1	3.86	0.08	303.15	no	https://doi.org/10.1016/j.bbamem.2011.07.022
SOPC	18:0/18:1	3.7	0.07	323.15	no	https://doi.org/10.1016/j.bbamem.2011.07.022
SOPC	18:0/18:1	3.58	0.07	333.15	no	https://doi.org/10.1016/j.bbamem.2011.07.022
PDPC	16:0/22:6	3.32	0.07	293.15	no	https://doi.org/10.1016/j.chemphyslip.2020.104892
PDPC	16:0/22:6	3.3	0.07	303.15	no	https://doi.org/10.1016/j.chemphyslip.2020.104892
PDPC	16:0/22:6	3.22	0.06	313.15	no	https://doi.org/10.1016/j.chemphyslip.2020.104892
SDPC	18:0/22:6	3.52	0.07	303.15	no	https://doi.org/10.1016/j.chemphyslip.2020.104892
DLPE	12:0/12:0	3.35	0.07	308.15	no	https://doi.org/10.1021/jp511159q
DLPE	12:0/12:0	3.25	0.07	318.15	no	https://doi.org/10.1021/jp511159q
DLPE	12:0/12:0	3.15	0.06	328.15	no	https://doi.org/10.1021/jp511159q
POPE	16:0/18:1	3.83	0.08	308.15	no	https://doi.org/10.1021/jp511159q
POPE	16:0/18:1	3.74	0.07	313.15	no	https://doi.org/10.1021/jp511159q
POPE	16:0/18:1	3.87	0.08	323.15	no	https://doi.org/10.1021/jp511159q
SOPE	18:0/18:1	4.16	0.08	308.15	no	https://doi.org/10.1021/jp511159q
SOPE	18:0/18:1	4.01	0.08	313.15	no	https://doi.org/10.1021/jp511159q
SOPE	18:0/18:1	3.98	0.08	323.15	no	https://doi.org/10.1021/jp511159q
DLPG	12:0/12:0	3	0.06	293.15	neutralizing	https://doi.org/10.1016/j.bbamem.2014.08.009
DLPG	12:0/12:0	2.94	0.06	303.15	neutralizing	https://doi.org/10.1016/j.bbamem.2014.08.009
DLPG	12:0/12:0	2.88	0.06	323.15	neutralizing	https://doi.org/10.1016/j.bbamem.2014.08.009
DLPG	12:0/12:0	2.84	0.06	333.15	neutralizing	https://doi.org/10.1016/j.bbamem.2014.08.009
DMPG	14:0/14:0	3.46	0.07	303.15	neutralizing	https://doi.org/10.1016/j.bbamem.2014.08.009
DMPG	14:0/14:0	3.46	0.07	323.15	neutralizing	https://doi.org/10.1016/j.bbamem.2014.08.009
DMPG	14:0/14:0	3.38	0.07	333.15	neutralizing	https://doi.org/10.1016/j.bbamem.2014.08.009
DPPG	16:0/16:0	3.86	0.08	323.15	neutralizing	https://doi.org/10.1016/j.bbamem.2014.08.009
DPPG	16:0/16:0	3.76	0.08	333.15	neutralizing	https://doi.org/10.1016/j.bbamem.2014.08.009
DSPG	18:0/18:0	4.12	0.08	333.15	neutralizing	https://doi.org/10.1016/j.bbamem.2014.08.009
POPG	16:0/18:1	3.7	0.07	293.15	neutralizing	https://doi.org/10.1016/j.bbamem.2014.08.009
POPG	16:0/18:1	3.66	0.07	303.15	neutralizing	https://doi.org/10.1016/j.bbamem.2014.08.009
POPG	16:0/18:1	3.56	0.07	323.15	neutralizing	https://doi.org/10.1016/j.bbamem.2014.08.009
POPG	16:0/18:1	3.54	0.07	333.15	neutralizing	https://doi.org/10.1016/j.bbamem.2014.08.009
SOPG	18:0/18:1	3.88	0.08	293.15	neutralizing	https://doi.org/10.1016/j.bbamem.2014.08.009
SOPG	18:0/18:1	3.82	0.08	303.15	neutralizing	https://doi.org/10.1016/j.bbamem.2014.08.009

SOPG	18:0/18:1	3.72	0.07	323.15	neutralizing	https://doi.org/10.1016/j.bbamem.2014.08.009
SOPG	18:0/18:1	3.66	0.07	333.15	neutralizing	https://doi.org/10.1016/j.bbamem.2014.08.009
DOPG	18:1/18:1	3.58	0.07	293.15	neutralizing	https://doi.org/10.1016/j.bbamem.2014.08.009
DOPG	18:1/18:1	3.58	0.07	303.15	neutralizing	https://doi.org/10.1016/j.bbamem.2014.08.009
DOPG	18:1/18:1	3.54	0.07	323.15	neutralizing	https://doi.org/10.1016/j.bbamem.2014.08.009
DOPG	18:1/18:1	3.56	0.07	333.15	neutralizing	https://doi.org/10.1016/j.bbamem.2014.08.009

DB

Ref Name	Lipid Tail	DB [nm]	Uncertainty [nm]	T [K]	NaCl [M]	Ref
DLPC	12:0/12:0	3.3	0.07	293.15	no	https://doi.org/10.1016/j.bbamem.2011.07.022
DLPC	12:0/12:0	3.26	0.07	303.15	no	https://doi.org/10.1016/j.bbamem.2011.07.022
DLPC	12:0/12:0	3.1	0.06	323.15	no	https://doi.org/10.1016/j.bbamem.2011.07.022
DLPC	12:0/12:0	3.07	0.06	333.15	no	https://doi.org/10.1016/j.bbamem.2011.07.022
DMPC	14:0/14:0	3.67	0.07	303.15	no	https://doi.org/10.1016/j.bbamem.2011.07.022
DMPC	14:0/14:0	3.52	0.07	323.15	no	https://doi.org/10.1016/j.bbamem.2011.07.022
DMPC	14:0/14:0	3.42	0.07	333.15	no	https://doi.org/10.1016/j.bbamem.2011.07.022
DPPC	16:0/16:0	3.9	0.08	323.15	no	https://doi.org/10.1016/j.bbamem.2011.07.022
DPPC	16:0/16:0	3.81	0.08	333.15	no	https://doi.org/10.1016/j.bbamem.2011.07.022
DSPC	18:0/18:0	4.22	0.08	333.15	no	https://doi.org/10.1016/j.bbamem.2011.07.022
MSPC	14:0/18:0	4.03	0.08	323.15	no	https://doi.org/10.3390/sym13081441
SMPC	18:0/14:0	4.03	0.08	323.15	no	https://doi.org/10.3390/sym13081441
PMPC	16:0/14:0	3.84	0.08	323.15	no	https://doi.org/10.3390/sym13081441
DRPC	14:1/14:1	3.37	0.07	303.15	no	https://doi.org/10.1016/j.bpj.2009.06.050
DYPC	16:1/16:1	3.62	0.07	303.15	no	https://doi.org/10.1016/j.bpj.2009.06.050
DOPC	18:1/18:1	3.94	0.08	293.15	no	https://doi.org/10.1039/C6SM02727J
DOPC	18:1/18:1	3.89	0.08	303.15	no	https://doi.org/10.1016/j.bpj.2009.06.050
DGPC	20:1/20:1	4.25	0.09	303.15	no	https://doi.org/10.1016/j.bpj.2009.06.050
DEPC	22:1/22:1	4.64	0.09	303.15	no	https://doi.org/10.1016/j.bpj.2009.06.050
DNPC	24:1/24:1	5.22	0.1	303.15	no	https://doi.org/10.1016/j.bpj.2009.06.050
PSM	d18:1/16:0	3.84	0.08	318.15	no	https://doi.org/10.1021/acs.jpcb.0c03389
PSM	d18:1/16:0	3.75	0.08	328.15	no	https://doi.org/10.1021/acs.jpcb.0c03389
SSM	d18:1/18:0	3.93	0.08	328.15	no	https://doi.org/10.1021/acs.jpcb.0c03389
SSM	d18:1/18:0	3.81	0.08	338.15	no	https://doi.org/10.1021/acs.jpcb.0c03389
POPC	16:0/18:1	3.98	0.08	293.15	no	https://doi.org/10.1016/j.bbamem.2011.07.022
POPC	16:0/18:1	3.91	0.08	303.15	no	https://doi.org/10.1016/j.bbamem.2011.07.022
POPC	16:0/18:1	3.79	0.08	323.15	no	https://doi.org/10.1016/j.bbamem.2011.07.022
POPC	16:0/18:1	3.77	0.08	333.15	no	https://doi.org/10.1016/j.bbamem.2011.07.022
SOPC	18:0/18:1	4.08	0.08	293.15	no	https://doi.org/10.1016/j.bbamem.2011.07.022
SOPC	18:0/18:1	4	0.08	303.15	no	https://doi.org/10.1016/j.bbamem.2011.07.022
SOPC	18:0/18:1	3.9	0.08	323.15	no	https://doi.org/10.1016/j.bbamem.2011.07.022
SOPC	18:0/18:1	3.85	0.08	333.15	no	https://doi.org/10.1016/j.bbamem.2011.07.022
PDPC	16:0/22:6	3.74	0.07	293.15	no	https://doi.org/10.1016/j.chemphyslip.2020.104892
PDPC	16:0/22:6	3.68	0.07	303.15	no	https://doi.org/10.1016/j.chemphyslip.2020.104892
PDPC	16:0/22:6	3.61	0.07	313.15	no	https://doi.org/10.1016/j.chemphyslip.2020.104892
SDPC	18:0/22:6	3.88	0.08	303.15	no	https://doi.org/10.1016/j.chemphyslip.2020.104892
DLPE	12:0/12:0	3.49	0.07	308.15	no	https://doi.org/10.1021/jp511159q

DLPE	12:0/12:0	3.38	0.07	318.15	no	https://doi.org/10.1021/jp511159q
DLPE	12:0/12:0	3.29	0.07	328.15	no	https://doi.org/10.1021/jp511159q
POPE	16:0/18:1	4.05	0.08	308.15	no	https://doi.org/10.1021/jp511159q
POPE	16:0/18:1	3.99	0.08	313.15	no	https://doi.org/10.1021/jp511159q
POPE	16:0/18:1	3.88	0.08	323.15	no	https://doi.org/10.1021/jp511159q
SOPE	18:0/18:1	4.31	0.09	308.15	no	https://doi.org/10.1021/jp511159q
SOPE	18:0/18:1	4.26	0.09	313.15	no	https://doi.org/10.1021/jp511159q
SOPE	18:0/18:1	4.13	0.08	323.15	no	https://doi.org/10.1021/jp511159q
DLPG	12:0/12:0	3.14	0.06	293.15	neutralizing	https://doi.org/10.1016/j.bbamem.2014.08.009
DLPG	12:0/12:0	3.07	0.06	303.15	neutralizing	https://doi.org/10.1016/j.bbamem.2014.08.009
DLPG	12:0/12:0	2.95	0.06	323.15	neutralizing	https://doi.org/10.1016/j.bbamem.2014.08.009
DLPG	12:0/12:0	2.89	0.06	333.15	neutralizing	https://doi.org/10.1016/j.bbamem.2014.08.009
DMPG	14:0/14:0	3.38	0.07	303.15	neutralizing	https://doi.org/10.1016/j.bbamem.2014.08.009
DMPG	14:0/14:0	3.26	0.07	323.15	neutralizing	https://doi.org/10.1016/j.bbamem.2014.08.009
DMPG	14:0/14:0	3.2	0.06	333.15	neutralizing	https://doi.org/10.1016/j.bbamem.2014.08.009
DPPG	16:0/16:0	3.67	0.07	323.15	neutralizing	https://doi.org/10.1016/j.bbamem.2014.08.009
DPPG	16:0/16:0	3.59	0.07	333.15	neutralizing	https://doi.org/10.1016/j.bbamem.2014.08.009
DSPG	18:0/18:0	3.91	0.08	333.15	neutralizing	https://doi.org/10.1016/j.bbamem.2014.08.009
POPG	16:0/18:1	3.85	0.08	293.15	neutralizing	https://doi.org/10.1016/j.bbamem.2014.08.009
POPG	16:0/18:1	3.76	0.08	303.15	neutralizing	https://doi.org/10.1016/j.bbamem.2014.08.009
POPG	16:0/18:1	3.61	0.07	323.15	neutralizing	https://doi.org/10.1016/j.bbamem.2014.08.009
POPG	16:0/18:1	3.57	0.07	333.15	neutralizing	https://doi.org/10.1016/j.bbamem.2014.08.009
SOPG	18:0/18:1	4.02	0.08	293.15	neutralizing	https://doi.org/10.1016/j.bbamem.2014.08.009
SOPG	18:0/18:1	3.96	0.08	303.15	neutralizing	https://doi.org/10.1016/j.bbamem.2014.08.009
SOPG	18:0/18:1	3.81	0.08	323.15	neutralizing	https://doi.org/10.1016/j.bbamem.2014.08.009
SOPG	18:0/18:1	3.76	0.08	333.15	neutralizing	https://doi.org/10.1016/j.bbamem.2014.08.009
DOPG	18:1/18:1	3.71	0.07	293.15	neutralizing	https://doi.org/10.1016/j.bbamem.2014.08.009
DOPG	18:1/18:1	3.66	0.07	303.15	neutralizing	https://doi.org/10.1016/j.bbamem.2014.08.009
DOPG	18:1/18:1	3.6	0.07	323.15	neutralizing	https://doi.org/10.1016/j.bbamem.2014.08.009
DOPG	18:1/18:1	3.59	0.07	333.15	neutralizing	https://doi.org/10.1016/j.bbamem.2014.08.009

2DC

Ref Name	Lipid Tail	2DC [nm]	Uncertainty [nm]	T [K]	NaCl [M]	Ref
DLPC	12:0/12:0	2.19	0.04	293.15	no	https://doi.org/10.1016/j.bbamem.2011.07.022
DLPC	12:0/12:0	2.17	0.04	303.15	no	https://doi.org/10.1016/j.bbamem.2011.07.022
DLPC	12:0/12:0	2.08	0.04	323.15	no	https://doi.org/10.1016/j.bbamem.2011.07.022
DLPC	12:0/12:0	2.06	0.04	333.15	no	https://doi.org/10.1016/j.bbamem.2011.07.022
DMPC	14:0/14:0	2.57	0.05	303.15	no	https://doi.org/10.1016/j.bbamem.2011.07.022
DMPC	14:0/14:0	2.48	0.05	323.15	no	https://doi.org/10.1016/j.bbamem.2011.07.022
DMPC	14:0/14:0	2.41	0.05	333.15	no	https://doi.org/10.1016/j.bbamem.2011.07.022
DPPC	16:0/16:0	2.85	0.06	323.15	no	https://doi.org/10.1016/j.bbamem.2011.07.022
DPPC	16:0/16:0	2.79	0.06	333.15	no	https://doi.org/10.1016/j.bbamem.2011.07.022
DSPC	18:0/18:0	3.19	0.06	333.15	no	https://doi.org/10.1016/j.bbamem.2011.07.022
MSPC	14:0/18:0	2.91	0.06	323.15	no	https://doi.org/10.3390/sym13081441
SMPC	18:0/14:0	2.92	0.06	323.15	no	https://doi.org/10.3390/sym13081441

PMPC	16:0/14:0	2.7	0.05	323.15	no	https://doi.org/10.3390/sym13081441
DRPC	14:1/14:1	2.34	0.05	303.15	no	https://doi.org/10.1016/j.bpj.2009.06.050
DYPC	16:1/16:1	2.62	0.05	303.15	no	https://doi.org/10.1016/j.bpj.2009.06.050
DOPC	18:1/18:1	3	0.06	293.15	no	https://doi.org/10.1039/C6SM02727J
DOPC	18:1/18:1	2.9	0.06	303.15	no	https://doi.org/10.1016/j.bpj.2009.06.050
DGPC	20:1/20:1	3.26	0.07	303.15	no	https://doi.org/10.1016/j.bpj.2009.06.050
DEPC	22:1/22:1	3.64	0.07	303.15	no	https://doi.org/10.1016/j.bpj.2009.06.050
DNPC	24:1/24:1	4.08	0.08	303.15	no	https://doi.org/10.1016/j.bpj.2009.06.050
PSM	d18:1/16:0	2.93	0.06	318.15	no	https://doi.org/10.1021/acs.jpcb.0c03389
PSM	d18:1/16:0	2.87	0.06	328.15	no	https://doi.org/10.1021/acs.jpcb.0c03389
SSM	d18:1/18:0	3.05	0.06	328.15	no	https://doi.org/10.1021/acs.jpcb.0c03389
SSM	d18:1/18:0	2.97	0.06	338.15	no	https://doi.org/10.1021/acs.jpcb.0c03389
POPC	16:0/18:1	2.92	0.06	293.15	no	https://doi.org/10.1016/j.bbamem.2011.07.022
POPC	16:0/18:1	2.88	0.06	303.15	no	https://doi.org/10.1016/j.bbamem.2011.07.022
POPC	16:0/18:1	2.81	0.06	323.15	no	https://doi.org/10.1016/j.bbamem.2011.07.022
POPC	16:0/18:1	2.8	0.06	333.15	no	https://doi.org/10.1016/j.bbamem.2011.07.022
SOPC	18:0/18:1	3.04	0.06	293.15	no	https://doi.org/10.1016/j.bbamem.2011.07.022
SOPC	18:0/18:1	2.99	0.06	303.15	no	https://doi.org/10.1016/j.bbamem.2011.07.022
SOPC	18:0/18:1	2.93	0.06	323.15	no	https://doi.org/10.1016/j.bbamem.2011.07.022
SOPC	18:0/18:1	2.9	0.06	333.15	no	https://doi.org/10.1016/j.bbamem.2011.07.022
PDPC	16:0/22:6	2.82	0.06	293.15	no	https://doi.org/10.1016/j.chemphyslip.2020.104892
PDPC	16:0/22:6	2.78	0.06	303.15	no	https://doi.org/10.1016/j.chemphyslip.2020.104892
PDPC	16:0/22:6	2.73	0.05	313.15	no	https://doi.org/10.1016/j.chemphyslip.2020.104892
SDPC	18:0/22:6	2.97	0.06	303.15	no	https://doi.org/10.1016/j.chemphyslip.2020.104892
DLPE	12:0/12:0	2.54	0.05	308.15	no	https://doi.org/10.1021/jp511159q
DLPE	12:0/12:0	2.47	0.05	318.15	no	https://doi.org/10.1021/jp511159q
DLPE	12:0/12:0	2.41	0.05	328.15	no	https://doi.org/10.1021/jp511159q
POPE	16:0/18:1	3.21	0.06	308.15	no	https://doi.org/10.1021/jp511159q
POPE	16:0/18:1	3.16	0.06	313.15	no	https://doi.org/10.1021/jp511159q
POPE	16:0/18:1	3.08	0.06	323.15	no	https://doi.org/10.1021/jp511159q
SOPE	18:0/18:1	3.45	0.07	308.15	no	https://doi.org/10.1021/jp511159q
SOPE	18:0/18:1	3.41	0.07	313.15	no	https://doi.org/10.1021/jp511159q
SOPE	18:0/18:1	3.32	0.07	323.15	no	https://doi.org/10.1021/jp511159q
DLPG	12:0/12:0	2.17	0.04	293.15	neutralizing	https://doi.org/10.1016/j.bbamem.2014.08.009
DLPG	12:0/12:0	2.13	0.04	303.15	neutralizing	https://doi.org/10.1016/j.bbamem.2014.08.009
DLPG	12:0/12:0	2.06	0.04	323.15	neutralizing	https://doi.org/10.1016/j.bbamem.2014.08.009
DLPG	12:0/12:0	2.03	0.04	333.15	neutralizing	https://doi.org/10.1016/j.bbamem.2014.08.009
DMPG	14:0/14:0	2.45	0.05	303.15	neutralizing	https://doi.org/10.1016/j.bbamem.2014.08.009
DMPG	14:0/14:0	2.37	0.05	323.15	neutralizing	https://doi.org/10.1016/j.bbamem.2014.08.009
DMPG	14:0/14:0	2.34	0.05	333.15	neutralizing	https://doi.org/10.1016/j.bbamem.2014.08.009
DPPG	16:0/16:0	2.77	0.06	323.15	neutralizing	https://doi.org/10.1016/j.bbamem.2014.08.009
DPPG	16:0/16:0	2.72	0.05	333.15	neutralizing	https://doi.org/10.1016/j.bbamem.2014.08.009
DSPG	18:0/18:0	3.04	0.06	333.15	neutralizing	https://doi.org/10.1016/j.bbamem.2014.08.009
POPG	16:0/18:1	2.91	0.06	293.15	neutralizing	https://doi.org/10.1016/j.bbamem.2014.08.009

POPG	16:0/18:1	2.85	0.06	303.15	neutralizing	https://doi.org/10.1016/j.bbamem.2014.08.009
POPG	16:0/18:1	2.76	0.06	323.15	neutralizing	https://doi.org/10.1016/j.bbamem.2014.08.009
POPG	16:0/18:1	2.74	0.05	333.15	neutralizing	https://doi.org/10.1016/j.bbamem.2014.08.009
SOPG	18:0/18:1	3.1	0.06	293.15	neutralizing	https://doi.org/10.1016/j.bbamem.2014.08.009
SOPG	18:0/18:1	3.05	0.06	303.15	neutralizing	https://doi.org/10.1016/j.bbamem.2014.08.009
SOPG	18:0/18:1	2.95	0.06	323.15	neutralizing	https://doi.org/10.1016/j.bbamem.2014.08.009
SOPG	18:0/18:1	2.92	0.06	333.15	neutralizing	https://doi.org/10.1016/j.bbamem.2014.08.009
DOPG	18:1/18:1	2.85	0.06	293.15	neutralizing	https://doi.org/10.1016/j.bbamem.2014.08.009
DOPG	18:1/18:1	2.82	0.06	303.15	neutralizing	https://doi.org/10.1016/j.bbamem.2014.08.009
DOPG	18:1/18:1	2.79	0.06	323.15	neutralizing	https://doi.org/10.1016/j.bbamem.2014.08.009
DOPG	18:1/18:1	2.78	0.06	333.15	neutralizing	https://doi.org/10.1016/j.bbamem.2014.08.009
